# Supplementary material for: Production of nonulosonic acids in the extracellular polymeric substances of “Candidatus Accumulibacter phosphatis”
Source: Appl Microbiol Biotechnol. 2021 Apr 1;105(8):3327–38. doi: 10.1007/s00253-021-11249-3 (PMC8053191; doi:10.1007/s00253-021-11249-3)
Supplement: Supplementary file 1 — (PDF 2542 kb) [file 253_2021_11249_MOESM1_ESM.pdf]

Applied Microbiology and Biotechnology

Supplementary File

Title: Production of nonulosonic acids in the extracellular polymeric substances of "*Candidatus* Accumulibacter phosphatis"

Sergio Tomás-Martínez*[1], Hugo B.C. Kleikamp[1], Thomas R. Neu[2], Martin Pabst[1], David G. Weissbrodt[1], Mark C.M. van Loosdrecht[1], Yuemei Lin[1]

1 Department of Biotechnology, Delft University of Technology. Van der Maasweg 9,2629 HZ, Delft, The Netherlands

2 Microbiology of Interfaces, Department River Ecology, Helmholtz Centre of Environmental Research – UFZ, Brueckstrasse 3A, 39114, Magdeburg, Germany

*Corresponding Author: Sergio Tomás-Martínez

Address: Department of Biotechnology, Delft University of Technology. Van der Maasweg 9,2629 HZ, Delft, The Netherlands

E-Mail: S.TomasMartinez@tudelft.nl

Supplementary Methods

Shotgun proteomic analysis

Briefly, biomass material was disrupted using beads beating in B-PER reagent (Thermo Scientific)/TEAB buffer (50 mM TEAB, 1% (w/w) NaDOC, adjusted to pH 8.0) buffer. The cell debris was further pelleted and the proteins were precipitated using ice cold acetone. The protein pellet was redissolved in 200 mM ammonium bicarbonate, reduced using DTT and alkylated using iodoacetamide and digested using sequencing grade Trypsin (Promega). An aliquot of approx. 100 ng protein digest was further analyzed by a one dimensional shotgun proteomics approach (Köcher *et al.*, 2012) using an ESAY nano LC 1200 coupled to a QE plus Orbitrap mass spectrometer (Thermo Fisher Scientific, US). The flow rate was maintained at 300 nL/min over a linear gradient from 5% to 30% solvent B over 85 minutes, and finally to 75% B over 25 minutes. Solvent A was H2O containing 0.1% formic acid, and solvent B consisted of 80% acetonitrile in H2O and 0.1% formic acid. The Orbitrap was operated in data depended acquisition mode acquiring peptide signals form 400-1200 m/z at 70K resolution, where the top 10 signals fragmented using a NCE of 30. Raw data were analyzed using PEAKS Studio 8.5 (Bioinformatics Solutions Inc., Canada) allowing 20 ppm parent ion and 0.02 Da fragment mass error tolerance. Search conditions further included considering 3 missed cleavages, carbamidomethylation as fixed and methionine oxidation and N/Q deamidation as variable modifications. Data were matched against a global "Ca. Accumulibacter phosphatis" database (Uniprot, Date, Tax ID 327159). Peptide search included the GPM crap contaminant database and a decoy fusion for determining false discovery rates. Peptide spectrum matches were filtered against 1% false discovery rate (FDR) and protein identifications with 2 or more unique peptides were considered as significant.

Enzymatic quantification

The Sialic Acid Quantitation Kit (Sigma-Aldrich, USA) was used to estimate the content of sialic acids (Neu5Ac as model one) in the enriched "*Ca*. Accumulibacter" biomass. The whole cell assay described in the kit was conducted. Fresh samples were washed with 20 mM Tris-HCl

buffer (pH 7.5) and resuspended in 80 µL of demineralized water. Then 20 µL of sialidase buffer and 1 µL of α(2→3,6,8,9)-neuraminidase were added. This enzyme releases α-2,3-, α-2,6-, α-2,8-, and α-2,9-linked *N*-acetylneuraminic acid from complex carbohydrates. Samples were incubated overnight at 37°C. After incubation, supernatants were collected, volumes were adjusted to 980 µL with Tris-HCl buffer, 20 µL of 0.01 M β-NADH solution was added and the absorbance at 340 nm was measured. Afterwards, 1 µL of *N*-acetylneuraminic acid aldolase and 1 µL of lactic dehydrogenase were added to each sample, and they were incubated at 37 °C for 1 h. Absorbance at 340 nm was measured again after incubation. Sialic acid concentration was calculated using a calibration line of Neu5Ac provided in the kit.

References

Köcher, T., Pichler, P., Swart, R., and Mechtler, K. (2012) Analysis of protein mixtures from whole-cell extracts by single-run nanolc-ms/ms using ultralong gradients. *Nat Protoc* **7**: 882–890.

Supplementary Table

Table S1. Complete list of unique proteins identified in the enrichment of "*Ca*. Accumulibacter"

Accession	-10lgP	Coverage (%)	Intensity	#Unique	Avg. Mass	Description
A0A011P7T1	360.66	52	4.48E8	33	99071	Tetratricopeptide repeat protein OS=Candidatus Accumulibacter sp. BA-93 OX=1454004 GN=AW11_00349 PE=4 SV=1
A0A011PHR0	389.41	57	5.91E9	26	58137	60 kDa chaperonin OS=Candidatus Accumulibacter sp. BA-93 OX=1454004 GN=groL PE=3 SV=1
A0A011REY5	297.41	41	2.15E8	26	98140	Alpha-1 4 glucan phosphorylase OS=Candidatus Accumulibacter sp. BA-93 OX=1454004 GN=malP_1 PE=3 SV=1
A0A369XMT4	339.41	56	3.09E8	25	53303	DUF1254 domain-containing protein OS=Candidatus Accumulibacter phosphatis OX=327160 GN=DVS81_10080 PE=4 SV=1
A0A011QDG6	357.94	33	3.63E8	24	91822	Arylsulfatase OS=Candidatus Accumulibacter sp. BA-93 OX=1454004 GN=atsA_3 PE=4 SV=1
A0A011PHI3	284.21	40	1.82E8	22	73608	Elongation factor G OS=Candidatus Accumulibacter sp. BA-93 OX=1454004 GN=fusA_2 PE=4 SV=1
A0A011PUD2	315.18	46	2.64E8	21	60517	Biotin carboxylase-like protein OS=Candidatus Accumulibacter sp. BA-93 OX=1454004 GN=AW11_00337 PE=4 SV=1
A0A011Q979	294.11	46	1.31E8	21	93546	Alpha-1 4 glucan phosphorylase OS=Candidatus Accumulibacter sp. BA-93 OX=1454004 GN=malP_2 PE=3 SV=1
A0A369XQF8	296.23	35	1.21E9	19	58849	DUF1254 domain-containing protein OS=Candidatus Accumulibacter phosphatis OX=327160 GN=DVS81_10075 PE=4 SV=1
A0A011R415	325.02	78	1.25E9	18	19731	Putative peroxiredoxin OS=Candidatus Accumulibacter sp. BA-93 OX=1454004 GN=AW11_03307 PE=4 SV=1
A0A011QNQ1	294.93	55	3.51E8	18	45856	Methionine gamma-lyase OS=Candidatus Accumulibacter sp. BA-93 OX=1454004 GN=mdeA_1 PE=3 SV=1
A0A011PAD4	272.76	59	3.35E8	18	38695	Calcineurin-like phosphoesterase OS=Candidatus Accumulibacter sp. BA-93 OX=1454004 GN=AW11_03805 PE=4 SV=1
A0A011Q966	282.03	52	6.95E8	17	41779	S-adenosylmethionine synthase OS=Candidatus Accumulibacter sp. BA-93 OX=1454004 GN=metK PE=3 SV=1
A0A011RBG8	313.41	47	3.04E8	16	67932	Outer membrane cobalamin translocator OS=Candidatus Accumulibacter sp. BA-93 OX=1454004 GN=btuB PE=3 SV=1
A0A011QYX9	311.55	54	1.02E8	16	51733	Succinate-semialdehyde dehydrogenase [NADP(+)] GabD OS=Candidatus Accumulibacter sp. BA-93 OX=1454004 GN=gabD_2 PE=3 SV=1
A0A011RIJ4	258.19	41	2.07E8	16	45890	4-aminobutyrate aminotransferase GabT OS=Candidatus Accumulibacter sp. BA-93 OX=1454004 GN=gabT PE=3 SV=1
A0A011QJ04	229.15	36	3.4E7	16	60055	Acyl-CoA dehydrogenase OS=Candidatus Accumulibacter sp. BA-93 OX=1454004 GN=mmgC_2 PE=3 SV=1
A0A011P2V3	220.09	11	2.66E7	16	204683	Tfp pilus assembly protein tip-associated adhesin PilY1 OS=Candidatus Accumulibacter sp. BA-93 OX=1454004 GN=AW11_01622 PE=4 SV=1

A0A011Q4H8	211.87	40	4.85E7	16	42829	Lipoprotein OS=Candidatus Accumulibacter sp. BA-93 OX=1454004 GN=AW11_03944 PE=4 SV=1
A0A011NTH1	305.34	71	1.13E9	15	44455	Isocitrate dehydrogenase [NADP] OS=Candidatus Accumulibacter sp. BA-93 OX=1454004 GN=icd PE=4 SV=1
A0A011QN26	292.14	48	1.53E8	15	62719	Acetolactate synthase OS=Candidatus Accumulibacter sp. BA-93 OX=1454004 GN=ilvI PE=3 SV=1
A0A011NQU5	265.82	50	9.56E7	15	49964	Dihydrolipoyl dehydrogenase OS=Candidatus Accumulibacter sp. BA-93 OX=1454004 GN=lpd PE=3 SV=1
A0A011QMI7	235.59	27	1.24E7	15	77269	Cyanophycin synthetase OS=Candidatus Accumulibacter sp. BA-93 OX=1454004 GN=cphA_1 PE=4 SV=1
A0A011QNJ1	223.62	27	5.97E7	15	70084	Cellobiose 2-epimerase OS=Candidatus Accumulibacter sp. BA-93 OX=1454004 GN=bfce PE=4 SV=1
A0A011QGM4	212.34	42	4.77E7	15	50618	Ribulose bisphosphate carboxylase OS=Candidatus Accumulibacter sp. BA-93 OX=1454004 GN=cbbM PE=3 SV=1
A0A011Q424	232.27	47	3.51E8	14	30880	Glutamate/aspartate periplasmic-binding protein OS=Candidatus Accumulibacter sp. BA-93 OX=1454004 GN=gltI_4 PE=4 SV=1
A0A011PUG6	168.19	19	1.46E7	14	102915	Phosphoenolpyruvate carboxylase OS=Candidatus Accumulibacter sp. BA-93 OX=1454004 GN=ppc PE=3 SV=1
A0A011RDT8	299.53	48	1.5E8	13	62411	Polyphosphate:AMP phosphotransferase OS=Candidatus Accumulibacter sp. BA-93 OX=1454004 GN=AW11_01480 PE=4 SV=1
A0A011QPQ4	278.54	66	9.85E8	13	27731	Sulfate starvation-induced protein 7 OS=Candidatus Accumulibacter sp. BA-93 OX=1454004 GN=fliY_1 PE=3 SV=1
A0A011RI06	274.57	38	4.79E8	13	46557	Putative outer membrane protein OS=Candidatus Accumulibacter sp. BA-93 OX=1454004 GN=AW11_00688 PE=4 SV=1
A0A011QJY0	265.58	47	2.91E8	13	44997	Pyruvate synthase subunit PorA OS=Candidatus Accumulibacter sp. BA-93 OX=1454004 GN=porA PE=4 SV=1
A0A011QD72	254.32	51	2.96E7	13	46600	Tol-Pal system protein TolB OS=Candidatus Accumulibacter sp. BA-93 OX=1454004 GN=tolB PE=3 SV=1
A0A011PTY2	235.31	59	2.43E8	13	22297	50S ribosomal protein L3 OS=Candidatus Accumulibacter sp. BA-93 OX=1454004 GN=rplC PE=3 SV=1
A0A011NQK1	234.41	47	2.05E8	13	29236	2 3 4 5-tetrahydropyridine-2 6-dicarboxylate N-succinyltransferase OS=Candidatus Accumulibacter sp. BA-93 OX=1454004 GN=dapD PE=3 SV=1
A0A011PVX4	335.08	90	2.12E9	12	25992	3-alpha-(Or 20-beta)-hydroxysteroid dehydrogenase OS=Candidatus Accumulibacter sp. BA-92 OX=1454003 GN=fabG3 PE=3 SV=1
A0A011PTB8	335.08	90	2.12E9	12	25992	3-alpha-(Or 20-beta)-hydroxysteroid dehydrogenase OS=Candidatus Accumulibacter sp. BA-93 OX=1454004 GN=fabG3 PE=3 SV=1
A0A011QKN7	312.15	66	7.63E8	12	31079	Succinate--CoA ligase [ADP-forming] subunit alpha OS=Candidatus Accumulibacter sp. BA-93 OX=1454004 GN=sucD PE=3 SV=1
A0A011QCL7	282.32	39	1.55E8	12	82067	NADP-dependent malic enzyme OS=Candidatus Accumulibacter sp. BA-93 OX=1454004 GN=maeB_2 PE=4 SV=1
A0A011PV59	261.90	33	6.76E8	12	41861	Cytochrome c oxidase subunit 2 OS=Candidatus Accumulibacter sp. BA-93 OX=1454004 GN=ctaC PE=3 SV=1
A0A011QJ46	261.16	27	2.8E7	12	155075	DNA-directed RNA polymerase subunit beta' OS=Candidatus Accumulibacter sp. BA-93 OX=1454004 GN=rpoC PE=3 SV=1
A0A011PS88	258.60	52	8.00E+07	12	41731	D-3-phosphoglycerate dehydrogenase OS=Candidatus Accumulibacter sp. BA-93 OX=1454004 GN=serA PE=3 SV=1

A0A011QCV0	248.49	32	4.07E7	12	69890	Peptidylprolyl isomerase OS=Candidatus Accumulibacter sp. BA-93 OX=1454004 GN=ppiD PE=4 SV=1
A0A011PLA5	227.82	27	3.61E7	12	62662	Major tail sheath protein OS=Candidatus Accumulibacter sp. BA-93 OX=1454004 GN=AW11_02177 PE=4 SV=1
A0A011Q5Y4	225.66	20	1.93E7	12	93932	Non-hemolytic phospholipase C OS=Candidatus Accumulibacter sp. BA-93 OX=1454004 GN=plcN PE=4 SV=1
A0A011Q833	205.04	35	1.46E7	12	45808	Hemolysin C OS=Candidatus Accumulibacter sp. BA-93 OX=1454004 GN=tlyC PE=4 SV=1
A0A011QB86	201.66	65	3.07E8	12	16466	50S ribosomal protein L9 OS=Candidatus Accumulibacter sp. BA-93 OX=1454004 GN=rplI PE=3 SV=1
A0A011PCY5	198.42	31	3.05E7	12	50909	Aspartate ammonia-lyase OS=Candidatus Accumulibacter sp. BA-93 OX=1454004 GN=aspA PE=3 SV=1
A0A011PR58	194.07	37	3.81E7	12	45725	Aspartokinase OS=Candidatus Accumulibacter sp. BA-92 OX=1454003 GN=lysC PE=3 SV=1
A0A011Q665	192.10	46	3.05E8	12	27275	Succinate dehydrogenase iron-sulfur subunit OS=Candidatus Accumulibacter sp. BA-93 OX=1454004 GN=sdhB PE=4 SV=1
A0A011RFG3	148.42	18	3.61E6	12	94883	Alanine--tRNA ligase OS=Candidatus Accumulibacter sp. BA-93 OX=1454004 GN=alaS_1 PE=3 SV=1
A0A011QIZ6	424.52	58	6.47E8	11	100003	Pyruvate phosphate dikinase OS=Candidatus Accumulibacter sp. BA-93 OX=1454004 GN=ppdK PE=3 SV=1
A0A011QHN8	308.52	44	7.62E7	11	93647	Periplasmic nitrate reductase OS=Candidatus Accumulibacter sp. BA-93 OX=1454004 GN=napA PE=3 SV=1
A0A011RIN6	282.10	56	1.8E8	11	40360	Polyphosphate kinase 2 OS=Candidatus Accumulibacter sp. BA-93 OX=1454004 GN=AW11_00395 PE=4 SV=1
A0A011RJ45	278.17	57	1.08E9	11	20573	Alkyl hydroperoxide reductase subunit C OS=Candidatus Accumulibacter sp. BA-93 OX=1454004 GN=ahpC PE=4 SV=1
A0A011QNL6	254.19	58	2.29E8	11	36366	Neu5Ac-binding protein OS=Candidatus Accumulibacter sp. BA-93 OX=1454004 GN=siaP PE=4 SV=1
A0A011R5N8	247.39	47	5.7E7	11	43195	Aminotransferase OS=Candidatus Accumulibacter sp. BA-93 OX=1454004 GN=tyrB PE=3 SV=1
A0A011RHD2	238.73	36	2.22E7	11	44896	Urea ABC transporter urea binding protein OS=Candidatus Accumulibacter sp. BA-93 OX=1454004 GN=AW11_00491 PE=4 SV=1
A0A011RBZ8	230.19	42	2.21E7	11	45484	Glucose-1-phosphate adenylyltransferase OS=Candidatus Accumulibacter sp. BA-93 OX=1454004 GN=glgC_1 PE=3 SV=1
A0A011Q992	215.26	64	2.39E8	11	23672	Putative pit accessory protein OS=Candidatus Accumulibacter sp. BA-93 OX=1454004 GN=AW11_03387 PE=4 SV=1
A0A011NRT4	211.35	63	1.34E8	11	24132	Putative peroxiredoxin OS=Candidatus Accumulibacter sp. BA-93 OX=1454004 GN=tsaA_2 PE=4 SV=1
A0A011QJ52	202.40	93	2.72E8	11	10363	Probable Fe(2+)-trafficking protein OS=Candidatus Accumulibacter sp. BA-93 OX=1454004 GN=AW11_01478 PE=3 SV=1
A0A011QB99	197.41	23	1.05E8	11	47173	Glucose-1-phosphate adenylyltransferase OS=Candidatus Accumulibacter sp. BA-93 OX=1454004 GN=glgC_2 PE=3 SV=1
A0A011QEU5	165.87	17	1.05E7	11	101679	Protease3 OS=Candidatus Accumulibacter sp. BA-93 OX=1454004 GN=AW11_02479 PE=3 SV=1
A0A011NUL8	380.76	77	9.88E8	10	40489	Beta-ketothiolase BktB OS=Candidatus Accumulibacter sp. BA-93 OX=1454004 GN=bktB PE=3 SV=1
A0A011QNK3	317.25	57	1.67E8	10	43855	Phosphoglycerate kinase OS=Candidatus Accumulibacter sp. BA-93 OX=1454004 GN=pgk PE=3 SV=1
A0A011PNK7	266.35	32	6.73E6	10	157784	DNA-directed RNA polymerase subunit beta OS=Candidatus Accumulibacter sp. BA-93 OX=1454004 GN=rpoB PE=3 SV=1
A0A011QIV3	257.27	55	7.09E7	10	31187	Elongation factor Ts OS=Candidatus Accumulibacter sp. BA-93 OX=1454004 GN=tsf PE=3 SV=1

A0A011P7B6	254.75	59	1.2E8	10	30144	50S ribosomal protein L2 OS=Candidatus Accumulibacter sp. BA-93 OX=1454004 GN=rplB PE=3 SV=1
A0A011RIP0	200.57	64	4.88E8	10	10681	Cytochrome c552 OS=Candidatus Accumulibacter sp. BA-93 OX=1454004 GN=cyt PE=4 SV=1
A0A011PKS8	192.55	39	6.12E7	10	28249	Succinate dehydrogenase iron-sulfur subunit OS=Candidatus Accumulibacter sp. BA-92 OX=1454003 GN=frdB PE=3 SV=1
A0A011Q7P3	190.98	46	8.69E7	10	26307	Putative phospholipid-binding lipoprotein MlaA OS=Candidatus Accumulibacter sp. BA-93 OX=1454004 GN=mlaA PE=4 SV=1
A0A011PTX2	182.59	66	6.02E7	10	13282	50S ribosomal protein L14 OS=Candidatus Accumulibacter sp. BA-93 OX=1454004 GN=rplN PE=3 SV=1
A0A011QIG7	175.16	12	2.78E7	10	104615	Protein translocase subunit SecA OS=Candidatus Accumulibacter sp. BA-93 OX=1454004 GN=secA PE=3 SV=1
A0A011RGS8	172.61	71	1.61E8	10	17812	Acetolactate synthase isozyme 3 small subunit OS=Candidatus Accumulibacter sp. BA-93 OX=1454004 GN=ilvH PE=4 SV=1
A0A011PLT8	171.22	45	5.48E7	10	29584	Site-determining protein OS=Candidatus Accumulibacter sp. BA-93 OX=1454004 GN=minD PE=3 SV=1
A0A011PFK1	164.83	34	2.88E7	10	39748	Outer membrane protein assembly factor BamB OS=Candidatus Accumulibacter sp. BA-93 OX=1454004 GN=bamB PE=3 SV=1
A0A011QMQ4	124.36	14	9.64E6	10	79950	Glycogen debranching enzyme OS=Candidatus Accumulibacter sp. BA-93 OX=1454004 GN=glgX_1 PE=3 SV=1
A0A011P2Z2	373.54	71	5.21E8	9	58842	NAD-dependent dihydropyrimidine dehydrogenase subunit PreT OS=Candidatus Accumulibacter sp. BA-93 OX=1454004 GN=preT PE=4 SV=1
A0A011QI84	340.26	57	2.26E7	9	77440	Elongation factor G OS=Candidatus Accumulibacter sp. BA-93 OX=1454004 GN=fusA_1 PE=3 SV=1
A0A011P5Q9	299.14	45	3.9E8	9	38803	Leucine- isoleucine- valine- threonine- and alanine-binding protein OS=Candidatus Accumulibacter sp. BA-93 OX=1454004 GN=braC_2 PE=4 SV=1
A0A011R267	285.04	91	1.73E9	9	18873	Phasin family protein OS=Candidatus Accumulibacter sp. BA-93 OX=1454004 GN=AW11_03612 PE=4 SV=1
A0A011PPG8	265.06	57	6.97E7	9	52498	Citrate lyase beta subunit OS=Candidatus Accumulibacter sp. BA-93 OX=1454004 GN=AW11_01665 PE=3 SV=1
A0A011P6B6	249.60	34	9.00E+06	9	91897	Cyanophycin synthetase OS=Candidatus Accumulibacter sp. BA-93 OX=1454004 GN=cphA_2 PE=4 SV=1
A0A369XP17	235.09	80	8.23E8	9	17064	VOC family protein OS=Candidatus Accumulibacter phosphatis OX=327160 GN=DVS81_12500 PE=4 SV=1
A0A011Q239	235.09	79	8.23E8	9	17151	Methylmalonyl-CoA epimerase OS=Candidatus Accumulibacter sp. BA-92 OX=1454003 GN=AW10_00048 PE=4 SV=1
A0A011P6W0	225.55	50	2.99E8	9	25311	Uncharacterized protein OS=Candidatus Accumulibacter sp. BA-93 OX=1454004 GN=AW11_00566 PE=4 SV=1
A0A011PSQ9	209.06	34	7.27E6	9	55186	2-isopropylmalate synthase OS=Candidatus Accumulibacter sp. BA-93 OX=1454004 GN=leuA_1 PE=3 SV=1
A0A011PPD9	206.62	33	1.93E8	9	28887	Uncharacterized protein OS=Candidatus Accumulibacter sp. BA-93 OX=1454004 GN=AW11_01640 PE=4 SV=1
A0A011RGH5	193.02	22	2.84E7	9	47239	Polyphosphate kinase 2 OS=Candidatus Accumulibacter sp. BA-93 OX=1454004 GN=AW11_01023 PE=4 SV=1
A0A011QG52	187.36	28	3.62E7	9	47489	UDP-glucose 6-dehydrogenase OS=Candidatus Accumulibacter sp. BA-93 OX=1454004 GN=algD PE=3 SV=1

A0A011P853	159.11	28	3.64E7	9	34511	Uncharacterized protein OS=Candidatus Accumulibacter sp. BA-93 OX=1454004 GN=AW11_00187 PE=4 SV=1
A0A011P2W3	146.80	12	4.72E6	9	105724	Isoleucine--tRNA ligase OS=Candidatus Accumulibacter sp. BA-93 OX=1454004 GN=ileS PE=3 SV=1
A0A011NS27	143.87	37	3.54E7	9	23806	30S ribosomal protein S4 OS=Candidatus Accumulibacter sp. BA-92 OX=1454003 GN=rpsD PE=3 SV=1
A0A011QNF0	143.87	37	3.54E7	9	23804	30S ribosomal protein S4 OS=Candidatus Accumulibacter sp. BA-93 OX=1454004 GN=rpsD PE=3 SV=1
A0A011P5R4	296.03	36	9.35E7	8	63358	Arylsulfatase OS=Candidatus Accumulibacter sp. BA-93 OX=1454004 GN=atsA_1 PE=4 SV=1
A0A011Q9F6	283.70	43	6.16E8	8	51662	Glutamine synthetase OS=Candidatus Accumulibacter sp. BA-93 OX=1454004 GN=AW11_03365 PE=3 SV=1
A0A011QNY0	267.42	71	1.19E8	8	28721	Peptidylprolyl isomerase OS=Candidatus Accumulibacter sp. BA-93 OX=1454004 GN=AW11_00313 PE=4 SV=1
A0A011PR99	260.55	47	6.34E7	8	46312	sn-glycerol-3-phosphate-binding periplasmic protein UgpB OS=Candidatus Accumulibacter sp. BA-93 OX=1454004 GN=ugpB PE=4 SV=1
A0A011RG95	245.45	42	1.02E8	8	64154	Acyl-CoA dehydrogenase OS=Candidatus Accumulibacter sp. BA-93 OX=1454004 GN=mmgC_1 PE=3 SV=1
A0A011QJL7	232.48	20	5.77E6	8	170018	NAD-specific glutamate dehydrogenase OS=Candidatus Accumulibacter sp. BA-93 OX=1454004 GN=gdhB PE=4 SV=1
A0A011QLQ4	227.53	26	1.67E8	8	39674	Phosphoserine transaminase OS=Candidatus Accumulibacter sp. BA-93 OX=1454004 GN=serC PE=3 SV=1
A0A011P6S7	213.25	26	5.89E7	8	54828	Uncharacterized protein OS=Candidatus Accumulibacter sp. BA-93 OX=1454004 GN=AW11_00530 PE=4 SV=1
A0A011PV16	210.30	54	3.84E8	8	12077	Peptidyl-prolyl cis-trans isomerase OS=Candidatus Accumulibacter sp. BA-93 OX=1454004 GN=fbp_1 PE=3 SV=1
A0A011QCR7	207.24	39	6.68E7	8	26189	Cytochrome c1 OS=Candidatus Accumulibacter sp. BA-93 OX=1454004 GN=petC PE=4 SV=1
A0A011RF33	203.91	23	8.53E6	8	67714	2-oxoglutarate oxidoreductase subunit KorA OS=Candidatus Accumulibacter sp. BA-93 OX=1454004 GN=korA PE=4 SV=1
A0A011PM08	201.78	36	1.94E8	8	33078	Peptidylprolyl isomerase OS=Candidatus Accumulibacter sp. BA-93 OX=1454004 GN=AW11_02096 PE=4 SV=1
A0A011N237	201.69	68	8.09E8	8	9863	DNA-binding protein HU OS=Candidatus Accumulibacter sp. BA-92 OX=1454003 GN=hup_2 PE=3 SV=1
A0A011QEA1	201.69	68	8.09E8	8	9891	DNA-binding protein HU OS=Candidatus Accumulibacter sp. BA-93 OX=1454004 GN=hup PE=3 SV=1
A0A011QPI1	198.32	49	2.16E8	8	16929	ATP synthase subunit b OS=Candidatus Accumulibacter sp. BA-93 OX=1454004 GN=atpF_1 PE=3 SV=1
A0A011QJJ3	185.91	52	1.18E8	8	11834	Putative zinc finger/helix-turn-helix protein YgiT family OS=Candidatus Accumulibacter sp. BA-93 OX=1454004 GN=AW11_01725 PE=4 SV=1
A0A011Q9D1	180.68	21	7.89E7	8	49454	NAD(P) transhydrogenase subunit beta OS=Candidatus Accumulibacter sp. BA-93 OX=1454004 GN=pntB PE=3 SV=1
A0A011PMN2	179.01	29	1.52E7	8	38096	N-ethylmaleimide reductase OS=Candidatus Accumulibacter sp. BA-93 OX=1454004 GN=nemA_2 PE=4 SV=1
A0A011R7J2	175.76	22	2.3E7	8	45811	ATP-dependent Clp protease ATP-binding subunit ClpX OS=Candidatus Accumulibacter sp. BA-93 OX=1454004 GN=clpX_1 PE=3 SV=1
A0A011PNM9	175.35	27	3.68E7	8	41271	D-alanyl-D-alanine carboxypeptidase DacC OS=Candidatus Accumulibacter sp. BA-93 OX=1454004 GN=dacC PE=3 SV=1

A0A011P8P1	173.58	45	8.55E7	8	22406	Cytochrome c4 OS=Candidatus Accumulibacter sp. BA-93 OX=1454004 GN=cc4_1 PE=4 SV=1
A0A011QAI9	173.28	16	6.74E7	8	58569	4-alpha-glucanotransferase OS=Candidatus Accumulibacter sp. BA-93 OX=1454004 GN=malQ PE=3 SV=1
A0A011PHN2	172.99	43	9.14E7	8	23145	Stringent starvation protein A OS=Candidatus Accumulibacter sp. BA-93 OX=1454004 GN=sspA PE=4 SV=1
A0A011QJ16	158.92	26	1.31E7	8	43835	Putative plant photosystem II stability/assembly factor OS=Candidatus Accumulibacter sp. BA-93 OX=1454004 GN=AW11_01676 PE=4 SV=1
A0A011Q956	155.01	36	2.13E7	8	25194	Pilus assembly protein PilO OS=Candidatus Accumulibacter sp. BA-93 OX=1454004 GN=AW11_03347 PE=4 SV=1
A0A011P1J6	148.32	28	2.00E+06	8	39931	dTDP-glucose 4 6-dehydratase OS=Candidatus Accumulibacter sp. BA-93 OX=1454004 GN=rfbB_2 PE=3 SV=1
A0A011QP52	141.76	27	2.87E6	8	38552	Twitching mobility protein OS=Candidatus Accumulibacter sp. BA-93 OX=1454004 GN=pilT_2 PE=4 SV=1
A0A011PTT1	141.57	33	0.00E+00	8	29298	Inositol-1-monophosphatase OS=Candidatus Accumulibacter sp. BA-93 OX=1454004 GN=suhB_1 PE=3 SV=1
A0A011RJ08	138.53	21	2.89E6	8	48571	Uncharacterized protein OS=Candidatus Accumulibacter sp. BA-93 OX=1454004 GN=AW11_00213 PE=4 SV=1
A0A011P7S6	132.85	10	5.1E5	8	105139	Lysine decarboxylase constitutive OS=Candidatus Accumulibacter sp. BA-93 OX=1454004 GN=ldcC PE=4 SV=1
A0A011PJ06	124.24	39	1.23E6	8	29660	Diaminopimelate epimerase OS=Candidatus Accumulibacter sp. BA-93 OX=1454004 GN=dapF PE=3 SV=1
A0A011R6R8	356.91	58	3.97E8	7	49879	Enolase OS=Candidatus Accumulibacter sp. BA-93 OX=1454004 GN=eno_1 PE=3 SV=1
A0A011RB95	344.26	67	1.74E8	7	35942	Glyceraldehyde-3-phosphate dehydrogenase OS=Candidatus Accumulibacter sp. BA-93 OX=1454004 GN=gapA PE=3 SV=1
A0A011RJ56	342.68	57	7.24E8	7	55549	ATP synthase subunit alpha OS=Candidatus Accumulibacter sp. BA-93 OX=1454004 GN=atpA_1 PE=3 SV=1
A0A011R5I3	306.99	63	1.64E8	7	58066	Phosphoglucomutase OS=Candidatus Accumulibacter sp. BA-93 OX=1454004 GN=pgm PE=3 SV=1
A0A011Q808	294.94	31	4.8E7	7	95508	Uncharacterized protein OS=Candidatus Accumulibacter sp. BA-93 OX=1454004 GN=AW11_03623 PE=4 SV=1
A0A011QPG3	274.26	52	1.05E8	7	28623	Uncharacterized protein OS=Candidatus Accumulibacter sp. BA-93 OX=1454004 GN=AW11_00001 PE=4 SV=1
A0A011PHR5	263.04	37	1.63E8	7	49705	Trigger factor OS=Candidatus Accumulibacter sp. BA-93 OX=1454004 GN=tig PE=3 SV=1
A0A011PT03	255.05	34	1.58E7	7	83159	NADH-quinone oxidoreductase OS=Candidatus Accumulibacter sp. BA-93 OX=1454004 GN=nqo3 PE=3 SV=1
A0A011QBU4	254.28	50	1.3E8	7	41450	Extracytoplasmic solute receptor protein YiaO OS=Candidatus Accumulibacter sp. BA-93 OX=1454004 GN=yiaO_2 PE=3 SV=1
A0A011Q3Y9	243.70	43	1.1E8	7	28446	Cobalt-precorrin-2 C(20)-methyltransferase OS=Candidatus Accumulibacter sp. BA-93 OX=1454004 GN=cbiL PE=4 SV=1
A0A011RHJ6	237.87	21	1.81E8	7	55728	Methylmalonyl-CoA carboxyltransferase 12S subunit OS=Candidatus Accumulibacter sp. BA-93 OX=1454004 GN=AW11_00542 PE=4 SV=1

A0A011P012	237.87	21	1.81E8	7	55728	Methylmalonyl-CoA carboxyltransferase 12S subunit OS=Candidatus Accumulibacter sp. BA-92 OX=1454003 GN=AW10_01549 PE=4 SV=1
A0A011RJ34	237.18	38	8.44E7	7	41806	Beta sliding clamp OS=Candidatus Accumulibacter sp. BA-93 OX=1454004 GN=dnaN PE=3 SV=1
A0A011RCA4	225.07	40	8.85E8	7	41916	Outer membrane porin protein 32 OS=Candidatus Accumulibacter sp. BA-93 OX=1454004 GN=AW11_01957 PE=4 SV=1
A0A011PPH3	224.46	26	1.62E8	7	62841	Crotonyl-CoA reductase OS=Candidatus Accumulibacter sp. BA-93 OX=1454004 GN=ccr PE=4 SV=1
A0A011QK57	223.72	44	3.03E7	7	44363	Capsular glucan synthase OS=Candidatus Accumulibacter sp. BA-93 OX=1454004 GN=glgA_1 PE=4 SV=1
A0A011P054	218.91	40	2.43E8	7	27839	Putative enoyl-CoA hydratase echA8 OS=Candidatus Accumulibacter sp. BA-92 OX=1454003 GN=echA8_2 PE=3 SV=1
A0A011P2W7	212.15	39	1.8E8	7	22884	Cbb3-type cytochrome c oxidase subunit II OS=Candidatus Accumulibacter sp. BA-93 OX=1454004 GN=AW11_01637 PE=4 SV=1
A0A011R568	210.40	31	5.17E7	7	43217	3-oxoacyl-[acyl-carrier-protein] synthase 2 OS=Candidatus Accumulibacter sp. BA-93 OX=1454004 GN=fabF PE=3 SV=1
A0A011RJI2	198.55	53	9.53E7	7	16080	Arabinose 5-phosphate isomerase KdsD OS=Candidatus Accumulibacter sp. BA-93 OX=1454004 GN=kdsD_1 PE=4 SV=1
A0A011QP28	198.50	63	1.92E8	7	11444	50S ribosomal protein L24 OS=Candidatus Accumulibacter sp. BA-93 OX=1454004 GN=rplX PE=3 SV=1
A0A011P6P4	195.76	73	2.5E8	7	15262	(3R)-hydroxyacyl-ACP dehydratase subunit HadB OS=Candidatus Accumulibacter sp. BA-93 OX=1454004 GN=AW11_00500 PE=4 SV=1
A0A011P7R3	188.37	56	1.55E7	7	16210	Uncharacterized protein OS=Candidatus Accumulibacter sp. BA-93 OX=1454004 GN=AW11_00319 PE=4 SV=1
A0A011P5S9	185.22	41	1.32E8	7	20448	Inorganic pyrophosphatase OS=Candidatus Accumulibacter sp. BA-93 OX=1454004 GN=ppa_1 PE=3 SV=1
A0A011P3M7	185.22	41	1.32E8	7	20409	Inorganic pyrophosphatase OS=Candidatus Accumulibacter sp. BA-92 OX=1454003 GN=ppa_2 PE=3 SV=1
A0A011RCS0	185.09	53	4.03E7	7	23850	50S ribosomal protein L1 OS=Candidatus Accumulibacter sp. BA-93 OX=1454004 GN=rplA PE=3 SV=1
A0A011RHE4	180.56	42	3.53E8	7	16278	(3R)-hydroxyacyl-ACP dehydratase subunit HadC OS=Candidatus Accumulibacter sp. BA-93 OX=1454004 GN=AW11_00501 PE=4 SV=1
A0A011PAD9	173.28	19	1.16E7	7	41473	Astacin (Peptidase family M12A) OS=Candidatus Accumulibacter sp. BA-93 OX=1454004 GN=AW11_03810 PE=4 SV=1
A0A011QNF7	172.09	53	2.29E7	7	15127	50S ribosomal protein L15 OS=Candidatus Accumulibacter sp. BA-93 OX=1454004 GN=rplO PE=3 SV=1
A0A011P220	171.04	41	5.79E7	7	17694	30S ribosomal protein S7 OS=Candidatus Accumulibacter sp. BA-93 OX=1454004 GN=rpsG PE=3 SV=1
A0A011QB11	170.80	17	1.71E6	7	63792	Magnesium chelatase subunit D OS=Candidatus Accumulibacter sp. BA-93 OX=1454004 GN=AW11_03094 PE=4 SV=1
A0A369XLB7	161.36	35	1.72E7	7	25714	UMP kinase OS=Candidatus Accumulibacter phosphatis OX=327160 GN=DVS81_19065 PE=4 SV=1
A0A011QPZ2	160.68	24	6.54E6	7	34404	Uncharacterized protein OS=Candidatus Accumulibacter sp. BA-93 OX=1454004 GN=AW11_00204 PE=4 SV=1
A0A011PKJ5	157.69	24	4.07E6	7	49278	Tryptophan synthase beta chain OS=Candidatus Accumulibacter sp. BA-93 OX=1454004 GN=trpB_1 PE=3 SV=1
A0A011N547	153.37	22	9.59E6	7	44763	Cell division protein FtsA OS=Candidatus Accumulibacter sp. BA-92 OX=1454003 GN=ftsA PE=3 SV=1

A0A369XSP8	153.37	22	9.59E6	7	44749	Cell division protein FtsA OS=Candidatus Accumulibacter phosphatis OX=327160 GN=ftsA PE=4 SV=1
A0A011R5X8	153.37	22	9.59E6	7	44777	Cell division protein FtsA OS=Candidatus Accumulibacter sp. BA-93 OX=1454004 GN=ftsA PE=3 SV=1
A0A011NU42	148.31	11	7.12E6	7	115413	Efflux pump membrane transporter OS=Candidatus Accumulibacter sp. BA-93 OX=1454004 GN=bepE_1 PE=3 SV=1
A0A369XNI6	147.80	39	2.81E8	7	11584	H-NS histone family protein OS=Candidatus Accumulibacter phosphatis OX=327160 GN=DVS81_19300 PE=4 SV=1
A0A011QNV8	147.67	29	2.6E6	7	29983	Outer membrane lipoprotein-sorting protein OS=Candidatus Accumulibacter sp. BA-93 OX=1454004 GN=AW11_00638 PE=4 SV=1
A0A011RC48	147.10	22	6.87E6	7	45302	Tryptophan--tRNA ligase OS=Candidatus Accumulibacter sp. BA-93 OX=1454004 GN=trpS PE=3 SV=1
A0A011RA32	143.17	33	8.49E6	7	23942	3-isopropylmalate dehydratase small subunit OS=Candidatus Accumulibacter sp. BA-93 OX=1454004 GN=leuD PE=3 SV=1
A0A011NWI8	139.67	17	5.29E6	7	49897	Cytochrome b OS=Candidatus Accumulibacter sp. BA-93 OX=1454004 GN=petB PE=3 SV=1
A0A011QIZ9	138.60	21	1.81E7	7	39223	Bifunctional aldehyde dehydrogenase/enoyl-CoA hydratase OS=Candidatus Accumulibacter sp. BA-93 OX=1454004 GN=AW11_01661 PE=4 SV=1
A0A011QN92	135.19	26	3.76E6	7	40866	2-aminoethylphosphonate--pyruvate transaminase OS=Candidatus Accumulibacter sp. BA-93 OX=1454004 GN=phnW PE=3 SV=1
A0A011PQ48	126.02	30	4.54E6	7	33599	Glucose-1-phosphate thymidylyltransferase OS=Candidatus Accumulibacter sp. BA-93 OX=1454004 GN=rmlA1 PE=3 SV=1
A0A011NU25	122.15	13	5.65E6	7	69709	Uncharacterized protein OS=Candidatus Accumulibacter sp. BA-93 OX=1454004 GN=AW11_03030 PE=4 SV=1
A0A011QGL9	114.25	26	1.68E6	7	38359	Phosphoribosylaminoimidazole-succinocarboxamide synthase OS=Candidatus Accumulibacter sp. BA-93 OX=1454004 GN=purC PE=3 SV=1
A0A011QQ48	369.87	58	1.4E8	6	38895	Poly(R)-hydroxyalkanoic acid synthase class III PhaE subunit OS=Candidatus Accumulibacter sp. BA-92 OX=1454003 GN=AW10_01588 PE=4 SV=1
A0A011P2K2	369.27	61	1.55E8	6	69852	Chaperone protein DnaK OS=Candidatus Accumulibacter sp. BA-93 OX=1454004 GN=dnaK_3 PE=2 SV=1
A0A011R2H3	344.77	49	2.01E8	6	75311	Polyribonucleotide nucleotidyltransferase OS=Candidatus Accumulibacter sp. BA-93 OX=1454004 GN=pnp PE=3 SV=1
A0A011NPE7	302.96	37	1.72E7	6	105964	2-oxoglutarate dehydrogenase E1 component OS=Candidatus Accumulibacter sp. BA-93 OX=1454004 GN=sucA PE=4 SV=1
A0A011QIK0	298.36	50	1.15E8	6	55894	Putative propionyl-CoA carboxylase beta chain 5 OS=Candidatus Accumulibacter sp. BA-93 OX=1454004 GN=accD5 PE=4 SV=1
A0A011Q948	267.61	50	5.93E7	6	52194	Glutamate synthase [NADPH] small chain OS=Candidatus Accumulibacter sp. BA-93 OX=1454004 GN=gltB_1 PE=4 SV=1
A0A011RDB1	264.25	35	2.33E7	6	58876	Phosphoglycerate mutase (2 3-diphosphoglycerate-independent) OS=Candidatus Accumulibacter sp. BA-93 OX=1454004 GN=gpmI PE=3 SV=1
A0A011QI39	253.27	25	7.84E7	6	93303	Arylsulfatase OS=Candidatus Accumulibacter sp. BA-93 OX=1454004 GN=atsA_2 PE=4 SV=1
A0A011NNQ6	249.06	20	2.27E8	6	82496	K(+)-insensitive pyrophosphate-energized proton pump OS=Candidatus Accumulibacter sp. BA-93 OX=1454004 GN=hppA_2 PE=3 SV=1

A0A011NSM1	243.76	21	0.00E+00	6	169969	Ferredoxin-dependent glutamate synthase 1 OS=Candidatus Accumulibacter sp. BA-93 OX=1454004 GN=gltB_2 PE=4 SV=1
A0A011R5E8	235.15	68	2.54E8	6	15374	Nucleoside diphosphate kinase OS=Candidatus Accumulibacter sp. BA-93 OX=1454004 GN=ndk PE=3 SV=1
A0A011PQV5	232.10	39	2.85E8	6	29292	Curli production assembly/transport component CsgG OS=Candidatus Accumulibacter sp. BA-93 OX=1454004 GN=AW11_01296 PE=4 SV=1
A0A011PPG1	230.58	44	2.01E8	6	21415	Pyruvate synthase subunit PorC OS=Candidatus Accumulibacter sp. BA-93 OX=1454004 GN=porC PE=4 SV=1
A0A011P1C8	228.94	21	2.21E7	6	102278	Translation initiation factor IF-2 OS=Candidatus Accumulibacter sp. BA-93 OX=1454004 GN=infB PE=3 SV=1
A0A011NZD8	216.76	21	1.25E7	6	72066	Threonine--tRNA ligase OS=Candidatus Accumulibacter sp. BA-93 OX=1454004 GN=thrS PE=3 SV=1
C7RJE2	213.88	57	1.37E8	6	11868	30S ribosomal protein S10 OS=Accumulibacter phosphatis (strain UW-1) OX=522306 GN=rpsJ PE=3 SV=1
A0A369XR18	213.88	57	1.37E8	6	11896	30S ribosomal protein S10 OS=Candidatus Accumulibacter phosphatis OX=327160 GN=DVS81_09325 PE=4 SV=1
A0A011PTZ3	213.88	57	1.37E8	6	11882	30S ribosomal protein S10 OS=Candidatus Accumulibacter sp. BA-92 OX=1454003 GN=rpsJ PE=3 SV=1
A0A011PMU2	213.88	57	1.37E8	6	11882	30S ribosomal protein S10 OS=Candidatus Accumulibacter sp. BA-94 OX=1454005 GN=rpsJ PE=3 SV=1
A0A011QNI1	213.88	57	1.37E8	6	11882	30S ribosomal protein S10 OS=Candidatus Accumulibacter sp. BA-93 OX=1454004 GN=rpsJ PE=3 SV=1
A0A084Y3B2	213.88	57	1.37E8	6	11876	30S ribosomal protein S10 OS=Candidatus Accumulibacter sp. SK-01 OX=1457154 GN=rpsJ PE=3 SV=1
A0A011QEW4	213.88	57	1.37E8	6	11860	30S ribosomal protein S10 OS=Candidatus Accumulibacter sp. BA-94 OX=1454005 GN=rpsJ PE=3 SV=1
A0A080MAM7	213.88	57	1.37E8	6	11876	30S ribosomal protein S10 OS=Candidatus Accumulibacter sp. SK-02 OX=1453999 GN=rpsJ PE=3 SV=1
A0A1Q3VPD5	213.88	57	1.37E8	6	11879	30S ribosomal protein S10 OS=Candidatus Accumulibacter sp. 66-26 OX=1895689 GN=rpsJ PE=3 SV=1
A0A011RIL3	209.67	44	2.04E7	6	45748	Argininosuccinate synthase OS=Candidatus Accumulibacter sp. BA-93 OX=1454004 GN=argG PE=3 SV=1
A0A011RDE0	196.35	94	1.82E8	6	8900	RNA-binding protein Hfq OS=Candidatus Accumulibacter sp. BA-93 OX=1454004 GN=hfq PE=3 SV=1
A0A011QDN8	195.38	55	2.38E7	6	17463	Single-stranded DNA-binding protein OS=Candidatus Accumulibacter sp. BA-93 OX=1454004 GN=ssb_2 PE=3 SV=1
A0A011R551	186.19	35	4.9E7	6	21645	Uncharacterized protein OS=Candidatus Accumulibacter sp. BA-93 OX=1454004 GN=AW11_03041 PE=4 SV=1
A0A011QFC2	186.01	27	0.00E+00	6	40625	Aspartate-semialdehyde dehydrogenase OS=Candidatus Accumulibacter sp. BA-93 OX=1454004 GN=asd PE=3 SV=1
A0A011PTB9	184.24	17	2.18E8	6	34587	UDP-glucose 4-epimerase OS=Candidatus Accumulibacter sp. SK-12 OX=1454001 GN=AW08_00107 PE=4 SV=1
A0A011P779	182.28	58	1.13E8	6	13076	Enamine/imine deaminase OS=Candidatus Accumulibacter sp. BA-93 OX=1454004 GN=yabJ PE=4 SV=1
A0A011QEF0	180.63	21	6.12E7	6	46821	Outer membrane protein P1 OS=Candidatus Accumulibacter sp. BA-93 OX=1454004 GN=ompP1 PE=4 SV=1
A0A011RIR5	178.82	16	0.00E+00	6	76501	Methyl-accepting chemotaxis protein 4 OS=Candidatus Accumulibacter sp. BA-93 OX=1454004 GN=mcp4_1 PE=4 SV=1
A0A369XQV5	176.10	51	7.11E7	6	13723	30S ribosomal protein S11 OS=Candidatus Accumulibacter phosphatis OX=327160 GN=DVS81_09200 PE=4 SV=1

A0A011P798	176.10	51	7.11E7	6	13723	30S ribosomal protein S11 OS=Candidatus Accumulibacter sp. BA-93 OX=1454004 GN=rpsK PE=3 SV=1
C7RJB8	176.10	51	7.11E7	6	13835	30S ribosomal protein S11 OS=Accumulibacter phosphatis (strain UW-1) OX=522306 GN=rpsK PE=3 SV=1
A0A011PPT5	172.84	59	2.23E8	6	11178	H-NS histone family protein OS=Candidatus Accumulibacter sp. BA-93 OX=1454004 GN=AW11_01537 PE=4 SV=1
A0A011RI37	172.78	73	7.03E7	6	11153	50S ribosomal protein L23 OS=Candidatus Accumulibacter sp. BA-93 OX=1454004 GN=rplW PE=3 SV=1
A0A011NPB6	172.64	27	8.01E7	6	29553	Uncharacterized protein OS=Candidatus Accumulibacter sp. BA-93 OX=1454004 GN=AW11_03791 PE=4 SV=1
A0A011P2V9	172.42	57	4.24E7	6	15934	Serogroup C1 OS=Candidatus Accumulibacter sp. BA-93 OX=1454004 GN=fimA_1 PE=4 SV=1
A0A011PVB5	171.44	24	2.27E6	6	36851	Delta-aminolevulinic acid dehydratase OS=Candidatus Accumulibacter sp. BA-93 OX=1454004 GN=hemB PE=3 SV=1
A0A011QLS4	169.98	14	3.58E6	6	113087	Bifunctional protein PutA OS=Candidatus Accumulibacter sp. BA-93 OX=1454004 GN=putA PE=3 SV=1
A0A011QLC2	168.46	10	5.12E7	6	79427	Bicarbonate transporter BicA OS=Candidatus Accumulibacter sp. BA-93 OX=1454004 GN=bicA PE=3 SV=1
A0A011RCE6	168.10	30	1.46E7	6	23473	Thiol:disulfide interchange protein OS=Candidatus Accumulibacter sp. BA-93 OX=1454004 GN=dsbA PE=3 SV=1
A0A011QJS7	167.10	19	2.36E6	6	83860	Outer membrane protein assembly factor BamA OS=Candidatus Accumulibacter sp. BA-93 OX=1454004 GN=bamA PE=3 SV=1
A0A011P4C6	165.79	17	1.45E6	6	69954	Glutamate synthase [NADPH] small chain OS=Candidatus Accumulibacter sp. BA-93 OX=1454004 GN=gltD PE=4 SV=1
A0A011QA04	160.41	20	2.47E7	6	42845	Patatin OS=Candidatus Accumulibacter sp. BA-93 OX=1454004 GN=AW11_03277 PE=4 SV=1
A0A011PKL2	160.21	8	2.35E7	6	111620	Uncharacterized protein OS=Candidatus Accumulibacter sp. BA-93 OX=1454004 GN=AW11_02320 PE=4 SV=1
A0A011PNY2	157.15	13	6.94E6	6	57328	Sensor protein ZraS OS=Candidatus Accumulibacter sp. BA-93 OX=1454004 GN=zraS_3 PE=4 SV=1
A0A011R7F5	156.76	31	1.23E8	6	21466	Ubiquinol-cytochrome c reductase iron-sulfur subunit OS=Candidatus Accumulibacter sp. BA-93 OX=1454004 GN=petA_2 PE=4 SV=1
A0A011PCI7	156.59	24	6.98E6	6	35071	Uncharacterized protein OS=Candidatus Accumulibacter sp. BA-93 OX=1454004 GN=AW11_03624 PE=4 SV=1
A0A011QCK9	156.35	33	2.27E7	6	29607	Cell division coordinator CpoB OS=Candidatus Accumulibacter sp. BA-93 OX=1454004 GN=cpoB PE=3 SV=1
A0A011QP68	155.97	21	1.8E6	6	52389	Magnesium transport protein CorA OS=Candidatus Accumulibacter sp. BA-93 OX=1454004 GN=corA PE=3 SV=1
A0A011P2R6	154.06	26	0.00E+00	6	37572	ADP-L-glycero-D-manno-heptose-6-epimerase OS=Candidatus Accumulibacter sp. BA-93 OX=1454004 GN=hldD PE=3 SV=1
A0A011RG10	154.00	57	3.04E7	6	16423	Uncharacterized protein OS=Candidatus Accumulibacter sp. BA-93 OX=1454004 GN=AW11_01062 PE=4 SV=1
A0A011PJB1	153.31	23	9.11E6	6	52186	Putative FAD-linked oxidoreductase OS=Candidatus Accumulibacter sp. BA-93 OX=1454004 GN=AW11_02475 PE=4 SV=1
A0A011P6I8	152.99	41	0.00E+00	6	34570	Ribose-phosphate pyrophosphokinase OS=Candidatus Accumulibacter sp. BA-93 OX=1454004 GN=prs PE=3 SV=1

A0A011QPS3	149.62	17	0.00E+00	6	42063	HDOD domain protein OS=Candidatus Accumulibacter sp. BA-93 OX=1454004 GN=AW11_00406 PE=4 SV=1
A0A011PNQ1	148.15	22	3.15E6	6	34725	Glutathione synthetase OS=Candidatus Accumulibacter sp. BA-93 OX=1454004 GN=gshB PE=3 SV=1
A0A011PNT9	145.33	20	6.23E7	6	29212	Outer membrane lipoprotein-sorting protein OS=Candidatus Accumulibacter sp. BA-93 OX=1454004 GN=AW11_01760 PE=4 SV=1
A0A011QJV6	144.60	24	3.11E7	6	33036	Cbb3-type cytochrome c oxidase subunit OS=Candidatus Accumulibacter sp. BA-93 OX=1454004 GN=ccoP PE=3 SV=1
A0A011PNL2	143.55	47	3.61E7	6	20280	Transcription termination/antitermination protein NusG OS=Candidatus Accumulibacter sp. BA-93 OX=1454004 GN=nusG PE=3 SV=1
A0A011PP06	142.19	47	9.38E6	6	17661	Peptidyl-prolyl cis-trans isomerase OS=Candidatus Accumulibacter sp. BA-93 OX=1454004 GN=ppiB PE=3 SV=1
A0A011Q7Y5	139.39	22	4.36E6	6	30020	Putative ABC transporter ATP-binding protein OS=Candidatus Accumulibacter sp. BA-93 OX=1454004 GN=AW11_03593 PE=4 SV=1
A0A011QK38	138.05	13	2.14E6	6	61670	Putative ABC transporter ATP-binding protein YjjK OS=Candidatus Accumulibacter sp. BA-93 OX=1454004 GN=yjjK PE=4 SV=1
A0A011PIM7	137.36	18	4.76E7	6	40102	UDP-2-acetamido-2-deoxy-3-oxo-D-glucuronate aminotransferase OS=Candidatus Accumulibacter sp. BA-93 OX=1454004 GN=wbpE_2 PE=3 SV=1
A0A369XQV1	137.14	11	0.00E+00	6	72338	Uncharacterized protein OS=Candidatus Accumulibacter phosphatis OX=327160 GN=DVS81_03885 PE=4 SV=1
A0A011QMI0	136.38	57	1.46E7	6	13425	Alkaline phosphatase synthesis transcriptional regulatory protein PhoP OS=Candidatus Accumulibacter sp. BA-92 OX=1454003 GN=phoP_2 PE=4 SV=1
A0A369XHG4	136.38	57	1.46E7	6	13439	Response regulator OS=Candidatus Accumulibacter phosphatis OX=327160 GN=DVS81_17230 PE=4 SV=1
A0A011PM79	136.20	14	2.35E6	6	48348	Phosphoglucosamine mutase OS=Candidatus Accumulibacter sp. BA-92 OX=1454003 GN=glmM PE=3 SV=1
A0A011QNA6	136.20	14	2.35E6	6	48361	Phosphoglucosamine mutase OS=Candidatus Accumulibacter sp. BA-93 OX=1454004 GN=glmM PE=3 SV=1
A0A011QP17	134.70	85	1.94E7	6	8242	Translation initiation factor IF-1 OS=Candidatus Accumulibacter sp. BA-93 OX=1454004 GN=infA PE=3 SV=1
A0A011QZB2	134.56	47	4.8E7	6	16945	Putative universal stress protein OS=Candidatus Accumulibacter sp. BA-93 OX=1454004 GN=AW11_03951 PE=4 SV=1
A0A011R801	131.79	12	0.00E+00	6	51703	Trk system potassium uptake protein TrkA OS=Candidatus Accumulibacter sp. BA-93 OX=1454004 GN=trkA PE=4 SV=1
A0A011PPN2	130.41	13	4.06E6	6	83955	Biodegradative arginine decarboxylase OS=Candidatus Accumulibacter sp. BA-93 OX=1454004 GN=adiA PE=4 SV=1
A0A011PIS1	129.42	23	1.91E8	6	21412	Uncharacterized protein OS=Candidatus Accumulibacter sp. BA-93 OX=1454004 GN=AW11_02549 PE=4 SV=1
A0A011Q460	129.41	13	9.06E6	6	97977	Aconitate hydratase OS=Candidatus Accumulibacter sp. BA-93 OX=1454004 GN=acn PE=3 SV=1
A0A011PNT0	127.90	19	3.23E6	6	34919	Putative acrylyl-CoA reductase AcuI OS=Candidatus Accumulibacter sp. BA-93 OX=1454004 GN=acuI_2 PE=4 SV=1

A0A011NT47	126.75	19	3.11E6	6	39426	Bifunctional polymyxin resistance protein ArnA OS=Candidatus Accumulibacter sp. BA-93 OX=1454004 GN=arnA_1 PE=4 SV=1
A0A011R573	123.95	15	1.11E7	6	51738	Periplasmic serine endoprotease DegP-like OS=Candidatus Accumulibacter sp. BA-93 OX=1454004 GN=mucD_4 PE=3 SV=1
A0A011QJV3	120.35	14	3.22E6	6	45951	Dihydroorotate dehydrogenase 1B OS=Candidatus Accumulibacter sp. BA-92 OX=1454003 GN=AW10_02535 PE=4 SV=1
A0A011PAK2	120.35	14	3.22E6	6	45865	Putative enoyl-[acyl-carrier-protein] reductase II OS=Candidatus Accumulibacter sp. BA-93 OX=1454004 GN=AW11_03776 PE=4 SV=1
A0A011QB83	119.32	12	0.00E+00	6	52782	Glutamate--cysteine ligase OS=Candidatus Accumulibacter sp. BA-93 OX=1454004 GN=AW11_03159 PE=4 SV=1
A0A011PN19	118.56	18	3.55E6	6	54047	Anthranilate synthase component 1 OS=Candidatus Accumulibacter sp. BA-93 OX=1454004 GN=trpE PE=3 SV=1
A0A011QMG8	113.47	43	1.97E7	6	15350	Acyl-CoA thioester hydrolase YbgC/YbaW family OS=Candidatus Accumulibacter sp. BA-93 OX=1454004 GN=AW11_00943 PE=4 SV=1
A0A011P8F4	109.80	10	0.00E+00	6	69365	Glutamine--tRNA ligase OS=Candidatus Accumulibacter sp. BA-93 OX=1454004 GN=glnS PE=3 SV=1
A0A011QI88	98.25	43	1.2E8	6	12651	50S ribosomal protein L7/L12 OS=Candidatus Accumulibacter sp. BA-93 OX=1454004 GN=rplL PE=3 SV=1
A0A011QNH6	89.33	12	1.07E7	6	66105	Lipoprotein NlpI OS=Candidatus Accumulibacter sp. BA-93 OX=1454004 GN=AW11_00446 PE=4 SV=1
A0A011QG34	372.24	55	1.14E8	5	67241	Phosphoenolpyruvate carboxykinase [GTP] OS=Candidatus Accumulibacter sp. BA-93 OX=1454004 GN=pckG PE=3 SV=1
A0A011P862	364.58	51	4.19E7	5	100603	Pyruvate dehydrogenase E1 component OS=Candidatus Accumulibacter sp. BA-93 OX=1454004 GN=aceE PE=4 SV=1
A0A011PUY8	359.70	67	2.13E8	5	50930	ATP synthase subunit beta OS=Candidatus Accumulibacter sp. BA-93 OX=1454004 GN=atpD_1 PE=3 SV=1
A0A011PWJ9	345.25	40	2.11E8	5	72391	Acetyl-/propionyl-coenzyme A carboxylase alpha chain OS=Candidatus Accumulibacter sp. BA-92 OX=1454003 GN=accA1_1 PE=4 SV=1
A0A011PN47	345.25	40	2.11E8	5	72391	Acetyl-/propionyl-coenzyme A carboxylase alpha chain OS=Candidatus Accumulibacter sp. BA-93 OX=1454004 GN=accA1_1 PE=4 SV=1
A0A011PTC9	332.16	58	1.15E8	5	54414	Poly(R)-hydroxyalkanoic acid synthase class III PhaC subunit OS=Candidatus Accumulibacter sp. BA-93 OX=1454004 GN=AW11_00513 PE=4 SV=1
A0A011QNL4	314.94	54	4.7E8	5	41079	Acetyl-CoA acetyltransferase OS=Candidatus Accumulibacter sp. BA-93 OX=1454004 GN=thlA PE=3 SV=1
A0A011Q5T0	312.00	41	5.98E7	5	48823	Citrate synthase OS=Candidatus Accumulibacter sp. BA-93 OX=1454004 GN=gltA_3 PE=3 SV=1
A0A011QI48	311.45	45	2.13E7	5	127458	Trehalose synthase/amylase TreS OS=Candidatus Accumulibacter sp. BA-93 OX=1454004 GN=treS PE=4 SV=1
A0A011RDN2	297.91	54	8.12E7	5	36417	Pyruvate synthase subunit PorB OS=Candidatus Accumulibacter sp. BA-93 OX=1454004 GN=porB PE=4 SV=1
A0A011P2B5	291.49	42	1.46E8	5	37526	Iron uptake protein A1 OS=Candidatus Accumulibacter sp. BA-93 OX=1454004 GN=futA1 PE=4 SV=1
A0A011PHK6	286.69	44	1.29E8	5	51341	Adenosylhomocysteinase OS=Candidatus Accumulibacter sp. BA-93 OX=1454004 GN=ahcY PE=3 SV=1

A0A011PVX8	285.12	55	8.22E8	5	40644	Poly-beta-hydroxybutyrate polymerase OS=Candidatus Accumulibacter sp. BA-92 OX=1454003 GN=phbC_2 PE=4 SV=1
A0A011R6S3	272.08	53	2.65E8	5	39040	Fructose-bisphosphate aldolase class 2 OS=Candidatus Accumulibacter sp. BA-93 OX=1454004 GN=fbaA PE=3 SV=1
A0A011QAW8	261.00	20	2.2E7	5	102291	Ribonuclease E OS=Candidatus Accumulibacter sp. BA-93 OX=1454004 GN=rne PE=3 SV=1
A0A011NUI9	254.48	38	1.13E7	5	58719	Glucose-6-phosphate isomerase OS=Candidatus Accumulibacter sp. BA-93 OX=1454004 GN=pgi PE=3 SV=1
A0A011QAU4	248.70	27	1.4E8	5	32407	C4-dicarboxylate-binding periplasmic protein OS=Candidatus Accumulibacter sp. BA-93 OX=1454004 GN=dctP_1 PE=4 SV=1
A0A011QNB7	240.81	46	8.85E7	5	38864	Alanine dehydrogenase OS=Candidatus Accumulibacter sp. BA-93 OX=1454004 GN=ald2 PE=3 SV=1
A0A011QQ29	238.35	53	9.45E7	5	31465	ATP synthase gamma chain OS=Candidatus Accumulibacter sp. BA-93 OX=1454004 GN=atpG PE=3 SV=1
A0A011Q801	234.43	62	2.52E8	5	19244	Phasin family protein OS=Candidatus Accumulibacter sp. BA-93 OX=1454004 GN=AW11_03613 PE=4 SV=1
A0A011P9E7	230.10	44	9.55E7	5	30948	4-hydroxy-tetrahydrodipicolinate synthase OS=Candidatus Accumulibacter sp. BA-93 OX=1454004 GN=dapA PE=3 SV=1
A0A011Q858	223.08	31	7.44E6	5	48071	Sulfate adenylyltransferase subunit 1 OS=Candidatus Accumulibacter sp. BA-93 OX=1454004 GN=cysN PE=3 SV=1
A0A011P0F6	219.55	27	5.9E7	5	48747	Chaperone SurA OS=Candidatus Accumulibacter sp. BA-93 OX=1454004 GN=surA PE=3 SV=1
A0A011RGV3	216.60	38	1.78E7	5	59510	Malate synthase OS=Candidatus Accumulibacter sp. BA-93 OX=1454004 GN=aceB PE=3 SV=1
A0A011PMZ5	210.72	67	2.86E8	5	17905	Protein-export protein SecB OS=Candidatus Accumulibacter sp. BA-93 OX=1454004 GN=secB PE=3 SV=1
A0A011PAE1	209.67	33	1.12E6	5	59152	Electron transfer flavoprotein-ubiquinone oxidoreductase OS=Candidatus Accumulibacter sp. BA-93 OX=1454004 GN=AW11_03818 PE=4 SV=1
A0A011PB12	209.32	42	3.88E6	5	47718	UDP-glucose 6-dehydrogenase OS=Candidatus Accumulibacter sp. BA-93 OX=1454004 GN=ywqF PE=3 SV=1
A0A011Q870	201.87	15	6.47E6	5	105436	Glycine dehydrogenase (decarboxylating) OS=Candidatus Accumulibacter sp. BA-93 OX=1454004 GN=gcvP PE=3 SV=1
A0A011P8B8	201.38	52	1.28E8	5	19155	ATP synthase subunit delta OS=Candidatus Accumulibacter sp. BA-93 OX=1454004 GN=atpH PE=3 SV=1
A0A011P365	197.47	51	8.04E6	5	26037	1-(5-phosphoribosyl)-5-[(5-phosphoribosylamino)methylideneamino] imidazole-4-carboxamide isomerase OS=Candidatus Accumulibacter sp. BA-93 OX=1454004 GN=hisA PE=3 SV=1
A0A011QEV2	195.23	29	8.42E7	5	30792	Phosphate-binding protein PstS 1 OS=Candidatus Accumulibacter sp. BA-93 OX=1454004 GN=pstS1_1 PE=4 SV=1
A0A011QJM3	194.41	47	1.96E8	5	14489	Universal stress protein F OS=Candidatus Accumulibacter sp. BA-93 OX=1454004 GN=uspF PE=4 SV=1
A0A011NU58	190.02	49	2.8E8	5	8786	Acyl carrier protein OS=Candidatus Accumulibacter sp. BA-93 OX=1454004 GN=acpP PE=3 SV=1
A0A011PYZ3	190.02	49	2.8E8	5	8786	Acyl carrier protein OS=Candidatus Accumulibacter sp. BA-92 OX=1454003 GN=acpP PE=3 SV=1
A0A011RBD3	187.09	43	9.17E7	5	17516	Putative membrane protein OS=Candidatus Accumulibacter sp. BA-93 OX=1454004 GN=AW11_02094 PE=4 SV=1

A0A369XMY0	186.91	22	4.56E7	5	82229	DUF3604 domain-containing protein OS=Candidatus Accumulibacter phosphatis OX=327160 GN=DVS81_05670 PE=4 SV=1
A0A011RH85	181.69	32	2.62E8	5	23697	50S ribosomal protein L25 OS=Candidatus Accumulibacter sp. BA-93 OX=1454004 GN=rplY PE=3 SV=1
A0A011P678	180.08	41	5.1E7	5	24245	Uncharacterized protein OS=Candidatus Accumulibacter sp. BA-93 OX=1454004 GN=AW11_00818 PE=4 SV=1
A0A011Q8B8	179.70	13	0.00E+00	5	82460	Putative tyrosine-protein kinase in cps region OS=Candidatus Accumulibacter sp. BA-93 OX=1454004 GN=AW11_03533 PE=4 SV=1
A0A011QDR4	177.48	42	0.00E+00	5	30884	Orotidine 5'-phosphate decarboxylase OS=Candidatus Accumulibacter sp. BA-93 OX=1454004 GN=pyrF PE=3 SV=1
A0A011R3K7	176.34	29	3.53E6	5	39329	Ethanolamine utilization protein EutJ OS=Candidatus Accumulibacter sp. BA-93 OX=1454004 GN=AW11_03349 PE=4 SV=1
A0A011N4N8	172.94	18	3.63E7	5	33297	Uncharacterized protein OS=Candidatus Accumulibacter sp. BA-92 OX=1454003 GN=AW10_03672 PE=4 SV=1
A0A011R556	169.25	25	1.11E7	5	43434	Solvent efflux pump periplasmic linker SrpA OS=Candidatus Accumulibacter sp. BA-93 OX=1454004 GN=srpA PE=3 SV=1
A0A011QAZ4	167.38	37	8.64E7	5	20878	Elongation factor P OS=Candidatus Accumulibacter sp. BA-93 OX=1454004 GN=efp PE=3 SV=1
A0A369XLL2	167.38	37	8.64E7	5	20859	Elongation factor P OS=Candidatus Accumulibacter phosphatis OX=327160 GN=efp PE=4 SV=1
A0A011QJS0	164.94	27	2.06E7	5	20789	Ribosome-recycling factor OS=Candidatus Accumulibacter sp. BA-93 OX=1454004 GN=frr PE=3 SV=1
A0A011P225	164.67	29	6.73E7	5	17267	50S ribosomal protein L10 OS=Candidatus Accumulibacter sp. BA-93 OX=1454004 GN=rplJ PE=3 SV=1
A0A011QGJ7	162.81	23	1.12E7	5	55947	Bifunctional purine biosynthesis protein PurH OS=Candidatus Accumulibacter sp. BA-93 OX=1454004 GN=purH PE=3 SV=1
A0A011NUL2	162.09	46	5.04E7	5	15870	Universal stress protein UspE OS=Candidatus Accumulibacter sp. BA-93 OX=1454004 GN=AW11_03187 PE=4 SV=1
A0A011R1Z5	162.04	29	7.86E7	5	25617	Thiol:disulfide interchange protein OS=Candidatus Accumulibacter sp. BA-93 OX=1454004 GN=dsbC_2 PE=3 SV=1
A0A011QP23	156.69	50	5.52E6	5	12781	50S ribosomal protein L18 OS=Candidatus Accumulibacter sp. BA-93 OX=1454004 GN=rplR PE=3 SV=1
A0A011R5B6	156.36	21	7.93E6	5	42831	Putrescine-binding periplasmic protein OS=Candidatus Accumulibacter sp. BA-93 OX=1454004 GN=potF PE=3 SV=1
A0A011QAL6	155.74	37	7.88E6	5	25395	3-oxoacyl-[acyl-carrier-protein] reductase FabG OS=Candidatus Accumulibacter sp. BA-93 OX=1454004 GN=fabG_4 PE=4 SV=1
A0A080LTV2	153.76	10	2.81E6	5	67656	Tyrosine phosphorylated protein A OS=Candidatus Accumulibacter sp. BA-91 OX=1454002 GN=typA PE=4 SV=1
A0A011QPC0	153.11	13	1.02E7	5	51482	Uncharacterized protein OS=Candidatus Accumulibacter sp. BA-93 OX=1454004 GN=AW11_00191 PE=4 SV=1
A0A011QJ50	153.09	33	7.81E7	5	14889	50S ribosomal protein L11 OS=Candidatus Accumulibacter sp. BA-93 OX=1454004 GN=rplK PE=3 SV=1
A0A011QK74	151.99	13	3.22E6	5	66043	Heat shock protein 70 OS=Candidatus Accumulibacter sp. BA-93 OX=1454004 GN=dnaK_1 PE=3 SV=1
A0A011RGS2	151.76	49	1.61E7	5	13854	50S ribosomal protein L19 OS=Candidatus Accumulibacter sp. BA-93 OX=1454004 GN=rplS PE=3 SV=1
A0A369XY87	151.76	49	1.61E7	5	13854	50S ribosomal protein L19 OS=Candidatus Accumulibacter phosphatis OX=327160 GN=DVS81_00670 PE=4 SV=1

A0A011QB49	151.10	21	3.29E8	5	24840	Uncharacterized protein OS=Candidatus Accumulibacter sp. BA-93 OX=1454004 GN=AW11_03124 PE=4 SV=1
A0A011QGK9	147.52	12	2.19E6	5	89098	LPS-assembly protein LptD OS=Candidatus Accumulibacter sp. BA-93 OX=1454004 GN=lptD PE=3 SV=1
A0A011QK56	147.32	37	0.00E+00	5	21444	dCTP deaminase OS=Candidatus Accumulibacter sp. BA-93 OX=1454004 GN=dcd PE=3 SV=1
A0A011R2F5	145.50	42	1.59E7	5	12432	Putative pterin-4-alpha-carbinolamine dehydratase OS=Candidatus Accumulibacter sp. BA-93 OX=1454004 GN=AW11_03502 PE=3 SV=1
A0A080LRH0	145.21	13	3.19E8	5	55880	Glycerol kinase OS=Candidatus Accumulibacter sp. BA-91 OX=1454002 GN=glpK PE=3 SV=1
A0A011PLF1	145.14	36	1.51E7	5	22434	Polysaccharide export protein Wza OS=Candidatus Accumulibacter sp. BA-93 OX=1454004 GN=AW11_02213 PE=4 SV=1
A0A011QMU1	142.90	21	2.5E6	5	38909	Poly(R)-hydroxyalkanoic acid synthase class III PhaE subunit OS=Candidatus Accumulibacter sp. BA-93 OX=1454004 GN=AW11_00499 PE=4 SV=1
A0A369XIF3	142.00	19	6.24E6	5	38640	Glycerate kinase OS=Candidatus Accumulibacter phosphatis OX=327160 GN=DVS81_15470 PE=4 SV=1
A0A011QMW5	141.71	17	0.00E+00	5	38757	CRISPR system Cascade subunit CasC OS=Candidatus Accumulibacter sp. BA-93 OX=1454004 GN=casC PE=4 SV=1
A0A011QPI4	137.64	35	8.24E7	5	15176	ATP synthase epsilon chain OS=Candidatus Accumulibacter sp. BA-93 OX=1454004 GN=atpC PE=3 SV=1
A0A011QAX8	136.00	16	2.07E7	5	33329	3-oxoacyl-[acyl-carrier-protein] synthase 3 OS=Candidatus Accumulibacter sp. BA-93 OX=1454004 GN=fabH PE=3 SV=1
A0A369XMP0	136.00	16	2.07E7	5	33387	Ketoacyl-ACP synthase III OS=Candidatus Accumulibacter phosphatis OX=327160 GN=DVS81_15220 PE=4 SV=1
A0A011RJB7	135.84	20	2.00E+07	5	33615	Adenosine kinase OS=Candidatus Accumulibacter sp. BA-93 OX=1454004 GN=adoK PE=4 SV=1
A0A011QEU3	135.83	18	3.8E6	5	37386	Putative FAD-linked oxidoreductase OS=Candidatus Accumulibacter sp. BA-93 OX=1454004 GN=AW11_02474 PE=4 SV=1
A0A369XQ92	134.79	50	2.19E7	5	11324	Integration host factor subunit alpha OS=Candidatus Accumulibacter phosphatis OX=327160 GN=DVS81_10555 PE=4 SV=1
A0A011PLA8	134.79	50	2.19E7	5	11294	Integration host factor subunit alpha OS=Candidatus Accumulibacter sp. BA-92 OX=1454003 GN=ihfA PE=3 SV=1
A0A011QFE2	134.79	50	2.19E7	5	11294	Integration host factor subunit alpha OS=Candidatus Accumulibacter sp. BA-93 OX=1454004 GN=ihfA PE=3 SV=1
A0A011RJ67	131.72	17	1.38E6	5	47386	Magnesium and cobalt efflux protein CorC OS=Candidatus Accumulibacter sp. BA-93 OX=1454004 GN=corC_1 PE=4 SV=1
A0A011PTI0	131.25	24	1.48E8	5	23065	Uncharacterized protein OS=Candidatus Accumulibacter sp. BA-93 OX=1454004 GN=AW11_00569 PE=4 SV=1
A0A011Q5F5	128.28	64	2.45E7	5	6545	UPF0434 protein AW11_03871 OS=Candidatus Accumulibacter sp. BA-93 OX=1454004 GN=AW11_03871 PE=3 SV=1
A0A011QPW2	125.69	46	2.71E7	5	11734	Acetolactate synthase isozyme 1 small subunit OS=Candidatus Accumulibacter sp. BA-93 OX=1454004 GN=ilvN PE=4 SV=1
A0A011NT11	125.68	16	1.77E6	5	51415	Glutathione reductase OS=Candidatus Accumulibacter sp. BA-93 OX=1454004 GN=garB PE=3 SV=1
A0A011PCD9	124.53	15	5.94E6	5	45105	UDP-N-acetylglucosamine 1-carboxyvinyltransferase OS=Candidatus Accumulibacter sp. BA-93 OX=1454004 GN=murA PE=3 SV=1

A0A011PLT1	124.19	18	1.1E6	5	45265	Phosphoribosylamine--glycine ligase OS=Candidatus Accumulibacter sp. BA-93 OX=1454004 GN=purD PE=3 SV=1
A0A011Q9C6	123.23	10	0.00E+00	5	66273	Glutamine--fructose-6-phosphate aminotransferase [isomerizing] OS=Candidatus Accumulibacter sp. BA-93 OX=1454004 GN=glmS PE=3 SV=1
A0A011P8E9	121.67	40	1.71E6	5	17015	Uncharacterized protein OS=Candidatus Accumulibacter sp. BA-93 OX=1454004 GN=AW11_00053 PE=4 SV=1
A0A011PC91	120.91	24	6.43E6	5	26777	Lipopolysaccharide export system ATP-binding protein LptB OS=Candidatus Accumulibacter sp. BA-93 OX=1454004 GN=lptB_2 PE=4 SV=1
A0A011QJR5	119.49	26	2.00E+06	5	29577	Methionine aminopeptidase OS=Candidatus Accumulibacter sp. BA-93 OX=1454004 GN=map PE=3 SV=1
A0A011PQ25	119.34	18	1.42E7	5	20215	Flagellar protein FliL OS=Candidatus Accumulibacter sp. BA-93 OX=1454004 GN=AW11_01392 PE=3 SV=1
A0A011P002	119.28	28	7.2E6	5	21550	Uncharacterized protein OS=Candidatus Accumulibacter sp. BA-93 OX=1454004 GN=AW11_02179 PE=4 SV=1
A0A011QMM7	119.08	29	4.29E7	5	20911	Uncharacterized protein OS=Candidatus Accumulibacter sp. BA-93 OX=1454004 GN=AW11_01003 PE=4 SV=1
A0A011RDP4	117.94	9	4.33E6	5	73383	Methylmalonyl-CoA mutase OS=Candidatus Accumulibacter sp. BA-93 OX=1454004 GN=scpA_1 PE=4 SV=1
A0A011PPB0	110.60	27	9.00E+07	5	19677	Cationic 19 kDa outer membrane protein OS=Candidatus Accumulibacter sp. BA-93 OX=1454004 GN=skp PE=3 SV=1
A0A011P3P6	110.50	34	0.00E+00	5	18296	UPF0234 protein AW11_01435 OS=Candidatus Accumulibacter sp. BA-93 OX=1454004 GN=AW11_01435 PE=3 SV=1
A0A011QJI1	109.51	13	0.00E+00	5	51372	Cysteine--tRNA ligase OS=Candidatus Accumulibacter sp. BA-93 OX=1454004 GN=cysS PE=3 SV=1
A0A011RC89	109.24	29	3.2E7	5	21800	CBS domain protein OS=Candidatus Accumulibacter sp. BA-93 OX=1454004 GN=AW11_01942 PE=4 SV=1
A0A080MJJ4	104.48	7	3.34E7	5	61587	Aerobic glycerol-3-phosphate dehydrogenase OS=Candidatus Accumulibacter sp. SK-02 OX=1453999 GN=glpD PE=4 SV=1
A0A011PT06	101.92	36	5.25E6	5	17441	NADH-quinone oxidoreductase subunit B OS=Candidatus Accumulibacter sp. BA-93 OX=1454004 GN=nuoB PE=3 SV=1
A0A011QET8	101.60	12	1.44E6	5	54102	ATP-dependent RNA helicase RhlE OS=Candidatus Accumulibacter sp. BA-93 OX=1454004 GN=rhlE_2 PE=3 SV=1
A0A011PNW5	101.60	12	1.44E6	5	54736	ATP-dependent RNA helicase RhlE OS=Candidatus Accumulibacter sp. BA-92 OX=1454003 GN=rhlE_2 PE=3 SV=1
A0A011RBH6	100.37	15	2.43E6	5	60827	Putative ABC transporter ATP-binding protein YheS OS=Candidatus Accumulibacter sp. BA-93 OX=1454004 GN=yheS_2 PE=4 SV=1
A0A369XNH5	99.79	10	2.04E6	5	64216	Nitrite/sulfite reductase OS=Candidatus Accumulibacter phosphatis OX=327160 GN=DVS81_08845 PE=4 SV=1
A0A011NBJ1	99.79	10	2.04E6	5	64067	Sulfite reductase [ferredoxin] OS=Candidatus Accumulibacter sp. BA-92 OX=1454003 GN=sir PE=4 SV=1
A0A011PG10	93.33	29	9.82E6	5	16327	Transcriptional regulator MraZ OS=Candidatus Accumulibacter sp. BA-93 OX=1454004 GN=mraZ PE=3 SV=1

A0A011PQ41	89.23	18	3.85E7	5	32721	Uncharacterized protein OS=Candidatus Accumulibacter sp. BA-92 OX=1454003 GN=AW10_02528 PE=4 SV=1
A0A011QZY8	88.87	11	0.00E+00	5	73192	Acetyl-/propionyl-coenzyme A carboxylase alpha chain OS=Candidatus Accumulibacter sp. BA-93 OX=1454004 GN=accA1_2 PE=4 SV=1
A0A011PES6	86.10	6	9.06E5	5	96559	Leucine--tRNA ligase OS=Candidatus Accumulibacter sp. BA-93 OX=1454004 GN=leuS PE=3 SV=1
A0A011Q9W9	81.16	18	0.00E+00	5	31364	FecR protein OS=Candidatus Accumulibacter sp. BA-93 OX=1454004 GN=AW11_03251 PE=4 SV=1
A0A011QNB0	400.29	52	4.21E8	4	71227	Acetyl-coenzyme A synthetase OS=Candidatus Accumulibacter sp. BA-93 OX=1454004 GN=acsA_1 PE=3 SV=1
A0A011P1K0	368.38	57	7.32E7	4	78353	Methylmalonyl-CoA mutase OS=Candidatus Accumulibacter sp. BA-93 OX=1454004 GN=scpA_3 PE=4 SV=1
A0A011P2R1	343.26	46	1.96E7	4	61580	30S ribosomal protein S1 OS=Candidatus Accumulibacter sp. BA-93 OX=1454004 GN=rpsA PE=3 SV=1
A0A011QPR9	322.92	70	8.22E8	4	42779	Leucine- isoleucine- valine- threonine- and alanine-binding protein OS=Candidatus Accumulibacter sp. BA-93 OX=1454004 GN=braC_1 PE=4 SV=1
A0A011R0G4	319.14	49	1.69E8	4	64626	Succinate dehydrogenase flavoprotein subunit OS=Candidatus Accumulibacter sp. BA-93 OX=1454004 GN=sdhA PE=3 SV=1
A0A011R3J6	306.05	41	2.33E8	4	55141	NAD(P) transhydrogenase subunit alpha OS=Candidatus Accumulibacter sp. BA-93 OX=1454004 GN=pntA PE=3 SV=1
A0A011QMG3	261.01	52	2.27E8	4	26777	Electron transfer flavoprotein small subunit OS=Candidatus Accumulibacter sp. BA-93 OX=1454004 GN=etfB PE=4 SV=1
A0A011QII5	244.36	17	5.45E8	4	38273	Outer membrane porin protein 32 OS=Candidatus Accumulibacter sp. BA-93 OX=1454004 GN=AW11_01958 PE=4 SV=1
A0A011Q9K5	242.89	23	5.1E7	4	83902	1 4-alpha-glucan branching enzyme GlgB OS=Candidatus Accumulibacter sp. BA-93 OX=1454004 GN=glgB_2 PE=3 SV=1
A0A011N5Q1	230.56	37	2.21E6	4	59444	Malate synthase OS=Candidatus Accumulibacter sp. BA-92 OX=1454003 GN=aceB PE=3 SV=1
A0A011P267	225.44	39	3.46E7	4	36447	Rod shape-determining protein MreB OS=Candidatus Accumulibacter sp. BA-93 OX=1454004 GN=mreB PE=4 SV=1
A0A011QFF6	225.44	39	3.46E7	4	36481	Rod shape-determining protein MreB OS=Candidatus Accumulibacter sp. BA-92 OX=1454003 GN=mreB PE=4 SV=1
A0A011PQ57	218.13	31	1.54E8	4	43745	Dihydrolipoyllysine-residue succinyltransferase component of 2-oxoglutarate dehydrogenase complex OS=Candidatus Accumulibacter sp. BA-92 OX=1454003 GN=sucB PE=3 SV=1
A0A011P2Z7	213.48	51	1.00E+07	4	38036	Malyl-CoA lyase OS=Candidatus Accumulibacter sp. BA-93 OX=1454004 GN=mcl1_1 PE=3 SV=1
A0A011QDF2	212.37	73	1.51E9	4	10511	10 kDa chaperonin OS=Candidatus Accumulibacter sp. BA-93 OX=1454004 GN=groS PE=3 SV=1
A0A011Q430	212.27	46	1.59E6	4	32728	Cysteine synthase OS=Candidatus Accumulibacter sp. BA-93 OX=1454004 GN=cysK1 PE=3 SV=1
A0A011QPC6	211.22	35	1.77E8	4	14838	Surface antigen OS=Candidatus Accumulibacter sp. BA-93 OX=1454004 GN=AW11_00196 PE=4 SV=1
A0A011PJ88	204.25	33	2.96E7	4	28811	Sulfate starvation-induced protein 7 OS=Candidatus Accumulibacter sp. BA-93 OX=1454004 GN=fliY_2 PE=3 SV=1
A0A011NSQ6	195.26	25	6.03E6	4	82075	1 4-alpha-glucan branching enzyme GlgB OS=Candidatus Accumulibacter sp. BA-93 OX=1454004 GN=glgB_3 PE=3 SV=1
A0A011NMQ4	184.82	19	1.15E7	4	34328	Sirohydrochlorin cobaltochelatase OS=Candidatus Accumulibacter sp. BA-93 OX=1454004 GN=cbiX_2 PE=4 SV=1

A0A011Q4F6	182.90	27	0.00E+00	4	34274	Cobalt-precorrin-3B C(17)-methyltransferase OS=Candidatus Accumulibacter sp. BA-93 OX=1454004 GN=cbiH PE=4 SV=1
A0A011QL62	173.82	29	5.29E6	4	33449	N-acetylmuramoyl-L-alanine amidase AmiD OS=Candidatus Accumulibacter sp. BA-93 OX=1454004 GN=amiD_1 PE=4 SV=1
A0A011QP31	170.21	20	9.51E6	4	34568	Uncharacterized protein OS=Candidatus Accumulibacter sp. BA-93 OX=1454004 GN=AW11_00388 PE=4 SV=1
A0A011QNG2	168.91	61	2.14E8	4	14152	30S ribosomal protein S8 OS=Candidatus Accumulibacter sp. BA-93 OX=1454004 GN=rpsH PE=3 SV=1
A0A369XTU3	168.91	61	2.14E8	4	14053	30S ribosomal protein S8 OS=Candidatus Accumulibacter phosphatis OX=327160 GN=DVS81_09250 PE=4 SV=1
A0A011PMP4	168.91	58	2.14E8	4	14966	30S ribosomal protein S8 OS=Candidatus Accumulibacter sp. BA-92 OX=1454003 GN=rpsH PE=3 SV=1
A0A011NS51	168.68	21	1.15E7	4	50322	Phosphate acetyltransferase OS=Candidatus Accumulibacter sp. BA-93 OX=1454004 GN=pta_2 PE=4 SV=1
A0A011NMU6	167.59	29	1.2E7	4	13595	Putative lactoylglutathione lyase OS=Candidatus Accumulibacter sp. BA-93 OX=1454004 GN=AW11_03992 PE=4 SV=1
A0A011QIS7	165.08	28	1.06E7	4	33607	Protein HflC OS=Candidatus Accumulibacter sp. BA-93 OX=1454004 GN=hflC PE=3 SV=1
A0A1A8XX18	162.91	25	3.04E7	4	22178	Uncharacterized protein OS=Candidatus Accumulibacter aalborgensis OX=1860102 GN=ACCAA_580044 PE=4 SV=1
A0A011RJ14	162.59	54	2.13E8	4	16318	Uncharacterized protein OS=Candidatus Accumulibacter sp. BA-93 OX=1454004 GN=AW11_00218 PE=4 SV=1
A0A011QJM7	162.07	31	5.95E6	4	35600	Biotin synthase OS=Candidatus Accumulibacter sp. BA-92 OX=1454003 GN=bioB PE=3 SV=1
A0A369XVZ2	162.07	31	5.95E6	4	35586	Biotin synthase BioB OS=Candidatus Accumulibacter phosphatis OX=327160 GN=bioB PE=4 SV=1
A0A011QPC3	161.79	35	1.62E7	4	17705	C-lysozyme inhibitor OS=Candidatus Accumulibacter sp. BA-93 OX=1454004 GN=AW11_00266 PE=4 SV=1
A0A011MI58	157.61	14	0.00E+00	4	33694	UDP-glucose 4-epimerase OS=Candidatus Accumulibacter sp. SK-12 OX=1454001 GN=AW08_00106 PE=4 SV=1
A0A011P0E0	157.27	15	8.09E6	4	52246	Putative uroporphyrinogen-III C-methyltransferase OS=Candidatus Accumulibacter sp. BA-93 OX=1454004 GN=hemX PE=4 SV=1
A0A084XU28	154.65	32	6.58E7	4	25881	3-oxoacyl-[acyl-carrier-protein] reductase FabG OS=Candidatus Accumulibacter sp. SK-01 OX=1457154 GN=fabG_6 PE=3 SV=1
C7RVI7	154.65	32	6.58E7	4	25868	Short-chain dehydrogenase/reductase SDR OS=Accumulibacter phosphatis (strain UW-1) OX=522306 GN=CAP2UW1_3190 PE=3 SV=1
A0A011PUK1	154.28	13	2.28E6	4	54893	Ornithine/acetylornithine aminotransferase OS=Candidatus Accumulibacter sp. BA-93 OX=1454004 GN=AW11_00437 PE=4 SV=1
A0A011P9S3	154.28	30	2.28E6	4	24364	Type IV secretory pathway VirB4 component OS=Candidatus Accumulibacter sp. BA-92 OX=1454003 GN=AW10_04236 PE=4 SV=1
A0A011QMP5	154.13	36	1.19E8	4	12679	4-carboxymuconolactone decarboxylase OS=Candidatus Accumulibacter sp. BA-93 OX=1454004 GN=AW11_00459 PE=4 SV=1
A0A011QKM1	153.65	16	0.00E+00	4	57443	GMP synthase [glutamine-hydrolyzing] OS=Candidatus Accumulibacter sp. BA-93 OX=1454004 GN=guaA PE=3 SV=1

A0A011R1T7	151.61	37	5.67E7	4	21363	Uncharacterized protein OS=Candidatus Accumulibacter sp. BA-93 OX=1454004 GN=AW11_03698 PE=4 SV=1
A0A369XI09	149.57	16	1.66E7	4	38700	Iron-sulfur cluster carrier protein ApbC OS=Candidatus Accumulibacter phosphatis OX=327160 GN=DVS81_18675 PE=4 SV=1
A0A011RDU2	149.57	16	1.66E7	4	38736	Iron-sulfur cluster carrier protein OS=Candidatus Accumulibacter sp. BA-93 OX=1454004 GN=ylxH_2 PE=3 SV=1
A0A011P6B0	148.11	39	0.00E+00	4	17130	NADH-quinone oxidoreductase subunit 2 OS=Candidatus Accumulibacter sp. BA-93 OX=1454004 GN=nqo2 PE=4 SV=1
A0A011R1I3	144.34	18	5.96E6	4	44202	Cell division protein ZipA OS=Candidatus Accumulibacter sp. BA-93 OX=1454004 GN=AW11_03722 PE=3 SV=1
A0A011PT72	143.39	22	9.89E6	4	22195	Uncharacterized protein OS=Candidatus Accumulibacter sp. BA-93 OX=1454004 GN=AW11_00458 PE=4 SV=1
A0A011QKA7	143.22	39	0.00E+00	4	15850	Molybdopterin biosynthesis protein MoeB OS=Candidatus Accumulibacter sp. BA-93 OX=1454004 GN=AW11_01297 PE=4 SV=1
A0A011QLN4	142.66	23	1.35E7	4	27511	PEP-CTERM motif protein OS=Candidatus Accumulibacter sp. BA-93 OX=1454004 GN=AW11_01196 PE=4 SV=1
A0A011P848	141.45	28	9.65E6	4	25027	Uncharacterized protein OS=Candidatus Accumulibacter sp. BA-93 OX=1454004 GN=AW11_00182 PE=4 SV=1
A0A011NX08	140.23	24	2.03E7	4	29191	Uncharacterized protein OS=Candidatus Accumulibacter sp. BA-93 OX=1454004 GN=AW11_02668 PE=4 SV=1
A0A011QHF1	135.88	9	6.07E6	4	67613	Malto-oligosyltrehalose trehalohydrolase OS=Candidatus Accumulibacter sp. BA-93 OX=1454004 GN=treZ PE=3 SV=1
A0A011NNT7	135.63	20	1.7E7	4	28387	Putative enoyl-CoA hydratase echA8 OS=Candidatus Accumulibacter sp. BA-93 OX=1454004 GN=echA8_6 PE=4 SV=1
A0A011Q813	135.50	9	2.85E6	4	54882	Cytochrome c mono-and diheme variant OS=Candidatus Accumulibacter sp. BA-93 OX=1454004 GN=AW11_03628 PE=4 SV=1
A0A011QBS4	135.12	13	0.00E+00	4	52725	UDP-N-acetylmuramoyl-L-alanyl-D-glutamate--2 6-diaminopimelate ligase OS=Candidatus Accumulibacter sp. BA-93 OX=1454004 GN=murE PE=3 SV=1
A0A011QB42	135.05	16	0.00E+00	4	49561	Pyruvate kinase OS=Candidatus Accumulibacter sp. BA-93 OX=1454004 GN=pyk_2 PE=3 SV=1
A0A011QD58	134.97	23	5.46E6	4	18797	Periplasmic repressor CpxP OS=Candidatus Accumulibacter sp. BA-93 OX=1454004 GN=AW11_02767 PE=4 SV=1
A0A351BGX1	134.63	12	1.13E7	4	37850	HAD family hydrolase OS=Candidatus Accumulibacter sp. OX=2053492 GN=DCY47_08585 PE=4 SV=1
A0A011Q434	134.07	10	4.53E5	4	63129	Proline--tRNA ligase OS=Candidatus Accumulibacter sp. BA-93 OX=1454004 GN=proS PE=3 SV=1
A0A011Q9V7	133.75	14	0.00E+00	4	70192	Isocitrate dehydrogenase kinase/phosphatase OS=Candidatus Accumulibacter sp. BA-93 OX=1454004 GN=aceK PE=3 SV=1
A0A011P480	132.87	42	0.00E+00	4	22452	LemA family protein OS=Candidatus Accumulibacter sp. BA-93 OX=1454004 GN=AW11_01248 PE=4 SV=1
A0A011QDV7	132.86	28	1.09E7	4	24148	Uncharacterized protein OS=Candidatus Accumulibacter sp. BA-93 OX=1454004 GN=AW11_02611 PE=4 SV=1
A0A011R1T1	132.85	29	1.56E7	4	15925	Camphor resistance protein CrcB OS=Candidatus Accumulibacter sp. BA-93 OX=1454004 GN=AW11_03688 PE=4 SV=1

A0A011P7S7	131.23	6	1.77E7	4	92868	Neu5Ac permease OS=Candidatus Accumulibacter sp. BA-93 OX=1454004 GN=siaT_1 PE=4 SV=1
A0A011NZH8	130.28	15	0.00E+00	4	38342	ATP-dependent zinc metalloprotease FtsH OS=Candidatus Accumulibacter sp. BA-93 OX=1454004 GN=ftsH_2 PE=3 SV=1
A0A011RG04	130.18	22	9.37E7	4	20687	Cytochrome c oxidase subunit 2 OS=Candidatus Accumulibacter sp. BA-93 OX=1454004 GN=cbaB PE=4 SV=1
A0A011QLV8	130.18	22	9.37E7	4	20673	Cytochrome c oxidase subunit 2 OS=Candidatus Accumulibacter sp. BA-92 OX=1454003 GN=cbaB PE=4 SV=1
A0A080MC82	130.14	70	3.09E9	4	7174	Cold shock-like protein CspD OS=Candidatus Accumulibacter sp. SK-02 OX=1453999 GN=cspD PE=4 SV=1
A0A084Y5Q1	130.14	70	3.09E9	4	7174	Cold shock-like protein CspD OS=Candidatus Accumulibacter sp. SK-01 OX=1457154 GN=cspD PE=4 SV=1
A0A369XVV1	130.14	70	3.09E9	4	7158	Cold-shock protein OS=Candidatus Accumulibacter phosphatis OX=327160 GN=DVS81_00540 PE=4 SV=1
A0A080M649	130.14	70	3.09E9	4	7174	Cold shock-like protein CspD OS=Candidatus Accumulibacter sp. BA-91 OX=1454002 GN=cspD PE=4 SV=1
C7RNB2	130.14	70	3.09E9	4	7174	Cold-shock DNA-binding domain protein OS=Accumulibacter phosphatis (strain UW-1) OX=522306 GN=CAP2UW1_4056 PE=4 SV=1
A0A1Q3VLN5	130.14	70	3.09E9	4	7215	Cold-shock protein OS=Candidatus Accumulibacter sp. 66-26 OX=1895689 GN=BGO63_01820 PE=4 SV=1
A0A011QN46	130.14	70	3.09E9	4	7158	Cold shock-like protein CspD OS=Candidatus Accumulibacter sp. BA-93 OX=1454004 GN=cspD PE=4 SV=1
A0A1A8XNK1	130.14	70	3.09E9	4	7174	DNA-binding transcriptional repressor OS=Candidatus Accumulibacter aalborgensis OX=1860102 GN=cspE PE=4 SV=1
A0A011N5N9	130.14	70	3.09E9	4	7158	Cold shock-like protein CspD OS=Candidatus Accumulibacter sp. BA-92 OX=1454003 GN=cspD PE=4 SV=1
A0A011QB36	130.08	28	3.27E7	4	20305	Methyl-accepting chemotaxis protein 4 OS=Candidatus Accumulibacter sp. BA-93 OX=1454004 GN=mcp4_3 PE=4 SV=1
A0A011QKN3	129.09	18	0.00E+00	4	33052	Glutamate/aspartate periplasmic-binding protein OS=Candidatus Accumulibacter sp. BA-93 OX=1454004 GN=gltI_2 PE=4 SV=1
A0A011P8L4	127.68	9	0.00E+00	4	55288	L-threonine dehydratase OS=Candidatus Accumulibacter sp. BA-93 OX=1454004 GN=ilvA PE=3 SV=1
A0A011QG78	127.41	24	6.22E6	4	20919	Hydrogenase transcriptional regulatory protein hupR1 OS=Candidatus Accumulibacter sp. BA-93 OX=1454004 GN=hupR1_3 PE=4 SV=1
A0A011QNG7	127.35	33	1.63E8	4	9987	30S ribosomal protein S17 OS=Candidatus Accumulibacter sp. BA-93 OX=1454004 GN=rpsQ PE=3 SV=1
A0A011P9Z2	126.14	43	8.05E7	4	11042	Uncharacterized protein OS=Candidatus Accumulibacter sp. BA-93 OX=1454004 GN=AW11_03877 PE=4 SV=1
A0A011QIM9	125.94	30	9.73E5	4	20268	Peptidyl-prolyl cis-trans isomerase OS=Candidatus Accumulibacter sp. BA-93 OX=1454004 GN=AW11_01712 PE=3 SV=1
A0A011P8E4	125.82	9	4.16E6	4	66967	Putative diguanylate cyclase AdrA OS=Candidatus Accumulibacter sp. BA-93 OX=1454004 GN=adrA PE=4 SV=1
A0A011QP64	124.75	40	0.00E+00	4	14409	Alkaline phosphatase synthesis transcriptional regulatory protein PhoP OS=Candidatus Accumulibacter sp. BA-93 OX=1454004 GN=phoP_1 PE=4 SV=1

A0A011QY10	124.44	38	1.28E6	4	17010	N5-carboxyaminoimidazole ribonucleotide mutase OS=Candidatus Accumulibacter sp. BA-93 OX=1454004 GN=purE PE=3 SV=1
A0A011QHP8	124.38	16	4.33E5	4	33404	Formyltetrahydrofolate deformylase OS=Candidatus Accumulibacter sp. BA-93 OX=1454004 GN=purU PE=3 SV=1
A0A011NM04	123.84	43	1.02E8	4	8404	30S ribosomal protein S21 OS=Candidatus Accumulibacter sp. SK-12 OX=1454001 GN=rpsU PE=3 SV=1
A0A011N782	123.84	43	1.02E8	4	8388	30S ribosomal protein S21 OS=Candidatus Accumulibacter sp. BA-92 OX=1454003 GN=rpsU PE=3 SV=1
A0A1A8XRR3	123.84	43	1.02E8	4	8404	30S ribosomal protein S21 OS=Candidatus Accumulibacter aalborgensis OX=1860102 GN=rpsU PE=3 SV=1
A0A011NRG1	123.84	43	1.02E8	4	8388	30S ribosomal protein S21 OS=Candidatus Accumulibacter sp. BA-93 OX=1454004 GN=rpsU PE=3 SV=1
C7RKY8	123.84	43	1.02E8	4	8404	30S ribosomal protein S21 OS=Accumulibacter phosphatis (strain UW-1) OX=522306 GN=rpsU PE=3 SV=1
A0A080M276	123.84	43	1.02E8	4	8422	30S ribosomal protein S21 OS=Candidatus Accumulibacter sp. SK-02 OX=1453999 GN=rpsU PE=3 SV=1
A0A084Y312	123.84	43	1.02E8	4	8422	30S ribosomal protein S21 OS=Candidatus Accumulibacter sp. SK-01 OX=1457154 GN=rpsU PE=3 SV=1
A0A080LXW5	123.84	43	1.02E8	4	8422	30S ribosomal protein S21 OS=Candidatus Accumulibacter sp. BA-91 OX=1454002 GN=rpsU PE=3 SV=1
A0A369XRC6	123.84	43	1.02E8	4	8388	30S ribosomal protein S21 OS=Candidatus Accumulibacter phosphatis OX=327160 GN=DVS81_03220 PE=4 SV=1
A0A011NL80	123.84	43	1.02E8	4	8404	30S ribosomal protein S21 OS=Candidatus Accumulibacter sp. SK-11 OX=1454000 GN=rpsU PE=3 SV=1
A0A011RHX9	123.58	39	3.3E6	4	11595	Iron-sulfur cluster assembly protein OS=Candidatus Accumulibacter sp. BA-93 OX=1454004 GN=iscA_1 PE=3 SV=1
A0A011PF63	123.58	39	3.3E6	4	11649	Iron-sulfur cluster assembly protein OS=Candidatus Accumulibacter sp. BA-93 OX=1454004 GN=iscA_2 PE=3 SV=1
A0A011QHN9	122.34	18	5.37E6	4	45211	Serine hydroxymethyltransferase OS=Candidatus Accumulibacter sp. BA-93 OX=1454004 GN=glyA2 PE=3 SV=1
A0A011RHF8	121.72	10	0.00E+00	4	56500	Long-chain-fatty-acid--CoA ligase FadD13 OS=Candidatus Accumulibacter sp. BA-93 OX=1454004 GN=AW11_00511 PE=4 SV=1
A0A011PRW6	121.62	46	1.65E7	4	14427	DNA repair and recombination protein RadA OS=Candidatus Accumulibacter sp. BA-93 OX=1454004 GN=AW11_01069 PE=4 SV=1
A0A011QIE5	121.58	25	8.64E6	4	20203	Cob(I)yrinic acid a c-diamide adenosyltransferase OS=Candidatus Accumulibacter sp. BA-93 OX=1454004 GN=yvqK PE=4 SV=1
A0A011P3Q0	120.60	21	1.05E7	4	27867	Thiazole synthase OS=Candidatus Accumulibacter sp. BA-93 OX=1454004 GN=thiG PE=3 SV=1
A0A011PDW6	120.39	22	1.55E7	4	16670	Rhodanese-like domain protein OS=Candidatus Accumulibacter sp. BA-93 OX=1454004 GN=AW11_03366 PE=4 SV=1
A0A011QKB7	120.07	36	0.00E+00	4	13582	Aspartate 1-decarboxylase OS=Candidatus Accumulibacter sp. BA-93 OX=1454004 GN=panD PE=3 SV=1
A0A369XVJ1	120.07	35	0.00E+00	4	14085	Aspartate 1-decarboxylase OS=Candidatus Accumulibacter phosphatis OX=327160 GN=DVS81_02865 PE=4 SV=1
A0A011NG78	120.07	36	0.00E+00	4	13538	Aspartate 1-decarboxylase OS=Candidatus Accumulibacter sp. BA-92 OX=1454003 GN=panD PE=3 SV=1
A0A011QHD7	119.72	9	3.87E6	4	70971	Transketolase OS=Candidatus Accumulibacter sp. BA-93 OX=1454004 GN=tktA_1 PE=3 SV=1
A0A011QAV9	118.42	17	2.28E7	4	35072	Cytoskeleton protein RodZ OS=Candidatus Accumulibacter sp. BA-93 OX=1454004 GN=rodZ PE=4 SV=1

A0A011PMP5	117.40	4	6.78E5	4	189965	4-alpha-glucanotransferase OS=Candidatus Accumulibacter sp. BA-93 OX=1454004 GN=treY PE=3 SV=1
A0A011NZD4	117.32	6	3.61E6	4	86228	Phenylalanine--tRNA ligase beta subunit OS=Candidatus Accumulibacter sp. BA-93 OX=1454004 GN=pheT PE=3 SV=1
A0A011R3P6	117.02	30	1.12E7	4	16900	Putative lactoylglutathione lyase OS=Candidatus Accumulibacter sp. BA-93 OX=1454004 GN=AW11_03384 PE=4 SV=1
A0A011PHH9	117.01	29	0.00E+00	4	17011	Phasin family protein OS=Candidatus Accumulibacter sp. BA-93 OX=1454004 GN=AW11_02793 PE=4 SV=1
A0A011QJX4	116.13	28	2.26E8	4	11604	Preprotein translocase subunit YajC OS=Candidatus Accumulibacter sp. BA-93 OX=1454004 GN=AW11_01654 PE=4 SV=1
A0A1A8XK04	115.28	9	1.6E7	4	68758	ATP-dependent zinc metalloprotease FtsH OS=Candidatus Accumulibacter aalborgensis OX=1860102 GN=ftsH PE=3 SV=1
A0A011P688	115.28	9	1.6E7	4	68829	ATP-dependent zinc metalloprotease FtsH OS=Candidatus Accumulibacter sp. BA-93 OX=1454004 GN=ftsH_1 PE=3 SV=1
A0A011PCL6	114.73	10	1.15E7	4	51647	Magnesium and cobalt efflux protein CorC OS=Candidatus Accumulibacter sp. BA-93 OX=1454004 GN=corC_6 PE=4 SV=1
A0A011Q440	114.36	23	3.95E7	4	16101	50S ribosomal protein L13 OS=Candidatus Accumulibacter sp. BA-93 OX=1454004 GN=rplM PE=3 SV=1
A0A011PXJ3	114.36	23	3.95E7	4	16101	50S ribosomal protein L13 OS=Candidatus Accumulibacter sp. BA-92 OX=1454003 GN=rplM PE=3 SV=1
A0A011QG39	113.71	19	4.93E6	4	30040	Putative metal-dependent hydrolase of the TIM-barrel fold protein OS=Candidatus Accumulibacter sp. BA-93 OX=1454004 GN=AW11_02188 PE=4 SV=1
A0A011R7Z2	113.69	16	5.43E6	4	41622	LysM domain protein OS=Candidatus Accumulibacter sp. BA-93 OX=1454004 GN=AW11_02681 PE=4 SV=1
A0A011PI70	113.29	17	0.00E+00	4	39608	Uroporphyrinogen decarboxylase OS=Candidatus Accumulibacter sp. BA-93 OX=1454004 GN=hemE PE=3 SV=1
A0A011N8P0	112.84	30	1.51E7	4	15898	Uncharacterized protein OS=Candidatus Accumulibacter sp. BA-92 OX=1454003 GN=AW10_02584 PE=4 SV=1
A0A011RJA5	112.84	30	1.51E7	4	15898	Uncharacterized protein OS=Candidatus Accumulibacter sp. BA-93 OX=1454004 GN=AW11_00069 PE=4 SV=1
A0A011QDF6	111.24	31	2.04E7	4	22936	ATP-dependent Clp protease proteolytic subunit OS=Candidatus Accumulibacter sp. BA-93 OX=1454004 GN=clpP PE=3 SV=1
A0A011QKU8	110.09	12	4.39E6	4	42352	O-succinylhomoserine sulfhydrylase OS=Candidatus Accumulibacter sp. BA-92 OX=1454003 GN=metZ PE=3 SV=1
A0A369XQ79	110.09	12	4.39E6	4	42429	O-succinylhomoserine sulfhydrylase OS=Candidatus Accumulibacter phosphatis OX=327160 GN=DVS81_10430 PE=4 SV=1
A0A011Q8C4	109.47	12	5.93E6	4	39243	Aminomethyltransferase OS=Candidatus Accumulibacter sp. BA-93 OX=1454004 GN=gcvT PE=3 SV=1
A0A011Q797	108.41	58	1.24E7	4	8879	50S ribosomal protein L28 OS=Candidatus Accumulibacter sp. BA-93 OX=1454004 GN=rpmB PE=3 SV=1
A0A011QN09	107.76	10	4.92E7	4	43942	Phosphate transporter OS=Candidatus Accumulibacter sp. BA-93 OX=1454004 GN=pitA_1 PE=3 SV=1
A0A011QMF2	107.75	12	0.00E+00	4	41199	Carbamoyl-phosphate synthase small chain OS=Candidatus Accumulibacter sp. BA-93 OX=1454004 GN=carA PE=3 SV=1
A0A011Q9H6	107.54	20	1.05E8	4	23256	Putative pit accessory protein OS=Candidatus Accumulibacter sp. BA-93 OX=1454004 GN=AW11_03385 PE=4 SV=1

A0A011RD56	107.25	13	0.00E+00	4	45767	Glutamate-pyruvate aminotransferase AlaA OS=Candidatus Accumulibacter sp. BA-93 OX=1454004 GN=alaA PE=4 SV=1
A0A011NPN4	107.25	13	0.00E+00	4	45890	Glutamate-pyruvate aminotransferase AlaA OS=Candidatus Accumulibacter sp. BA-92 OX=1454003 GN=alaA PE=4 SV=1
A0A011P833	107.25	21	0.00E+00	4	22500	Inner membrane lipoprotein YiaD OS=Candidatus Accumulibacter sp. BA-93 OX=1454004 GN=yiaD_1 PE=3 SV=1
A0A011RA36	105.83	65	3.29E7	4	10102	Uncharacterized protein OS=Candidatus Accumulibacter sp. BA-93 OX=1454004 GN=AW11_02328 PE=4 SV=1
A0A011PK73	105.54	6	0.00E+00	4	91293	DNA gyrase subunit B OS=Candidatus Accumulibacter sp. BA-92 OX=1454003 GN=gyrB PE=3 SV=1
A0A011NR84	105.53	28	2.77E7	4	16617	Putative phospholipid ABC transporter-binding protein MlaD OS=Candidatus Accumulibacter sp. BA-93 OX=1454004 GN=mlaD PE=4 SV=1
A0A011QJG2	105.48	16	1.6E6	4	44805	Nitrogen fixation regulatory protein OS=Candidatus Accumulibacter sp. BA-93 OX=1454004 GN=nifL PE=4 SV=1
A0A011NY98	104.89	10	2.45E6	4	71714	Asparagine synthetase [glutamine-hydrolyzing] 1 OS=Candidatus Accumulibacter sp. SK-12 OX=1454001 GN=asnB_1 PE=4 SV=1
A0A011P1M6	103.26	17	0.00E+00	4	27974	Cell division protein FtsN OS=Candidatus Accumulibacter sp. BA-93 OX=1454004 GN=AW11_01996 PE=4 SV=1
A0A011RCC9	103.14	10	1.42E6	4	52160	Glutamate decarboxylase OS=Candidatus Accumulibacter sp. BA-93 OX=1454004 GN=gadB PE=3 SV=1
A0A011NEX9	103.14	10	1.42E6	4	52146	Glutamate decarboxylase OS=Candidatus Accumulibacter sp. BA-92 OX=1454003 GN=gadB_1 PE=3 SV=1
A0A011P914	101.59	25	0.00E+00	4	15131	OsmC-like protein OS=Candidatus Accumulibacter sp. BA-93 OX=1454004 GN=AW11_04000 PE=4 SV=1
A0A011QCP3	100.15	16	0.00E+00	4	26941	Uncharacterized protein OS=Candidatus Accumulibacter sp. BA-93 OX=1454004 GN=AW11_02830 PE=4 SV=1
A0A011Q6W9	99.92	31	0.00E+00	4	15647	NADPH-dependent 7-cyano-7-deazaguanine reductase OS=Candidatus Accumulibacter sp. BA-93 OX=1454004 GN=queF PE=3 SV=1
A0A011Q880	99.43	27	0.00E+00	4	21072	Phosphocholine transferase AnkX OS=Candidatus Accumulibacter sp. BA-93 OX=1454004 GN=ankX PE=4 SV=1
A0A011RJJ4	96.55	14	2.09E7	4	31517	Uncharacterized protein OS=Candidatus Accumulibacter sp. BA-93 OX=1454004 GN=AW11_00155 PE=4 SV=1
A0A011QJY6	95.68	17	0.00E+00	4	31485	Uncharacterized protein OS=Candidatus Accumulibacter sp. BA-93 OX=1454004 GN=AW11_01664 PE=4 SV=1
A0A011N5X8	95.38	17	5.07E6	4	18892	NADH-quinone oxidoreductase subunit I OS=Candidatus Accumulibacter sp. BA-92 OX=1454003 GN=nuoI PE=3 SV=1
A0A011RH05	95.38	17	5.07E6	4	18928	NADH-quinone oxidoreductase subunit I OS=Candidatus Accumulibacter sp. BA-93 OX=1454004 GN=nuoI PE=3 SV=1
A0A1A8XRJ1	95.38	17	5.07E6	4	18826	NADH-quinone oxidoreductase subunit I OS=Candidatus Accumulibacter aalborgensis OX=1860102 GN=nuoI PE=3 SV=1
A0A011PT66	93.41	16	0.00E+00	4	39251	Ribosome-binding ATPase YchF OS=Candidatus Accumulibacter sp. BA-93 OX=1454004 GN=ychF PE=3 SV=1
A0A011NCH4	93.41	16	0.00E+00	4	39212	Ribosome-binding ATPase YchF OS=Candidatus Accumulibacter sp. BA-92 OX=1454003 GN=ychF PE=3 SV=1

A0A011N8T1	93.26	10	0.00E+00	4	50202	Biotin carboxylase OS=Candidatus Accumulibacter sp. BA-92 OX=1454003 GN=accC PE=4 SV=1
A0A011QWR6	93.22	20	0.00E+00	4	28022	Lipopolysaccharide export system ATP-binding protein LptB OS=Candidatus Accumulibacter sp. BA-92 OX=1454003 GN=lptB_1 PE=4 SV=1
A0A011P7Y0	93.22	20	0.00E+00	4	27977	Glutamine transport ATP-binding protein GlnQ OS=Candidatus Accumulibacter sp. BA-93 OX=1454004 GN=glnQ_2 PE=4 SV=1
A0A011PR65	93.18	12	0.00E+00	4	51520	Inosine-5'-monophosphate dehydrogenase OS=Candidatus Accumulibacter sp. BA-93 OX=1454004 GN=guaB_2 PE=3 SV=1
A0A011QJH2	90.79	8	0.00E+00	4	54076	Threonine synthase OS=Candidatus Accumulibacter sp. BA-93 OX=1454004 GN=thrC PE=4 SV=1
A0A369XQD4	89.34	10	8.17E6	4	38025	2-oxoacid:ferredoxin oxidoreductase subunit beta OS=Candidatus Accumulibacter phosphatis OX=327160 GN=DVS81_02900 PE=4 SV=1
A0A011QLA3	89.34	10	8.17E6	4	37992	2-oxoglutarate oxidoreductase subunit KorB OS=Candidatus Accumulibacter sp. BA-93 OX=1454004 GN=korB PE=4 SV=1
A0A011P260	89.34	10	8.17E6	4	38064	2-oxoglutarate oxidoreductase subunit KorB OS=Candidatus Accumulibacter sp. BA-92 OX=1454003 GN=korB_1 PE=4 SV=1
A0A011RIQ4	88.47	16	1.33E7	4	28249	Pyrroline-5-carboxylate reductase OS=Candidatus Accumulibacter sp. BA-93 OX=1454004 GN=proC PE=3 SV=1
A0A011PSB6	85.66	6	0.00E+00	4	63024	Arylsulfatase OS=Candidatus Accumulibacter sp. BA-92 OX=1454003 GN=atsA_3 PE=4 SV=1
A0A011NQU8	83.85	12	0.00E+00	4	34963	Uncharacterized protein OS=Candidatus Accumulibacter sp. BA-93 OX=1454004 GN=AW11_03682 PE=4 SV=1
A0A011R7Y2	83.31	7	0.00E+00	4	80298	6-phosphofructokinase OS=Candidatus Accumulibacter sp. BA-93 OX=1454004 GN=pfkA PE=4 SV=1
A0A011RA45	82.98	12	1.97E6	4	48671	Cyclic di-GMP phosphodiesterase response regulator RpfG OS=Candidatus Accumulibacter sp. BA-93 OX=1454004 GN=rpfG_8 PE=4 SV=1
A0A011QFI1	82.82	6	1.22E6	4	90375	Type IV secretion system protein virB4 OS=Candidatus Accumulibacter sp. BA-93 OX=1454004 GN=virB4 PE=4 SV=1
A0A011PM63	69.12	17	8.37E6	4	23295	Phosphoserine phosphatase OS=Candidatus Accumulibacter sp. BA-93 OX=1454004 GN=AW11_02166 PE=4 SV=1
A0A369XUF0	397.70	52	4.18E7	3	99905	Pyruvate phosphate dikinase OS=Candidatus Accumulibacter phosphatis OX=327160 GN=DVS81_08475 PE=4 SV=1
A0A011QP03	366.25	73	3.92E8	3	34128	TRAP transporter solute receptor TAXI family OS=Candidatus Accumulibacter sp. BA-93 OX=1454004 GN=AW11_00343 PE=4 SV=1
A0A011QJ18	350.25	55	9.15E7	3	72067	Chaperone protein HtpG OS=Candidatus Accumulibacter sp. BA-93 OX=1454004 GN=htpG PE=3 SV=1
A0A011PFR2	327.14	54	1.46E8	3	60836	Nitrite reductase OS=Candidatus Accumulibacter sp. BA-93 OX=1454004 GN=nirS_3 PE=4 SV=1
A0A080M1J8	298.35	40	1.23E8	3	35025	Malate dehydrogenase OS=Candidatus Accumulibacter sp. BA-91 OX=1454002 GN=mdh PE=3 SV=1
A0A369XPP8	294.02	60	1.58E9	3	15348	Chemotaxis protein OS=Candidatus Accumulibacter phosphatis OX=327160 GN=DVS81_12490 PE=4 SV=1
A0A011P7W9	294.02	60	1.58E9	3	15448	Uncharacterized protein OS=Candidatus Accumulibacter sp. BA-93 OX=1454004 GN=AW11_00394 PE=4 SV=1
A0A369XGH5	291.00	55	0.00E+00	3	35812	Type I glyceraldehyde-3-phosphate dehydrogenase OS=Candidatus Accumulibacter phosphatis OX=327160 GN=gap PE=4 SV=1
A0A369XI94	286.13	35	9.91E7	3	58976	Arylsulfatase OS=Candidatus Accumulibacter phosphatis OX=327160 GN=DVS81_14480 PE=4 SV=1

A0A011NWF1	280.44	38	2.69E7	3	67045	Aspartate--tRNA(Asp/Asn) ligase OS=Candidatus Accumulibacter sp. BA-93 OX=1454004 GN=aspS PE=3 SV=1
A0A011QKM7	278.27	64	3.81E8	3	22946	Outer membrane protein II OS=Candidatus Accumulibacter sp. BA-93 OX=1454004 GN=ompA_1 PE=3 SV=1
A0A011QJK9	264.74	63	1.58E8	3	23518	Outer membrane protein II OS=Candidatus Accumulibacter sp. BA-93 OX=1454004 GN=ompA_2 PE=3 SV=1
A0A011QMG6	263.09	54	0.00E+00	3	54370	Fumarate hydratase class I OS=Candidatus Accumulibacter sp. BA-93 OX=1454004 GN=fumB PE=3 SV=1
A0A369XHS3	250.97	40	1.29E9	3	26052	Acetoacetyl-CoA reductase OS=Candidatus Accumulibacter phosphatis OX=327160 GN=phbB PE=4 SV=1
A0A011QM83	242.83	30	1.83E7	3	53978	Probable cytosol aminopeptidase OS=Candidatus Accumulibacter sp. BA-93 OX=1454004 GN=pepA PE=3 SV=1
A0A011NHJ3	238.65	22	2.77E7	3	74230	Alpha-1 4-glucan:maltose-1-phosphate maltosyltransferase OS=Candidatus Accumulibacter sp. BA-92 OX=1454003 GN=glgE1 PE=3 SV=1
A0A011P7S3	237.54	29	1.14E7	3	51275	Succinate-semialdehyde dehydrogenase [NADP(+)] GabD OS=Candidatus Accumulibacter sp. BA-93 OX=1454004 GN=gabD_1 PE=3 SV=1
A0A011PM92	234.73	48	5.84E7	3	35692	DNA-directed RNA polymerase subunit alpha OS=Candidatus Accumulibacter sp. BA-92 OX=1454003 GN=rpoA PE=3 SV=1
A0A011PCR6	232.96	27	3.01E7	3	61015	Long-chain-fatty-acid--CoA ligase OS=Candidatus Accumulibacter sp. BA-93 OX=1454004 GN=fadD_2 PE=4 SV=1
A0A011R5A3	229.32	46	4.98E7	3	34174	Tropinesterase OS=Candidatus Accumulibacter sp. BA-93 OX=1454004 GN=AW11_03103 PE=4 SV=1
A0A011QP34	225.75	48	1.94E6	3	31549	30S ribosomal protein S3 OS=Candidatus Accumulibacter sp. BA-93 OX=1454004 GN=rpsC PE=3 SV=1
A0A011P2H4	222.19	26	1.36E7	3	55533	Circadian clock protein kinase KaiC OS=Candidatus Accumulibacter sp. BA-93 OX=1454004 GN=kaiC PE=4 SV=1
A0A011QC97	221.08	58	1.17E7	3	22704	Uncharacterized protein OS=Candidatus Accumulibacter sp. BA-93 OX=1454004 GN=AW11_02875 PE=4 SV=1
A0A011R238	217.64	44	3.03E7	3	47791	Citrate synthase OS=Candidatus Accumulibacter sp. BA-93 OX=1454004 GN=gltA_2 PE=3 SV=1
A0A011Q829	216.41	39	1.04E8	3	19883	Poly(Hydroxyalkanoate) granule-associated protein OS=Candidatus Accumulibacter sp. BA-93 OX=1454004 GN=AW11_03510 PE=4 SV=1
A0A011PCE6	204.56	47	4.18E7	3	22864	Putative phospholipid-binding protein MlaC OS=Candidatus Accumulibacter sp. BA-93 OX=1454004 GN=mlaC PE=4 SV=1
A0A011QBI0	199.47	19	1.62E7	3	60587	Dihydrolipoyl dehydrogenase OS=Candidatus Accumulibacter sp. BA-93 OX=1454004 GN=lpdA PE=3 SV=1
A0A011P305	197.67	58	9.11E7	3	15829	(R)-specific enoyl-CoA hydratase OS=Candidatus Accumulibacter sp. BA-93 OX=1454004 GN=phaJ PE=4 SV=1
A0A369XMR2	196.67	56	1.64E8	3	11884	Thioredoxin TrxA OS=Candidatus Accumulibacter phosphatis OX=327160 GN=DVS81_06975 PE=4 SV=1
A0A011NRL9	196.67	56	1.64E8	3	11884	Thioredoxin OS=Candidatus Accumulibacter sp. BA-93 OX=1454004 GN=trxA PE=3 SV=1
A0A011PQB1	196.67	56	1.64E8	3	11884	Thioredoxin OS=Candidatus Accumulibacter sp. BA-92 OX=1454003 GN=trxA PE=3 SV=1
A0A011NT71	196.48	28	5.68E6	3	29097	Uncharacterized protein OS=Candidatus Accumulibacter sp. BA-93 OX=1454004 GN=AW11_03280 PE=4 SV=1

A0A011QBB7	196.38	17	1.99E8	3	54393	Acetate transporter ActP OS=Candidatus Accumulibacter sp. BA-93 OX=1454004 GN=actP_5 PE=3 SV=1
A0A011RFF4	195.77	23	1.4E7	3	34149	Branched-chain-amino-acid aminotransferase OS=Candidatus Accumulibacter sp. BA-93 OX=1454004 GN=ilvE PE=3 SV=1
A0A011QHN3	194.06	30	0.00E+00	3	50921	Carboxy-terminal processing protease CtpB OS=Candidatus Accumulibacter sp. BA-93 OX=1454004 GN=ctpB PE=3 SV=1
A0A011R1U0	189.99	49	1.78E7	3	39351	Phospho-2-dehydro-3-deoxyheptonate aldolase OS=Candidatus Accumulibacter sp. BA-93 OX=1454004 GN=aroG PE=3 SV=1
A0A011QEA9	175.46	34	4.79E6	3	25930	Carboxylesterase 2 OS=Candidatus Accumulibacter sp. BA-93 OX=1454004 GN=estB PE=4 SV=1
A0A011RDM2	175.46	41	2.79E7	3	22745	Putative GST-like protein YibF OS=Candidatus Accumulibacter sp. BA-93 OX=1454004 GN=yibF PE=4 SV=1
A0A011QNT5	172.23	23	3.69E7	3	28455	Putative enoyl-CoA hydratase echA8 OS=Candidatus Accumulibacter sp. BA-93 OX=1454004 GN=echA8_1 PE=3 SV=1
A0A011NRN7	171.18	19	1.61E6	3	73717	Fatty acyl-CoA reductase OS=Candidatus Accumulibacter sp. BA-93 OX=1454004 GN=acr1 PE=4 SV=1
A0A011NU96	170.33	32	5.22E7	3	16052	Spore protein SP21 OS=Candidatus Accumulibacter sp. BA-93 OX=1454004 GN=hspA_2 PE=3 SV=1
A0A011NWL6	168.18	18	0.00E+00	3	89150	Lon protease OS=Candidatus Accumulibacter sp. BA-93 OX=1454004 GN=lon_1 PE=2 SV=1
A0A011QH66	159.34	22	5.85E6	3	44601	Cysteine desulfurase IscS OS=Candidatus Accumulibacter sp. BA-92 OX=1454003 GN=iscS_2 PE=3 SV=1
A0A011PTS6	159.34	22	5.85E6	3	44701	Cysteine desulfurase IscS OS=Candidatus Accumulibacter sp. BA-93 OX=1454004 GN=iscS_1 PE=3 SV=1
A0A011QPD7	158.86	30	2.77E7	3	21349	Superoxide dismutase OS=Candidatus Accumulibacter sp. BA-92 OX=1454003 GN=sodB PE=3 SV=1
A0A011QCI9	158.86	30	2.77E7	3	21259	Superoxide dismutase OS=Candidatus Accumulibacter sp. BA-93 OX=1454004 GN=sodB PE=3 SV=1
A0A011NMW6	155.95	35	1.5E8	3	20443	Putative cysteine protease YraA OS=Candidatus Accumulibacter sp. BA-93 OX=1454004 GN=yraA_2 PE=4 SV=1
A0A011RGY9	154.59	37	2.3E7	3	17091	Transcription elongation factor GreA OS=Candidatus Accumulibacter sp. BA-93 OX=1454004 GN=greA PE=3 SV=1
A0A011QAJ4	154.35	20	0.00E+00	3	33136	Glutamate/aspartate periplasmic-binding protein OS=Candidatus Accumulibacter sp. BA-93 OX=1454004 GN=gltI_3 PE=4 SV=1
A0A011NRP2	151.23	40	1.36E8	3	10323	30S ribosomal protein S15 OS=Candidatus Accumulibacter sp. BA-93 OX=1454004 GN=rpsO PE=3 SV=1
A0A011QJZ8	151.23	40	1.36E8	3	10337	30S ribosomal protein S15 OS=Candidatus Accumulibacter sp. BA-92 OX=1454003 GN=rpsO PE=3 SV=1
A0A011Q9D6	148.11	14	1.61E7	3	74153	Type IV pilus biogenesis and competence protein PilQ OS=Candidatus Accumulibacter sp. BA-93 OX=1454004 GN=pilQ PE=3 SV=1
A0A011QPA9	146.71	24	7.26E7	3	14328	Putative manganese-dependent inorganic pyrophosphatase OS=Candidatus Accumulibacter sp. BA-93 OX=1454004 GN=AW11_00256 PE=4 SV=1
A0A011PS58	146.45	10	0.00E+00	3	72481	Acetyl-coenzyme A synthetase OS=Candidatus Accumulibacter sp. BA-93 OX=1454004 GN=acsA_2 PE=4 SV=1
A0A011RDN7	144.83	17	0.00E+00	3	43798	Formyl-coenzyme A transferase OS=Candidatus Accumulibacter sp. BA-93 OX=1454004 GN=frc_2 PE=4 SV=1
A0A011QMB8	143.40	13	1.04E6	3	106204	Glycine dehydrogenase (decarboxylating) OS=Candidatus Accumulibacter sp. BA-92 OX=1454003 GN=gcvP PE=3 SV=1
A0A011Q4F2	142.98	22	2.84E6	3	41132	Cobalt-precorrin-5B C(1)-methyltransferase OS=Candidatus Accumulibacter sp. BA-93 OX=1454004 GN=cbiD PE=3 SV=1

A0A011PS53	141.08	34	4.98E7	3	31404	Electron transfer flavoprotein large subunit OS=Candidatus Accumulibacter sp. BA-93 OX=1454004 GN=etfA PE=4 SV=1
A0A011QQ52	138.74	7	0.00E+00	3	133596	Methionine synthase OS=Candidatus Accumulibacter sp. BA-93 OX=1454004 GN=metH PE=4 SV=1
A0A011QMK1	137.31	17	7.8E6	3	37489	Phosphohistidine phosphatase OS=Candidatus Accumulibacter sp. BA-93 OX=1454004 GN=AW11_00973 PE=4 SV=1
A0A011P0G7	136.62	11	1.02E6	3	51305	Pyruvate kinase OS=Candidatus Accumulibacter sp. BA-93 OX=1454004 GN=pykA PE=3 SV=1
A0A369XUM2	136.43	31	2.77E8	3	11127	Uncharacterized protein OS=Candidatus Accumulibacter phosphatis OX=327160 GN=DVS81_03870 PE=4 SV=1
A0A011QGW5	131.55	36	2.72E7	3	12292	Cell division protein ZapA OS=Candidatus Accumulibacter sp. BA-93 OX=1454004 GN=zapA PE=4 SV=1
A0A011QJ82	129.92	32	9.98E7	3	11522	Uncharacterized protein OS=Candidatus Accumulibacter sp. BA-93 OX=1454004 GN=AW11_01792 PE=4 SV=1
A0A011QWY7	129.92	32	9.98E7	3	11558	Uncharacterized protein OS=Candidatus Accumulibacter sp. BA-94 OX=1454005 GN=AW12_02608 PE=4 SV=1
A0A011QN40	127.18	8	0.00E+00	3	106253	Valine--tRNA ligase OS=Candidatus Accumulibacter sp. BA-93 OX=1454004 GN=valS PE=3 SV=1
C7RQD6	126.83	23	2.18E7	3	16111	PTS IIA-like nitrogen-regulatory protein PtsN OS=Accumulibacter phosphatis (strain UW-1) OX=522306 GN=CAP2UW1_4307 PE=4 SV=1
A0A080LTL4	126.83	23	2.18E7	3	16150	Nitrogen regulatory protein OS=Candidatus Accumulibacter sp. BA-91 OX=1454002 GN=ptsN PE=4 SV=1
A0A084Y328	126.83	23	2.18E7	3	16096	Nitrogen regulatory protein OS=Candidatus Accumulibacter sp. SK-01 OX=1457154 GN=ptsN PE=4 SV=1
A0A080M1N1	126.83	23	2.18E7	3	16078	Nitrogen regulatory protein OS=Candidatus Accumulibacter sp. SK-02 OX=1453999 GN=ptsN PE=4 SV=1
A0A011PCD6	126.83	23	2.18E7	3	16269	Nitrogen regulatory protein OS=Candidatus Accumulibacter sp. SK-11 OX=1454000 GN=ptsN PE=4 SV=1
A0A351BDZ0	126.83	23	2.18E7	3	16269	PTS IIA-like nitrogen-regulatory protein PtsN OS=Candidatus Accumulibacter sp. OX=2053492 GN=ptsN PE=4 SV=1
A0A011R1Z3	126.83	23	2.18E7	3	16257	Nitrogen regulatory protein OS=Candidatus Accumulibacter sp. BA-93 OX=1454004 GN=ptsN PE=4 SV=1
A0A011NUY2	126.83	23	2.18E7	3	16267	Nitrogen regulatory protein OS=Candidatus Accumulibacter sp. SK-12 OX=1454001 GN=ptsN PE=4 SV=1
A0A011QI17	126.83	18	2.18E7	3	19952	Nitrogen regulatory protein OS=Candidatus Accumulibacter sp. BA-92 OX=1454003 GN=ptsN PE=4 SV=1
A0A011P015	125.88	19	2.53E6	3	27228	Putative S-adenosylmethionine-dependent methyltransferase OS=Candidatus Accumulibacter sp. BA-93 OX=1454004 GN=AW11_02195 PE=4 SV=1
A0A011RGG6	125.05	22	0.00E+00	3	34111	Thioredoxin reductase OS=Candidatus Accumulibacter sp. BA-93 OX=1454004 GN=AW11_01013 PE=3 SV=1
A0A011R5H2	124.54	35	1.74E7	3	10726	30S ribosomal protein S18 OS=Candidatus Accumulibacter sp. BA-93 OX=1454004 GN=rpsR PE=3 SV=1
A0A369XRQ2	124.54	35	1.74E7	3	10712	30S ribosomal protein S18 OS=Candidatus Accumulibacter phosphatis OX=327160 GN=rpsR PE=4 SV=1
C7RPW6	124.54	35	1.74E7	3	10770	30S ribosomal protein S18 OS=Accumulibacter phosphatis (strain UW-1) OX=522306 GN=rpsR PE=3 SV=1

A0A011NKZ8	124.54	35	1.74E7	3	10810	30S ribosomal protein S18 OS=Candidatus Accumulibacter sp. SK-12 OX=1454001 GN=rpsR PE=3 SV=1
A0A1A8XNT7	124.54	35	1.74E7	3	10784	30S ribosomal protein S18 OS=Candidatus Accumulibacter aalborgensis OX=1860102 GN=rpsR PE=3 SV=1
A0A011P631	124.54	35	1.74E7	3	10810	30S ribosomal protein S18 OS=Candidatus Accumulibacter sp. BA-94 OX=1454005 GN=rpsR PE=3 SV=1
A0A080M3D3	124.54	35	1.74E7	3	10784	30S ribosomal protein S18 OS=Candidatus Accumulibacter sp. SK-02 OX=1453999 GN=rpsR PE=3 SV=1
A0A084Y2X5	124.54	35	1.74E7	3	10784	30S ribosomal protein S18 OS=Candidatus Accumulibacter sp. SK-01 OX=1457154 GN=rpsR PE=3 SV=1
A0A351BN64	124.54	35	1.74E7	3	10810	30S ribosomal protein S18 OS=Candidatus Accumulibacter sp. OX=2053492 GN=rpsR PE=4 SV=1
A0A011P6H5	124.54	35	1.74E7	3	10810	30S ribosomal protein S18 OS=Candidatus Accumulibacter sp. SK-11 OX=1454000 GN=rpsR PE=3 SV=1
A0A080LXU4	124.54	55	1.74E7	3	6749	30S ribosomal protein S18 OS=Candidatus Accumulibacter sp. BA-91 OX=1454002 GN=rpsR PE=3 SV=1
A0A011PT82	124.18	32	6.18E6	3	12263	Anti-sigma factor antagonist OS=Candidatus Accumulibacter sp. BA-93 OX=1454004 GN=btrV PE=3 SV=1
A0A011P6B3	124.12	28	1.73E7	3	12277	Preprotein translocase band 1 subunit OS=Candidatus Accumulibacter sp. BA-93 OX=1454004 GN=secG PE=4 SV=1
A0A011QHD2	122.68	7	0.00E+00	3	77837	Oligopeptidase A OS=Candidatus Accumulibacter sp. BA-93 OX=1454004 GN=prlC PE=3 SV=1
A0A011R5W3	120.43	18	9.99E6	3	22745	Outer membrane lipoprotein OS=Candidatus Accumulibacter sp. BA-93 OX=1454004 GN=AW11_02959 PE=4 SV=1
A0A011QJ73	119.88	33	6.07E7	3	12667	HIT-like protein OS=Candidatus Accumulibacter sp. BA-93 OX=1454004 GN=AW11_01498 PE=4 SV=1
A0A011PUQ7	118.15	24	6.17E6	3	15481	Uncharacterized protein OS=Candidatus Accumulibacter sp. BA-93 OX=1454004 GN=AW11_00190 PE=4 SV=1
A0A369XMR7	114.55	12	1.82E7	3	43113	Cytochrome P450 OS=Candidatus Accumulibacter phosphatis OX=327160 GN=DVS81_20880 PE=4 SV=1
A0A011RBD9	113.92	21	4.03E6	3	20311	Uncharacterized protein OS=Candidatus Accumulibacter sp. BA-93 OX=1454004 GN=AW11_02104 PE=4 SV=1
A0A011R262	112.69	18	5.68E7	3	24454	Uncharacterized protein OS=Candidatus Accumulibacter sp. BA-93 OX=1454004 GN=AW11_03607 PE=4 SV=1
A0A011PN11	111.69	11	0.00E+00	3	43249	Arginine biosynthesis bifunctional protein ArgJ OS=Candidatus Accumulibacter sp. BA-93 OX=1454004 GN=argJ PE=3 SV=1
A0A011PCV3	110.26	13	3.38E6	3	34598	Sulfate adenylyltransferase OS=Candidatus Accumulibacter sp. BA-93 OX=1454004 GN=cysD PE=3 SV=1
A0A011PN31	110.18	36	1.08E7	3	9583	30S ribosomal protein S20 OS=Candidatus Accumulibacter sp. BA-93 OX=1454004 GN=rpsT PE=3 SV=1
A0A011QM61	109.97	5	0.00E+00	3	67610	1-deoxy-D-xylulose-5-phosphate synthase OS=Candidatus Accumulibacter sp. BA-93 OX=1454004 GN=dxs PE=3 SV=1
A0A369XQW5	107.00	21	1.59E6	3	11560	30S ribosomal protein S14 OS=Candidatus Accumulibacter phosphatis OX=327160 GN=DVS81_09255 PE=4 SV=1
A0A011P7A7	107.00	21	1.59E6	3	11544	30S ribosomal protein S14 OS=Candidatus Accumulibacter sp. BA-93 OX=1454004 GN=rpsN PE=3 SV=1
A0A011P6T8	105.97	5	0.00E+00	3	81140	Ribonuclease R OS=Candidatus Accumulibacter sp. BA-93 OX=1454004 GN=rnr PE=3 SV=1
A0A011QN19	105.31	19	1.57E7	3	32546	UTP--glucose-1-phosphate uridylyltransferase OS=Candidatus Accumulibacter sp. BA-93 OX=1454004 GN=gtaB PE=3 SV=1
A0A011QPA0	105.19	6	5.16E6	3	74833	Glycine--tRNA ligase beta subunit OS=Candidatus Accumulibacter sp. BA-93 OX=1454004 GN=glyS PE=3 SV=1

A0A011PDU4	104.82	20	8.99E6	3	18645	Pilus assembly protein PilP OS=Candidatus Accumulibacter sp. BA-93 OX=1454004 GN=AW11_03346 PE=4 SV=1
A0A011PD63	103.20	45	2.2E6	3	9144	Uncharacterized protein OS=Candidatus Accumulibacter sp. BA-93 OX=1454004 GN=AW11_03442 PE=4 SV=1
A0A011QPM1	102.98	11	9.82E6	3	36136	Aliphatic amidase OS=Candidatus Accumulibacter sp. BA-93 OX=1454004 GN=amiE PE=4 SV=1
A0A011QPZ4	102.46	27	9.18E8	3	12738	Biotin carboxyl carrier protein of acetyl-CoA carboxylase OS=Candidatus Accumulibacter sp. BA-92 OX=1454003 GN=accB_1 PE=4 SV=1
A0A011PTF7	102.46	27	9.18E8	3	12738	Biotin carboxyl carrier protein of acetyl-CoA carboxylase OS=Candidatus Accumulibacter sp. BA-93 OX=1454004 GN=accB_2 PE=4 SV=1
A0A011P0Q3	101.99	50	1.98E6	3	7302	Uncharacterized protein OS=Candidatus Accumulibacter sp. BA-93 OX=1454004 GN=AW11_02158 PE=4 SV=1
A0A011QNS4	101.66	34	2.61E7	3	9931	Uncharacterized protein OS=Candidatus Accumulibacter sp. BA-93 OX=1454004 GN=AW11_00598 PE=4 SV=1
A0A011PVL5	101.66	34	2.61E7	3	10072	Uncharacterized protein OS=Candidatus Accumulibacter sp. BA-92 OX=1454003 GN=AW10_01493 PE=4 SV=1
A0A011PN23	101.64	10	2.63E6	3	35928	Anthranilate phosphoribosyltransferase OS=Candidatus Accumulibacter sp. BA-93 OX=1454004 GN=trpD PE=3 SV=1
A0A011Q7V2	100.18	29	1.97E6	3	12370	UPF0145 protein AW11_03640 OS=Candidatus Accumulibacter sp. BA-93 OX=1454004 GN=AW11_03640 PE=3 SV=1
A0A011R2G1	99.56	12	8.88E6	3	28016	Uncharacterized protein OS=Candidatus Accumulibacter sp. BA-93 OX=1454004 GN=AW11_03507 PE=4 SV=1
A0A011QDA8	98.43	21	1.7E7	3	18002	Bacterioferritin OS=Candidatus Accumulibacter sp. BA-93 OX=1454004 GN=bfr PE=3 SV=1
A0A011QL49	97.03	20	2.42E7	3	13724	Uncharacterized protein OS=Candidatus Accumulibacter sp. BA-93 OX=1454004 GN=AW11_01111 PE=4 SV=1
A0A011R3M4	96.30	30	8.52E6	3	17652	Uncharacterized protein OS=Candidatus Accumulibacter sp. BA-93 OX=1454004 GN=AW11_03364 PE=4 SV=1
A0A011PSU8	95.89	28	0.00E+00	3	14972	Universal stress protein family protein OS=Candidatus Accumulibacter sp. BA-93 OX=1454004 GN=AW11_00806 PE=4 SV=1
A0A011R5W8	95.24	19	0.00E+00	3	16328	Uncharacterized protein OS=Candidatus Accumulibacter sp. BA-93 OX=1454004 GN=AW11_02964 PE=4 SV=1
A0A011PU84	94.87	8	0.00E+00	3	55176	Uncharacterized protein OS=Candidatus Accumulibacter sp. BA-93 OX=1454004 GN=AW11_00297 PE=4 SV=1
A0A011QJU6	94.55	8	0.00E+00	3	33142	4-hydroxy-3-methylbut-2-enyl diphosphate reductase OS=Candidatus Accumulibacter sp. BA-93 OX=1454004 GN=ispH PE=3 SV=1
A0A011R280	94.48	7	0.00E+00	3	56802	Uncharacterized protein OS=Candidatus Accumulibacter sp. BA-93 OX=1454004 GN=AW11_03627 PE=4 SV=1
A0A011QPA5	94.35	11	1.33E6	3	37988	PhoH-like protein OS=Candidatus Accumulibacter sp. BA-93 OX=1454004 GN=ybeZ PE=4 SV=1
A0A011Q8K2	94.00	14	0.00E+00	3	25098	D-beta-hydroxybutyrate dehydrogenase OS=Candidatus Accumulibacter sp. BA-93 OX=1454004 GN=bdhA PE=3 SV=1
A0A011QGT3	93.80	20	7.27E6	3	15832	T4-like virus tail tube protein gp19 OS=Candidatus Accumulibacter sp. BA-93 OX=1454004 GN=AW11_02176 PE=4 SV=1

A0A369XP60	93.80	20	7.27E6	3	15908	Phage tail protein OS=Candidatus Accumulibacter phosphatis OX=327160 GN=DVS81_03820 PE=4 SV=1
A0A011QE71	93.65	32	8.27E6	3	11785	Uncharacterized protein OS=Candidatus Accumulibacter sp. BA-93 OX=1454004 GN=AW11_02528 PE=4 SV=1
A0A011PEH6	93.26	17	1.18E6	3	20944	Uncharacterized protein OS=Candidatus Accumulibacter sp. BA-93 OX=1454004 GN=AW11_03283 PE=3 SV=1
A0A011Q6W4	92.98	23	2.43E7	3	9804	DNA-binding protein HU-beta OS=Candidatus Accumulibacter sp. BA-93 OX=1454004 GN=hupB PE=3 SV=1
A0A011QP22	90.92	15	4.69E6	3	22014	Cytochrome c4 OS=Candidatus Accumulibacter sp. BA-93 OX=1454004 GN=AW11_00373 PE=4 SV=1
A0A011PKE5	90.92	15	4.69E6	3	22124	Cytochrome c4 OS=Candidatus Accumulibacter sp. BA-92 OX=1454003 GN=AW10_03867 PE=4 SV=1
A0A011PFI3	90.67	14	5.75E6	3	35523	Acetyl-coenzyme A carboxylase carboxyl transferase subunit alpha OS=Candidatus Accumulibacter sp. BA-93 OX=1454004 GN=accA PE=3 SV=1
A0A011NUF2	90.50	8	0.00E+00	3	37938	C4-dicarboxylate-binding periplasmic protein OS=Candidatus Accumulibacter sp. BA-93 OX=1454004 GN=dctP_3 PE=4 SV=1
A0A011R559	90.27	12	0.00E+00	3	34487	Putative signal peptide peptidase SppA OS=Candidatus Accumulibacter sp. BA-93 OX=1454004 GN=sppA_2 PE=4 SV=1
A0A011RAW2	90.17	6	3.16E6	3	71877	Asparagine synthetase [glutamine-hydrolyzing] 1 OS=Candidatus Accumulibacter sp. BA-93 OX=1454004 GN=asnB_2 PE=4 SV=1
A0A011PJJ1	90.17	6	3.16E6	3	72691	Asparagine synthetase [glutamine-hydrolyzing] 1 OS=Candidatus Accumulibacter sp. BA-92 OX=1454003 GN=asnB_2 PE=4 SV=1
A0A011PV89	89.99	35	2.25E7	3	11359	Uncharacterized protein OS=Candidatus Accumulibacter sp. BA-92 OX=1454003 GN=AW10_01657 PE=4 SV=1
A0A011QDV5	89.99	35	2.25E7	3	11315	Uncharacterized protein OS=Candidatus Accumulibacter sp. BA-93 OX=1454004 GN=AW11_02606 PE=4 SV=1
A0A011NZA0	89.30	11	1.46E6	3	28739	Cell division protein ZapD OS=Candidatus Accumulibacter sp. BA-93 OX=1454004 GN=zapD PE=3 SV=1
A0A369XT44	89.30	11	1.46E6	3	28792	Cell division protein ZapD OS=Candidatus Accumulibacter phosphatis OX=327160 GN=DVS81_10365 PE=4 SV=1
A0A011QKV7	89.30	11	1.46E6	3	28697	Cell division protein ZapD OS=Candidatus Accumulibacter sp. BA-92 OX=1454003 GN=zapD PE=3 SV=1
A0A011R8U0	88.83	19	3.05E7	3	15824	Flagellar assembly factor FliW OS=Candidatus Accumulibacter sp. BA-93 OX=1454004 GN=fliW PE=3 SV=1
A0A011QPJ3	88.35	6	0.00E+00	3	64355	Na/Pi-cotransporter II-related protein OS=Candidatus Accumulibacter sp. BA-93 OX=1454004 GN=AW11_00036 PE=4 SV=1
A0A011RBF7	87.69	18	0.00E+00	3	23136	Putative esterase of the alpha/beta hydrolase fold protein OS=Candidatus Accumulibacter sp. BA-93 OX=1454004 GN=AW11_02139 PE=4 SV=1
A0A011PG12	87.10	8	0.00E+00	3	48078	UDP-N-acetylmuramoyl-tripeptide--D-alanyl-D-alanine ligase OS=Candidatus Accumulibacter sp. BA-93 OX=1454004 GN=murF PE=3 SV=1
A0A011P868	86.90	54	2.32E6	3	8841	Phosphate-starvation-inducible E OS=Candidatus Accumulibacter sp. BA-93 OX=1454004 GN=AW11_00202 PE=4 SV=1
A0A011QJ22	83.98	52	0.00E+00	3	7929	Uncharacterized protein OS=Candidatus Accumulibacter sp. BA-93 OX=1454004 GN=AW11_01853 PE=4 SV=1

A0A011P0K5	83.23	10	0.00E+00	3	32509	Polyphosphate:nucleotide phosphotransferase PPK2 family OS=Candidatus Accumulibacter sp. BA-93 OX=1454004 GN=AW11_02088 PE=4 SV=1
A0A369XP83	82.99	16	5.5E6	3	12158	Phosphate-starvation-inducible protein PsiF OS=Candidatus Accumulibacter phosphatis OX=327160 GN=DVS81_03175 PE=4 SV=1
A0A011PC97	82.99	15	5.5E6	3	12571	Phosphate starvation-inducible protein PsiF OS=Candidatus Accumulibacter sp. BA-93 OX=1454004 GN=psiF PE=4 SV=1
A0A011PUR9	82.36	5	0.00E+00	3	80544	NTE family protein RssA OS=Candidatus Accumulibacter sp. BA-93 OX=1454004 GN=rssA_1 PE=4 SV=1
A0A011RFR8	81.99	4	1.6E6	3	80720	DNA polymerase III PolC-type OS=Candidatus Accumulibacter sp. BA-93 OX=1454004 GN=polC_1 PE=4 SV=1
A0A011PCZ1	81.45	18	0.00E+00	3	24672	Lactate utilization protein C OS=Candidatus Accumulibacter sp. BA-93 OX=1454004 GN=lutC PE=4 SV=1
A0A011NA38	81.08	7	3.09E6	3	56036	Amidophosphoribosyltransferase OS=Candidatus Accumulibacter sp. BA-92 OX=1454003 GN=purF PE=3 SV=1
A0A011RF17	80.85	9	0.00E+00	3	28396	UPF0246 protein AW11_01284 OS=Candidatus Accumulibacter sp. BA-93 OX=1454004 GN=AW11_01284 PE=3 SV=1
A0A011NRL3	80.57	17	0.00E+00	3	19606	Isochorismatase family protein OS=Candidatus Accumulibacter sp. BA-93 OX=1454004 GN=AW11_03491 PE=4 SV=1
A0A011PTP1	80.32	6	2.12E7	3	29062	Uncharacterized protein OS=Candidatus Accumulibacter sp. BA-93 OX=1454004 GN=AW11_00629 PE=4 SV=1
A0A011RHQ6	79.57	5	0.00E+00	3	53849	Inosine-5'-monophosphate dehydrogenase OS=Candidatus Accumulibacter sp. BA-93 OX=1454004 GN=guaB_1 PE=4 SV=1
A0A011QG27	79.47	11	0.00E+00	3	27767	Uncharacterized protein OS=Candidatus Accumulibacter sp. BA-93 OX=1454004 GN=AW11_02178 PE=4 SV=1
A0A011PGN6	79.07	10	0.00E+00	3	39578	NADH-dependent phenylglyoxylate dehydrogenase subunit epsilon OS=Candidatus Accumulibacter sp. BA-92 OX=1454003 GN=padH_2 PE=4 SV=1
A0A369XRE9	79.07	9	0.00E+00	3	43958	NAD(P)/FAD-dependent oxidoreductase OS=Candidatus Accumulibacter phosphatis OX=327160 GN=DVS81_06715 PE=4 SV=1
A0A011QJ68	79.02	16	2.39E6	3	23879	Imidazole glycerol phosphate synthase subunit HisH OS=Candidatus Accumulibacter sp. BA-93 OX=1454004 GN=hisH1_1 PE=3 SV=1
A0A011NRG5	78.92	17	2.34E7	3	18005	4-hydroxy-4-methyl-2-oxoglutarate aldolase OS=Candidatus Accumulibacter sp. BA-92 OX=1454003 GN=AW10_03393 PE=3 SV=1
A0A011RCA9	78.35	6	0.00E+00	3	49360	Outer membrane protein TolC OS=Candidatus Accumulibacter sp. BA-93 OX=1454004 GN=tolC_1 PE=4 SV=1
A0A011P4R7	75.19	9	0.00E+00	3	37196	L-threonine aldolase OS=Candidatus Accumulibacter sp. BA-93 OX=1454004 GN=ltaE PE=3 SV=1
A0A351BMU3	75.19	10	0.00E+00	3	33614	Threonine aldolase (Fragment) OS=Candidatus Accumulibacter sp. OX=2053492 GN=DCY47_19490 PE=4 SV=1
A0A011NVS8	75.19	9	0.00E+00	3	37042	L-threonine aldolase OS=Candidatus Accumulibacter sp. SK-12 OX=1454001 GN=ltaE PE=3 SV=1
A0A369XPP1	75.19	9	0.00E+00	3	37852	Low specificity L-threonine aldolase OS=Candidatus Accumulibacter phosphatis OX=327160 GN=DVS81_12855 PE=4 SV=1
A0A011QYX1	73.46	9	0.00E+00	3	40795	Multidrug transporter MdtA OS=Candidatus Accumulibacter sp. BA-93 OX=1454004 GN=mdtA_2 PE=3 SV=1

A0A011PSQ2	73.12	23	0.00E+00	3	19742	Ribosome maturation factor RimM OS=Candidatus Accumulibacter sp. BA-93 OX=1454004 GN=rimM PE=3 SV=1
A0A011P840	72.39	13	0.00E+00	3	34271	Glycine--tRNA ligase alpha subunit OS=Candidatus Accumulibacter sp. BA-93 OX=1454004 GN=glyQ PE=3 SV=1
A0A011PYR7	72.39	13	0.00E+00	3	34266	Glycine--tRNA ligase alpha subunit OS=Candidatus Accumulibacter sp. BA-92 OX=1454003 GN=glyQ PE=3 SV=1
A0A011NRB8	69.82	8	0.00E+00	3	50847	Glutamate--tRNA ligase OS=Candidatus Accumulibacter sp. BA-93 OX=1454004 GN=gltX PE=3 SV=1
A0A369XQE1	69.50	4	5.65E6	3	72505	Acyl CoA:acetate/3-ketoacid CoA transferase OS=Candidatus Accumulibacter phosphatis OX=327160 GN=DVS81_00945 PE=4 SV=1
A0A011R228	68.37	29	0.00E+00	3	15438	Uncharacterized protein OS=Candidatus Accumulibacter sp. BA-93 OX=1454004 GN=AW11_03571 PE=4 SV=1
A0A011NV53	67.20	11	6.22E6	3	27284	Uncharacterized protein OS=Candidatus Accumulibacter sp. BA-93 OX=1454004 GN=AW11_02946 PE=4 SV=1
A0A011Q9R8	65.35	4	0.00E+00	3	80075	Thioredoxin-related protein OS=Candidatus Accumulibacter sp. BA-93 OX=1454004 GN=AW11_03303 PE=4 SV=1
A0A011QP55	64.65	11	2.65E6	3	44429	Dihydroorotase OS=Candidatus Accumulibacter sp. BA-93 OX=1454004 GN=pyrC_1 PE=4 SV=1
A0A011RC63	64.15	51	2.18E6	3	8036	Uncharacterized protein OS=Candidatus Accumulibacter sp. BA-93 OX=1454004 GN=AW11_01917 PE=4 SV=1
A0A011NWE7	62.69	8	0.00E+00	3	62337	Type II traffic warden ATPase OS=Candidatus Accumulibacter sp. BA-93 OX=1454004 GN=epsE_3 PE=4 SV=1
A0A011RIB6	60.53	9	0.00E+00	3	33213	Haloalkane dehalogenase OS=Candidatus Accumulibacter sp. BA-93 OX=1454004 GN=dhlA PE=4 SV=1
A0A011QGJ2	51.80	15	0.00E+00	3	27669	Uroporphyrinogen-III synthase OS=Candidatus Accumulibacter sp. BA-93 OX=1454004 GN=hemD PE=4 SV=1
A0A011RE99	322.93	61	7.07E6	2	40909	Succinate--CoA ligase [ADP-forming] subunit beta OS=Candidatus Accumulibacter sp. BA-93 OX=1454004 GN=sucC PE=3 SV=1
A0A011P4C2	312.33	49	0.00E+00	2	92820	Aconitate hydratase B OS=Candidatus Accumulibacter sp. BA-93 OX=1454004 GN=acnB PE=3 SV=1
A0A011QCJ0	306.37	36	1.71E8	2	60217	Pyrophosphate--fructose 6-phosphate 1-phosphotransferase OS=Candidatus Accumulibacter sp. BA-93 OX=1454004 GN=pfp PE=4 SV=1
A0A369XNI1	303.20	45	2.21E7	2	92827	Bifunctional aconitate hydratase 2/2-methylisocitrate dehydratase OS=Candidatus Accumulibacter phosphatis OX=327160 GN=DVS81_09020 PE=4 SV=1
A0A011PVC7	301.38	43	1.88E8	2	54309	Poly(R)-hydroxyalkanoic acid synthase class III PhaC subunit OS=Candidatus Accumulibacter sp. BA-92 OX=1454003 GN=AW10_01576 PE=4 SV=1
A0A011RI41	297.11	47	6.81E6	2	35951	Glyceraldehyde-3-phosphate dehydrogenase OS=Candidatus Accumulibacter sp. BA-94 OX=1454005 GN=gapA PE=3 SV=1
A0A369XJC7	291.36	45	4.09E7	2	60689	Nitrite reductase OS=Candidatus Accumulibacter phosphatis OX=327160 GN=DVS81_12285 PE=4 SV=1
A0A1Q3VTR4	284.90	36	0.00E+00	2	41920	Elongation factor Tu (Fragment) OS=Candidatus Accumulibacter sp. 66-26 OX=1895689 GN=BGO63_07865 PE=3 SV=1
A0A369XS58	279.95	43	3.08E6	2	36803	Uncharacterized protein OS=Candidatus Accumulibacter phosphatis OX=327160 GN=DVS81_07145 PE=4 SV=1

A0A011P684	255.60	16	7.24E6	2	117467	Carbamoyl-phosphate synthase large chain OS=Candidatus Accumulibacter sp. BA-93 OX=1454004 GN=carB PE=3 SV=1
A0A011NWX7	252.76	31	2.59E7	2	51812	Glutamine synthetase OS=Candidatus Accumulibacter sp. BA-94 OX=1454005 GN=AW12_02065 PE=3 SV=1
A0A011QJP7	252.40	32	8.8E7	2	64776	Fumarate reductase flavoprotein subunit OS=Candidatus Accumulibacter sp. BA-93 OX=1454004 GN=frdA PE=3 SV=1
A0A011PZQ6	251.13	33	0.00E+00	2	84525	Nitrous-oxide reductase OS=Candidatus Accumulibacter sp. BA-92 OX=1454003 GN=nosZ PE=4 SV=1
A0A011PVJ8	249.40	19	2.34E7	2	92261	Arylsulfatase OS=Candidatus Accumulibacter sp. BA-92 OX=1454003 GN=atsA_2 PE=4 SV=1
A0A011P625	240.83	42	1.25E8	2	36806	Ketol-acid reductoisomerase (NADP(+)) OS=Candidatus Accumulibacter sp. BA-93 OX=1454004 GN=ilvC PE=3 SV=1
A0A011RHC4	240.64	65	1.06E8	2	26580	Triosephosphate isomerase OS=Candidatus Accumulibacter sp. BA-93 OX=1454004 GN=pgk PE=3 SV=1
A0A011N696	238.32	70	0.00E+00	2	18389	30S ribosomal protein S5 OS=Candidatus Accumulibacter sp. BA-92 OX=1454003 GN=rpsE PE=3 SV=1
A0A011QVL7	236.24	16	3.13E6	2	170150	Ferredoxin-dependent glutamate synthase 1 OS=Candidatus Accumulibacter sp. BA-92 OX=1454003 GN=gltB_2 PE=4 SV=1
A0A369XSJ8	234.37	38	1.82E7	2	29806	Uncharacterized protein OS=Candidatus Accumulibacter phosphatis OX=327160 GN=DVS81_06305 PE=4 SV=1
A0A011PVC0	233.61	30	5.13E6	2	58542	Acetolactate synthase OS=Candidatus Accumulibacter sp. BA-93 OX=1454004 GN=ilvB PE=3 SV=1
A0A011QAT1	232.13	28	1.18E7	2	84691	Nitrous-oxide reductase OS=Candidatus Accumulibacter sp. BA-93 OX=1454004 GN=nosZ PE=4 SV=1
A0A369XMJ3	217.12	28	7.05E6	2	64074	Acyl-CoA dehydrogenase OS=Candidatus Accumulibacter phosphatis OX=327160 GN=DVS81_15440 PE=4 SV=1
A0A011PVS9	216.61	40	0.00E+00	2	34309	Tropinesterase OS=Candidatus Accumulibacter sp. BA-92 OX=1454003 GN=AW10_01362 PE=4 SV=1
A0A369XKH7	215.23	16	1.22E7	2	68260	TonB-dependent receptor OS=Candidatus Accumulibacter phosphatis OX=327160 GN=DVS81_13795 PE=4 SV=1
A0A351BKT0	210.59	24	3.44E8	2	40845	Acetyl-CoA C-acyltransferase OS=Candidatus Accumulibacter sp. OX=2053492 GN=DCY47_15755 PE=4 SV=1
A0A011NBZ0	210.59	24	3.44E8	2	40845	Acetyl-CoA acetyltransferase OS=Candidatus Accumulibacter sp. SK-11 OX=1454000 GN=thlA PE=3 SV=1
A0A011QQA7	210.12	47	3.41E7	2	17595	Thiol peroxidase OS=Candidatus Accumulibacter sp. BA-93 OX=1454004 GN=tpx PE=3 SV=1
A0A011QP15	208.79	28	0.00E+00	2	41378	Acetylornithine aminotransferase OS=Candidatus Accumulibacter sp. BA-93 OX=1454004 GN=argD PE=3 SV=1
A0A011MC21	207.49	25	0.00E+00	2	54877	Fumarate hydratase class I OS=Candidatus Accumulibacter sp. SK-12 OX=1454001 GN=fumA PE=3 SV=1
A0A011PU89	206.61	19	0.00E+00	2	79810	Polyphosphate kinase OS=Candidatus Accumulibacter sp. BA-92 OX=1454003 GN=ppk PE=3 SV=1
A0A011Q262	202.53	13	0.00E+00	2	170014	NAD-specific glutamate dehydrogenase OS=Candidatus Accumulibacter sp. BA-92 OX=1454003 GN=gdhB PE=4 SV=1
A0A369XR76	202.22	12	6.22E7	2	82452	Sodium-translocating pyrophosphatase OS=Candidatus Accumulibacter phosphatis OX=327160 GN=DVS81_02950 PE=4 SV=1
A0A011Q529	192.87	41	7.96E6	2	23604	Adenylate kinase OS=Candidatus Accumulibacter sp. BA-93 OX=1454004 GN=adk PE=3 SV=1
A0A011PIB1	189.96	44	4.54E6	2	25206	3-oxoacyl-[acyl-carrier-protein] reductase FabG OS=Candidatus Accumulibacter sp. BA-93 OX=1454004 GN=fabG_3 PE=4 SV=1
A0A011PP68	188.21	35	1.01E7	2	47022	Protein HflK OS=Candidatus Accumulibacter sp. BA-93 OX=1454004 GN=hflK_2 PE=3 SV=1

A0A011P8W6	185.41	14	5.44E6	2	46145	Putative cobalt-precorrin-6Y C(15)-methyltransferase [decarboxylating] OS=Candidatus Accumulibacter sp. BA-93 OX=1454004 GN=cbiT PE=4 SV=1
C7RRX2	185.17	34	1.41E7	2	38447	HpcH/HpaI aldolase OS=Accumulibacter phosphatis (strain UW-1) OX=522306 GN=CAP2UW1_2508 PE=3 SV=1
A0A1A8XI51	185.17	34	1.41E7	2	39385	HpcH/HpaI aldolase OS=Candidatus Accumulibacter aalborgensis OX=1860102 GN=ACCAA_130031 PE=3 SV=1
A0A011NUY6	183.70	32	1.5E7	2	28233	Enoyl-[acyl-carrier-protein] reductase [NADH] OS=Candidatus Accumulibacter sp. BA-92 OX=1454003 GN=fabI_2 PE=3 SV=1
A0A011RP55	182.43	15	8.46E7	2	23651	Outer membrane protein II OS=Candidatus Accumulibacter sp. BA-94 OX=1454005 GN=ompA_1 PE=3 SV=1
A0A011PUX0	178.81	29	1.25E8	2	28777	Peptidylprolyl isomerase OS=Candidatus Accumulibacter sp. BA-92 OX=1454003 GN=AW10_01763 PE=4 SV=1
A0A011PTW7	178.09	53	0.00E+00	2	18642	50S ribosomal protein L6 OS=Candidatus Accumulibacter sp. BA-93 OX=1454004 GN=rplF PE=3 SV=1
A0A011QNY1	177.20	69	4.31E6	2	13807	Iron-sulfur cluster assembly scaffold protein IscU OS=Candidatus Accumulibacter sp. BA-93 OX=1454004 GN=nifU_1 PE=3 SV=1
A0A011Q888	174.28	26	6.08E6	2	31326	Phosphate-import protein PhnD OS=Candidatus Accumulibacter sp. BA-93 OX=1454004 GN=phnD PE=4 SV=1
A0A011RI26	173.62	50	0.00E+00	2	19888	50S ribosomal protein L5 OS=Candidatus Accumulibacter sp. BA-93 OX=1454004 GN=rplE PE=3 SV=1
A0A011QNC6	172.12	25	2.4E7	2	22979	NADH-quinone oxidoreductase subunit C OS=Candidatus Accumulibacter sp. BA-93 OX=1454004 GN=nuoC1 PE=3 SV=1
A0A011PM19	171.64	14	8.74E6	2	81827	Uncharacterized protein OS=Candidatus Accumulibacter sp. BA-93 OX=1454004 GN=AW11_02111 PE=4 SV=1
A0A011Q552	170.77	27	0.00E+00	2	41595	Acyl-CoA dehydrogenase OS=Candidatus Accumulibacter sp. BA-93 OX=1454004 GN=acdA_2 PE=3 SV=1
A0A011NNT0	166.22	24	5.15E6	2	42802	Carbonic anhydrase 2 OS=Candidatus Accumulibacter sp. BA-93 OX=1454004 GN=can PE=4 SV=1
A0A011QFB8	163.53	22	8.41E6	2	43157	Tryptophan synthase beta chain OS=Candidatus Accumulibacter sp. BA-93 OX=1454004 GN=trpB_2 PE=3 SV=1
A0A011NEG8	162.54	18	3.88E6	2	80687	Glycogen debranching enzyme OS=Candidatus Accumulibacter sp. BA-92 OX=1454003 GN=glgX_2 PE=3 SV=1
A0A011Q4K1	159.15	43	1.7E7	2	12393	Putative iron-sulfur cluster insertion protein ErpA OS=Candidatus Accumulibacter sp. BA-93 OX=1454004 GN=erpA PE=3 SV=1
A0A011QZV9	158.16	38	0.00E+00	2	22688	Putative kinase inhibitor protein OS=Candidatus Accumulibacter sp. BA-93 OX=1454004 GN=AW11_03875 PE=4 SV=1
A0A011P5S4	154.82	32	0.00E+00	2	26514	cAMP regulatory protein OS=Candidatus Accumulibacter sp. BA-93 OX=1454004 GN=crp_2 PE=4 SV=1
A0A011P3N1	154.36	33	0.00E+00	2	26375	Cyclic AMP receptor-like protein OS=Candidatus Accumulibacter sp. BA-92 OX=1454003 GN=vfr PE=4 SV=1
A0A369XKQ1	153.07	37	1.86E7	2	27591	30S ribosomal protein S2 OS=Candidatus Accumulibacter phosphatis OX=327160 GN=rpsB PE=4 SV=1
A0A011NZB4	149.56	16	4.79E6	2	38943	3-isopropylmalate dehydrogenase OS=Candidatus Accumulibacter sp. BA-93 OX=1454004 GN=leuB_1 PE=3 SV=1

A0A011P924	147.33	22	2.61E6	2	36374	Phosphoribosylformylglycinamidine cyclo-ligase OS=Candidatus Accumulibacter sp. BA-93 OX=1454004 GN=purM PE=3 SV=1
A0A011QEG1	142.95	16	0.00E+00	2	70734	Acetate CoA-transferase YdiF OS=Candidatus Accumulibacter sp. BA-93 OX=1454004 GN=ydiF_2 PE=4 SV=1
A0A011P2Q6	140.07	11	0.00E+00	2	96445	DNA gyrase subunit A OS=Candidatus Accumulibacter sp. BA-93 OX=1454004 GN=gyrA PE=3 SV=1
A0A351BCV6	139.21	11	4.56E6	2	56525	Cation acetate symporter (Fragment) OS=Candidatus Accumulibacter sp. OX=2053492 GN=DCY47_01115 PE=4 SV=1
A0A011PBP0	139.21	11	4.56E6	2	56525	Acetate transporter ActP OS=Candidatus Accumulibacter sp. SK-11 OX=1454000 GN=actP_1 PE=3 SV=1
A0A369XK17	138.73	24	0.00E+00	2	26049	SDR family NAD(P)-dependent oxidoreductase OS=Candidatus Accumulibacter phosphatis OX=327160 GN=DVS81_12300 PE=4 SV=1
A0A011R5M2	138.73	24	0.00E+00	2	26008	3-oxoacyl-[acyl-carrier-protein] reductase FabG OS=Candidatus Accumulibacter sp. BA-93 OX=1454004 GN=fabG_5 PE=4 SV=1
A0A011Q8J0	138.63	8	2.39E6	2	105924	Vitamin B12-dependent ribonucleotide reductase OS=Candidatus Accumulibacter sp. BA-93 OX=1454004 GN=nrdZ PE=3 SV=1
A0A369XQS8	138.60	21	0.00E+00	2	36409	Phosphoribosylformylglycinamidine cyclo-ligase OS=Candidatus Accumulibacter phosphatis OX=327160 GN=DVS81_04890 PE=4 SV=1
A0A011QJC3	138.23	32	3.74E7	2	13460	Uncharacterized protein OS=Candidatus Accumulibacter sp. BA-93 OX=1454004 GN=AW11_01764 PE=4 SV=1
A0A011R2K7	137.57	24	9.02E6	2	17629	Glutathione peroxidase OS=Candidatus Accumulibacter sp. BA-93 OX=1454004 GN=AW11_03542 PE=3 SV=1
A0A011R8W3	137.46	12	1.47E9	2	12501	Uncharacterized protein OS=Candidatus Accumulibacter sp. BA-93 OX=1454004 GN=AW11_02443 PE=4 SV=1
A0A011QDE2	137.40	39	1.54E7	2	8538	Uncharacterized protein OS=Candidatus Accumulibacter sp. BA-93 OX=1454004 GN=AW11_02665 PE=4 SV=1
A0A011NXU4	136.65	18	0.00E+00	2	45128	Diaminopimelate decarboxylase OS=Candidatus Accumulibacter sp. BA-93 OX=1454004 GN=lysA PE=3 SV=1
A0A011PFB4	135.64	11	1.58E7	2	32061	Malonyl CoA-acyl carrier protein transacylase OS=Candidatus Accumulibacter sp. BA-93 OX=1454004 GN=fabD PE=3 SV=1
A0A011NJR1	135.13	17	0.00E+00	2	46903	Cobyrinate a c-diamide synthase OS=Candidatus Accumulibacter sp. BA-92 OX=1454003 GN=cobB_2 PE=3 SV=1
A0A011P741	134.92	46	0.00E+00	2	13137	Putative lactoylglutathione lyase OS=Candidatus Accumulibacter sp. BA-93 OX=1454004 GN=AW11_00646 PE=4 SV=1
A0A011P4S6	134.74	21	1.94E8	2	17676	Putative membrane protein OS=Candidatus Accumulibacter sp. BA-92 OX=1454003 GN=AW10_00427 PE=4 SV=1
A0A011QJF3	134.51	16	0.00E+00	2	46956	Homoserine dehydrogenase OS=Candidatus Accumulibacter sp. BA-93 OX=1454004 GN=hom PE=3 SV=1
A0A011PQS4	134.42	17	1.81E7	2	18268	Peptidylprolyl isomerase OS=Candidatus Accumulibacter sp. BA-92 OX=1454003 GN=slyD PE=4 SV=1
A0A011PN93	133.46	6	2.04E7	2	99111	Tetratricopeptide repeat protein OS=Candidatus Accumulibacter sp. BA-92 OX=1454003 GN=AW10_03120 PE=4 SV=1
A0A011QQ95	133.13	17	3.42E6	2	34089	Acrylyl-CoA reductase AcuI OS=Candidatus Accumulibacter sp. BA-93 OX=1454004 GN=acuI_1 PE=4 SV=1

A0A369XGB6	132.41	10	4.15E7	2	52828	Uncharacterized protein OS=Candidatus Accumulibacter phosphatis OX=327160 GN=DVS81_18385 PE=4 SV=1
A0A011PI52	132.05	10	0.00E+00	2	95621	DNA topoisomerase 3 OS=Candidatus Accumulibacter sp. BA-93 OX=1454004 GN=topB PE=4 SV=1
A0A011P0J7	130.41	19	0.00E+00	2	26430	Glucose 1-dehydrogenase 1 OS=Candidatus Accumulibacter sp. BA-93 OX=1454004 GN=gdhI PE=4 SV=1
A0A011NJR5	129.42	18	0.00E+00	2	40986	Cobalt-precorrin-5B C(1)-methyltransferase OS=Candidatus Accumulibacter sp. BA-92 OX=1454003 GN=cbiD PE=3 SV=1
A0A1Q3VUB7	126.91	8	3.7E6	2	73451	Elongation factor G OS=Candidatus Accumulibacter sp. 66-26 OX=1895689 GN=fusA PE=4 SV=1
A0A011P301	124.91	12	8.33E6	2	43334	MaoC like domain protein OS=Candidatus Accumulibacter sp. BA-93 OX=1454004 GN=AW11_01667 PE=4 SV=1
A0A011QND6	124.80	21	3.09E6	2	42188	3-ketoacyl-CoA thiolase OS=Candidatus Accumulibacter sp. BA-93 OX=1454004 GN=fadA PE=3 SV=1
A0A011Q4E9	124.61	19	0.00E+00	2	46203	Cobyrinate a c-diamide synthase OS=Candidatus Accumulibacter sp. BA-93 OX=1454004 GN=cobB_3 PE=3 SV=1
A0A011RJ62	123.57	11	9.06E6	2	30580	Tim44-like domain protein OS=Candidatus Accumulibacter sp. BA-93 OX=1454004 GN=AW11_00028 PE=4 SV=1
A0A011RB60	123.38	66	0.00E+00	2	11810	Glutaredoxin OS=Candidatus Accumulibacter sp. BA-93 OX=1454004 GN=grxD PE=3 SV=1
A0A011P5T6	123.33	27	3.5E6	2	11352	Uncharacterized protein OS=Candidatus Accumulibacter sp. BA-93 OX=1454004 GN=AW11_01027 PE=4 SV=1
A0A011QFE5	123.16	20	1.42E6	2	16934	Translation initiation factor IF-3 OS=Candidatus Accumulibacter sp. BA-93 OX=1454004 GN=infC PE=3 SV=1
C7RRP9	123.16	16	1.42E6	2	21137	Translation initiation factor IF-3 OS=Accumulibacter phosphatis (strain UW-1) OX=522306 GN=infC PE=3 SV=1
A0A011RFF9	123.09	12	0.00E+00	2	49918	Phosphomannomutase/phosphoglucomutase OS=Candidatus Accumulibacter sp. BA-93 OX=1454004 GN=algC PE=4 SV=1
A0A369XMW4	122.27	22	0.00E+00	2	35562	Ornithine carbamoyltransferase OS=Candidatus Accumulibacter phosphatis OX=327160 GN=argF PE=4 SV=1
A0A011QM66	121.73	39	3.53E7	2	9582	30S ribosomal protein S16 OS=Candidatus Accumulibacter sp. BA-93 OX=1454004 GN=rpsP PE=3 SV=1
A0A011P863	120.79	22	0.00E+00	2	15094	Uncharacterized protein OS=Candidatus Accumulibacter sp. BA-93 OX=1454004 GN=AW11_00197 PE=4 SV=1
A0A011QNC2	120.49	16	4.54E7	2	23648	General stress protein 18 OS=Candidatus Accumulibacter sp. BA-92 OX=1454003 GN=yfkM PE=4 SV=1
A0A369XUB0	117.24	3	2.58E7	2	96108	Arylsulfatase OS=Candidatus Accumulibacter phosphatis OX=327160 GN=DVS81_08235 PE=4 SV=1
A0A011P7G0	115.99	13	1.89E6	2	30612	Bacterial extracellular solute-binding protein family 3 OS=Candidatus Accumulibacter sp. BA-93 OX=1454004 GN=AW11_00249 PE=4 SV=1
C7RRU2	115.61	10	1.36E8	2	14953	17 kDa surface antigen OS=Accumulibacter phosphatis (strain UW-1) OX=522306 GN=CAP2UW1_1254 PE=4 SV=1
A0A011PRM5	113.01	24	3.39E7	2	9359	50S ribosomal protein L27 OS=Candidatus Accumulibacter sp. BA-92 OX=1454003 GN=rpmA PE=3 SV=1
A0A011NZ96	113.01	24	3.39E7	2	9391	50S ribosomal protein L27 OS=Candidatus Accumulibacter sp. BA-93 OX=1454004 GN=rpmA PE=3 SV=1
A0A369XNW5	113.01	24	3.39E7	2	9375	50S ribosomal protein L27 OS=Candidatus Accumulibacter phosphatis OX=327160 GN=DVS81_10340 PE=4 SV=1
A0A011QKH5	112.20	7	3.8E6	2	78011	Methionine--tRNA ligase OS=Candidatus Accumulibacter sp. BA-93 OX=1454004 GN=metG PE=3 SV=1

A0A011NBT0	112.20	7	3.8E6	2	77767	Methionine--tRNA ligase OS=Candidatus Accumulibacter sp. BA-92 OX=1454003 GN=metG PE=3 SV=1
A0A011QQM8	111.54	4	1.55E8	2	41597	Outer membrane porin protein 32 OS=Candidatus Accumulibacter sp. BA-92 OX=1454003 GN=AW10_01223 PE=4 SV=1
A0A369XSR1	111.54	5	1.55E8	2	38944	Porin OS=Candidatus Accumulibacter phosphatis OX=327160 GN=DVS81_01425 PE=4 SV=1
A0A011QJK6	111.34	12	3.92E6	2	39080	Phospho-2-dehydro-3-deoxyheptonate aldolase OS=Candidatus Accumulibacter sp. BA-93 OX=1454004 GN=aroF PE=3 SV=1
A0A011P6J9	111.10	16	1.4E7	2	23755	Putative cysteine protease YraA OS=Candidatus Accumulibacter sp. BA-93 OX=1454004 GN=yraA_1 PE=4 SV=1
A0A011Q823	110.88	49	6.95E6	2	9154	Acyl-CoA-binding protein OS=Candidatus Accumulibacter sp. BA-93 OX=1454004 GN=AW11_03505 PE=4 SV=1
A0A011P611	109.63	18	1.62E6	2	30519	Sulfurtransferase OS=Candidatus Accumulibacter sp. BA-93 OX=1454004 GN=AW11_00763 PE=4 SV=1
A0A011PLS4	108.25	20	4.48E6	2	31283	Porphobilinogen deaminase OS=Candidatus Accumulibacter sp. BA-93 OX=1454004 GN=hemC PE=3 SV=1
A0A011QRS8	106.61	19	2.44E7	2	14462	30S ribosomal protein S9 OS=Candidatus Accumulibacter sp. BA-92 OX=1454003 GN=rpsI PE=3 SV=1
A0A011QLP0	105.83	46	3.79E7	2	7166	Zinc-finger domain protein OS=Candidatus Accumulibacter sp. BA-93 OX=1454004 GN=AW11_01201 PE=4 SV=1
A0A011PP22	104.69	27	0.00E+00	2	17074	Putative kinase inhibitor protein OS=Candidatus Accumulibacter sp. BA-93 OX=1454004 GN=AW11_01727 PE=4 SV=1
A0A011QNQ6	103.21	8	3.05E6	2	58062	Chemotaxis regulator BdlA OS=Candidatus Accumulibacter sp. BA-93 OX=1454004 GN=bdlA_1 PE=4 SV=1
A0A1Q3VS20	103.19	10	9.54E6	2	20493	Peroxiredoxin OS=Candidatus Accumulibacter sp. 66-26 OX=1895689 GN=BGO63_09695 PE=4 SV=1
C7RQD1	103.14	6	1.25E7	2	25809	Thiol:disulfide interchange protein OS=Accumulibacter phosphatis (strain UW-1) OX=522306 GN=CAP2UW1_4302 PE=3 SV=1
A0A011NRA5	99.97	18	6.69E6	2	21504	Lipopolysaccharide export system protein LptA OS=Candidatus Accumulibacter sp. BA-93 OX=1454004 GN=lptA PE=3 SV=1
A0A011P616	99.53	9	1.12E7	2	30565	GTP cyclohydrolase FolE2 OS=Candidatus Accumulibacter sp. BA-93 OX=1454004 GN=folE2_1 PE=3 SV=1
A0A011QJN9	99.51	8	0.00E+00	2	31461	Uncharacterized protein OS=Candidatus Accumulibacter sp. BA-93 OX=1454004 GN=AW11_01449 PE=4 SV=1
A0A011QZ96	98.96	10	1.07E7	2	32673	Murein hydrolase activator NlpD OS=Candidatus Accumulibacter sp. BA-93 OX=1454004 GN=nlpD_2 PE=4 SV=1
A0A011QQ37	97.31	4	0.00E+00	2	57882	Magnesium and cobalt efflux protein CorC OS=Candidatus Accumulibacter sp. BA-93 OX=1454004 GN=corC_2 PE=4 SV=1
A0A369XNT4	97.31	5	0.00E+00	2	57342	HlyC/CorC family transporter OS=Candidatus Accumulibacter phosphatis OX=327160 GN=DVS81_10960 PE=4 SV=1
A0A011PK14	97.31	5	0.00E+00	2	57447	Magnesium and cobalt efflux protein CorC OS=Candidatus Accumulibacter sp. BA-92 OX=1454003 GN=corC_5 PE=4 SV=1
A0A011R2I6	96.67	14	3.92E6	2	19434	Uncharacterized protein OS=Candidatus Accumulibacter sp. BA-93 OX=1454004 GN=AW11_03527 PE=4 SV=1

A0A011QI63	96.54	22	0.00E+00	2	12450	Uncharacterized protein OS=Candidatus Accumulibacter sp. BA-93 OX=1454004 GN=AW11_01850 PE=4 SV=1
A0A011PJ57	96.30	16	0.00E+00	2	29990	Protein phosphatase CheZ OS=Candidatus Accumulibacter sp. BA-93 OX=1454004 GN=AW11_02410 PE=3 SV=1
A0A011R8Z1	95.75	13	0.00E+00	2	45261	Lactate utilization protein A OS=Candidatus Accumulibacter sp. BA-93 OX=1454004 GN=lutA_1 PE=4 SV=1
A0A011PF89	95.54	8	2.7E6	2	33735	Metal-dependent hydrolase OS=Candidatus Accumulibacter sp. BA-93 OX=1454004 GN=AW11_03028 PE=4 SV=1
A0A011P7W4	95.32	7	0.00E+00	2	47881	Uncharacterized protein OS=Candidatus Accumulibacter sp. BA-93 OX=1454004 GN=AW11_00389 PE=4 SV=1
A0A011R2G6	95.12	15	0.00E+00	2	17611	ProP effector OS=Candidatus Accumulibacter sp. BA-93 OX=1454004 GN=proQ PE=4 SV=1
A0A011QYR5	94.82	17	0.00E+00	2	24115	Precorrin-8X methylmutase OS=Candidatus Accumulibacter sp. BA-93 OX=1454004 GN=cobH PE=4 SV=1
A0A011PU17	94.62	18	0.00E+00	2	19050	Uncharacterized protein OS=Candidatus Accumulibacter sp. BA-93 OX=1454004 GN=AW11_00237 PE=4 SV=1
A0A011PN71	94.60	6	0.00E+00	2	38982	Uncharacterized protein OS=Candidatus Accumulibacter sp. BA-93 OX=1454004 GN=AW11_01999 PE=4 SV=1
A0A011QLA7	94.12	16	0.00E+00	2	28435	3-methyl-2-oxobutanoate hydroxymethyltransferase OS=Candidatus Accumulibacter sp. BA-93 OX=1454004 GN=panB PE=3 SV=1
A0A1A8XQG7	93.55	38	0.00E+00	2	11892	Thioredoxin OS=Candidatus Accumulibacter aalborgensis OX=1860102 GN=trxA PE=3 SV=1
A0A011P8B0	93.55	6	0.00E+00	2	55077	Alkyl hydroperoxide reductase subunit F OS=Candidatus Accumulibacter sp. BA-93 OX=1454004 GN=ahpF PE=3 SV=1
A0A011PQL5	93.26	15	1.45E7	2	23306	Ankyrin repeat protein OS=Candidatus Accumulibacter sp. BA-93 OX=1454004 GN=AW11_01216 PE=4 SV=1
A0A011QZA7	92.57	20	1.26E7	2	10984	Uncharacterized protein OS=Candidatus Accumulibacter sp. BA-93 OX=1454004 GN=AW11_03946 PE=4 SV=1
A0A011QEQ0	91.56	7	0.00E+00	2	60384	Chemotaxis regulator BdlA OS=Candidatus Accumulibacter sp. BA-93 OX=1454004 GN=bdlA_2 PE=4 SV=1
A0A084XX45	91.02	41	4.96E7	2	6068	50S ribosomal protein L33 OS=Candidatus Accumulibacter sp. SK-01 OX=1457154 GN=rpmG PE=3 SV=1
A0A1A8XJY0	91.02	41	4.96E7	2	6040	50S ribosomal protein L33 OS=Candidatus Accumulibacter aalborgensis OX=1860102 GN=rpmG PE=3 SV=1
A0A080M4C8	91.02	41	4.96E7	2	6068	50S ribosomal protein L33 OS=Candidatus Accumulibacter sp. SK-02 OX=1453999 GN=rpmG PE=3 SV=1
A0A369XSA1	91.02	41	4.96E7	2	6096	50S ribosomal protein L33 OS=Candidatus Accumulibacter phosphatis OX=327160 GN=rpmG PE=4 SV=1
A0A011NQW4	91.02	41	4.96E7	2	6068	50S ribosomal protein L33 OS=Candidatus Accumulibacter sp. BA-93 OX=1454004 GN=rpmG PE=3 SV=1
C7RTB8	91.02	41	4.96E7	2	6040	50S ribosomal protein L33 OS=Accumulibacter phosphatis (strain UW-1) OX=522306 GN=rpmG PE=3 SV=1
A0A011PG17	90.76	5	1.66E6	2	48554	UDP-N-acetylmuramate--L-alanine ligase OS=Candidatus Accumulibacter sp. BA-93 OX=1454004 GN=murC PE=3 SV=1

A0A011QLI7	90.48	18	0.00E+00	2	29388	Uncharacterized protein OS=Candidatus Accumulibacter sp. BA-92 OX=1454003 GN=AW10_02229 PE=4 SV=1
A0A011QPY7	90.48	18	0.00E+00	2	29522	Uncharacterized protein OS=Candidatus Accumulibacter sp. BA-93 OX=1454004 GN=AW11_00199 PE=4 SV=1
A0A369XTV3	90.14	21	3.1E7	2	10064	30S ribosomal protein S19 OS=Candidatus Accumulibacter phosphatis OX=327160 GN=DVS81_09300 PE=4 SV=1
A0A011QNH3	90.14	21	3.1E7	2	10064	30S ribosomal protein S19 OS=Candidatus Accumulibacter sp. BA-93 OX=1454004 GN=rpsS PE=3 SV=1
A0A084Y3A7	90.14	21	3.1E7	2	10050	30S ribosomal protein S19 OS=Candidatus Accumulibacter sp. SK-01 OX=1457154 GN=rpsS PE=3 SV=1
C7RJD7	90.14	21	3.1E7	2	10034	30S ribosomal protein S19 OS=Accumulibacter phosphatis (strain UW-1) OX=522306 GN=rpsS PE=3 SV=1
A0A011PTZ6	90.14	21	3.1E7	2	10064	30S ribosomal protein S19 OS=Candidatus Accumulibacter sp. BA-92 OX=1454003 GN=rpsS PE=3 SV=1
A0A080MAN1	90.14	21	3.1E7	2	10036	30S ribosomal protein S19 OS=Candidatus Accumulibacter sp. SK-02 OX=1453999 GN=rpsS PE=3 SV=1
A0A011PRT3	89.85	12	1.76E7	2	30594	SapC OS=Candidatus Accumulibacter sp. BA-93 OX=1454004 GN=AW11_01039 PE=4 SV=1
A0A011QCA1	89.77	21	3.99E6	2	11206	Universal stress protein family protein OS=Candidatus Accumulibacter sp. BA-93 OX=1454004 GN=AW11_02880 PE=4 SV=1
A0A011P8M7	88.97	17	4.2E6	2	18831	Uncharacterized protein OS=Candidatus Accumulibacter sp. BA-93 OX=1454004 GN=AW11_00129 PE=4 SV=1
A0A011P657	88.15	18	0.00E+00	2	19384	ATP-dependent protease subunit HslV OS=Candidatus Accumulibacter sp. BA-93 OX=1454004 GN=hslV PE=3 SV=1
A0A011PVQ4	87.95	7	0.00E+00	2	44622	4-hydroxy-3-methylbut-2-en-1-yl diphosphate synthase (flavodoxin) OS=Candidatus Accumulibacter sp. BA-92 OX=1454003 GN=ispG PE=3 SV=1
A0A011PNZ0	87.91	25	1.25E7	2	10304	Circadian clock protein KaiB OS=Candidatus Accumulibacter sp. BA-93 OX=1454004 GN=kaiB_2 PE=4 SV=1
A0A011QIT4	87.33	5	0.00E+00	2	68682	Multifunctional fusion protein OS=Candidatus Accumulibacter sp. BA-93 OX=1454004 GN=aroA PE=3 SV=1
A0A011QL36	87.30	12	0.00E+00	2	27658	Cyclic di-GMP phosphodiesterase response regulator RpfG OS=Candidatus Accumulibacter sp. BA-93 OX=1454004 GN=rpfG_6 PE=4 SV=1
A0A011PX13	87.23	22	1.85E7	2	18506	Uncharacterized protein OS=Candidatus Accumulibacter sp. BA-92 OX=1454003 GN=AW10_01204 PE=4 SV=1
A0A011PJK0	87.23	22	1.85E7	2	18520	Uncharacterized protein OS=Candidatus Accumulibacter sp. BA-93 OX=1454004 GN=AW11_02386 PE=4 SV=1
A0A011NXU3	87.03	21	0.00E+00	2	10098	FeoA domain protein OS=Candidatus Accumulibacter sp. BA-93 OX=1454004 GN=AW11_02617 PE=4 SV=1
A0A011P732	86.99	4	0.00E+00	2	60157	Uncharacterized protein OS=Candidatus Accumulibacter sp. BA-93 OX=1454004 GN=AW11_00636 PE=4 SV=1
C7RMU8	86.44	16	2.00E+06	2	18278	Transcriptional regulator BadM/Rrf2 family OS=Accumulibacter phosphatis (strain UW-1) OX=522306 GN=CAP2UW1_2019 PE=4 SV=1
A0A1A8XGR2	86.44	16	2.00E+06	2	18249	DNA-binding transcriptional repressor OS=Candidatus Accumulibacter aalborgensis OX=1860102 GN=iscR PE=4 SV=1

A0A080M9H3	86.44	16	2.00E+06	2	18078	HTH-type transcriptional regulator IscR OS=Candidatus Accumulibacter sp. SK-02 OX=1453999 GN=iscR PE=4 SV=1
A0A084Y0M8	86.44	16	2.00E+06	2	18017	HTH-type transcriptional regulator IscR OS=Candidatus Accumulibacter sp. SK-01 OX=1457154 GN=iscR PE=4 SV=1
A0A080M7P2	86.44	16	2.00E+06	2	18063	HTH-type transcriptional regulator IscR OS=Candidatus Accumulibacter sp. BA-91 OX=1454002 GN=iscR PE=4 SV=1
A0A011QAE3	86.44	16	2.00E+06	2	17890	HTH-type transcriptional regulator IscR OS=Candidatus Accumulibacter sp. BA-93 OX=1454004 GN=iscR_2 PE=4 SV=1
A0A011N1U1	86.44	16	2.00E+06	2	18111	HTH-type transcriptional regulator IscR OS=Candidatus Accumulibacter sp. SK-12 OX=1454001 GN=iscR PE=4 SV=1
A0A011P761	86.44	16	2.00E+06	2	17821	HTH-type transcriptional regulator IscR OS=Candidatus Accumulibacter sp. BA-93 OX=1454004 GN=iscR_1 PE=4 SV=1
A0A011P6F0	86.44	16	2.00E+06	2	18216	HTH-type transcriptional regulator IscR OS=Candidatus Accumulibacter sp. SK-11 OX=1454000 GN=iscR PE=4 SV=1
A0A351BJX3	86.44	16	2.00E+06	2	18216	Fe-S cluster assembly transcriptional regulator IscR OS=Candidatus Accumulibacter sp. OX=2053492 GN=iscR PE=4 SV=1
A0A369XKW3	86.44	15	2.00E+06	2	18447	Fe-S cluster assembly transcriptional regulator IscR OS=Candidatus Accumulibacter phosphatis OX=327160 GN=iscR PE=4 SV=1
A0A011PMB5	86.44	15	2.00E+06	2	18582	HTH-type transcriptional regulator IscR OS=Candidatus Accumulibacter sp. BA-92 OX=1454003 GN=iscR PE=4 SV=1
A0A011P665	86.44	18	2.00E+06	2	16473	HTH-type transcriptional regulator IscR OS=Candidatus Accumulibacter sp. BA-94 OX=1454005 GN=iscR PE=4 SV=1
A0A351BFY4	85.54	15	0.00E+00	2	30683	Acyltransferase OS=Candidatus Accumulibacter sp. OX=2053492 GN=DCY47_06835 PE=4 SV=1
A0A011P4Q2	85.47	13	8.78E7	2	13589	Uncharacterized protein OS=Candidatus Accumulibacter sp. BA-93 OX=1454004 GN=AW11_01194 PE=4 SV=1
A0A011Q8E6	85.43	14	0.00E+00	2	22840	Phosphoribosylglycinamide formyltransferase OS=Candidatus Accumulibacter sp. BA-93 OX=1454004 GN=purN PE=3 SV=1
A0A369XRJ2	85.15	2	2.72E6	2	120347	Methylmalonyl-CoA mutase OS=Candidatus Accumulibacter phosphatis OX=327160 GN=DVS81_03545 PE=4 SV=1
A0A011P5E3	84.85	4	0.00E+00	2	54842	Patatin-like phospholipase OS=Candidatus Accumulibacter sp. BA-93 OX=1454004 GN=AW11_01066 PE=4 SV=1
A0A011PG68	84.84	7	0.00E+00	2	32389	Dihydrolipoyllysine-residue acetyltransferase component of pyruvate dehydrogenase complex OS=Candidatus Accumulibacter sp. BA-93 OX=1454004 GN=aceF PE=4 SV=1
A0A080M7N9	84.10	7	3.35E6	2	35166	Hopanoid-associated sugar epimerase OS=Candidatus Accumulibacter sp. BA-91 OX=1454002 GN=AW09_001666 PE=4 SV=1
A0A011R6K8	83.65	9	4.28E6	2	42628	Formate-dependent nitrite reductase complex subunit NrfG OS=Candidatus Accumulibacter sp. BA-93 OX=1454004 GN=nrfG PE=4 SV=1
A0A011PU40	83.23	19	0.00E+00	2	16385	Uncharacterized protein OS=Candidatus Accumulibacter sp. BA-93 OX=1454004 GN=AW11_00257 PE=4 SV=1
A0A011P817	82.55	7	0.00E+00	2	34944	Universal stress protein UspG OS=Candidatus Accumulibacter sp. BA-94 OX=1454005 GN=AW12_01390 PE=4 SV=1

C7RVT9	82.50	10	0.00E+00	2	31148	Uncharacterized protein OS=Accumulibacter phosphatis (strain UW-1) OX=522306 GN=CAP2UW1_4617 PE=4 SV=1
A0A011QPJ7	81.98	11	0.00E+00	2	20607	Uncharacterized protein OS=Candidatus Accumulibacter sp. BA-93 OX=1454004 GN=AW11_00041 PE=4 SV=1
A0A011P2L8	81.79	12	4.89E6	2	22291	Plasmid pRiA4b ORF-3-like protein OS=Candidatus Accumulibacter sp. BA-93 OX=1454004 GN=AW11_01734 PE=4 SV=1
A0A011R7C7	80.98	17	0.00E+00	2	12795	Regulator of competence-specific genes OS=Candidatus Accumulibacter sp. BA-93 OX=1454004 GN=AW11_02827 PE=4 SV=1
A0A011PNH4	80.98	17	0.00E+00	2	12868	Regulator of competence-specific genes OS=Candidatus Accumulibacter sp. BA-92 OX=1454003 GN=AW10_02909 PE=4 SV=1
A0A369XK54	80.98	17	0.00E+00	2	12938	Competence protein TfoX OS=Candidatus Accumulibacter phosphatis OX=327160 GN=DVS81_18070 PE=4 SV=1
A0A011QNP7	80.60	18	0.00E+00	2	16785	6-carboxy-5 6 7 8-tetrahydropterin synthase OS=Candidatus Accumulibacter sp. BA-93 OX=1454004 GN=queD PE=3 SV=1
A0A011RDE5	79.96	8	0.00E+00	2	41399	ATP phosphoribosyltransferase regulatory subunit OS=Candidatus Accumulibacter sp. BA-93 OX=1454004 GN=hisZ PE=3 SV=1
A0A011QJH8	79.70	12	2.86E6	2	24113	Flagella basal body P-ring formation protein FlgA OS=Candidatus Accumulibacter sp. BA-93 OX=1454004 GN=flgA PE=3 SV=1
A0A011QRH4	79.70	12	2.86E6	2	24569	Flagella basal body P-ring formation protein FlgA OS=Candidatus Accumulibacter sp. BA-92 OX=1454003 GN=flgA PE=3 SV=1
A0A011P5U0	79.43	16	0.00E+00	2	14253	Putative ribonuclease VapC28 OS=Candidatus Accumulibacter sp. BA-93 OX=1454004 GN=AW11_01032 PE=4 SV=1
A0A011QKX7	79.24	12	6.12E6	2	19260	Uncharacterized protein OS=Candidatus Accumulibacter sp. BA-93 OX=1454004 GN=AW11_01169 PE=4 SV=1
A0A011QHR2	78.88	11	9.77E6	2	24469	Protein-L-isoaspartate O-methyltransferase OS=Candidatus Accumulibacter sp. BA-93 OX=1454004 GN=pcm_2 PE=4 SV=1
A0A080LYI2	78.70	13	0.00E+00	2	26942	Glucose 1-dehydrogenase 4 OS=Candidatus Accumulibacter sp. BA-91 OX=1454002 GN=gdhIV PE=4 SV=1
C7RTS5	78.70	12	0.00E+00	2	27848	Short-chain dehydrogenase/reductase SDR OS=Accumulibacter phosphatis (strain UW-1) OX=522306 GN=CAP2UW1_1587 PE=4 SV=1
A0A084Y3N9	78.70	12	0.00E+00	2	27845	Glucose 1-dehydrogenase 4 OS=Candidatus Accumulibacter sp. SK-01 OX=1457154 GN=gdhIV PE=4 SV=1
A0A080MBA4	78.70	12	0.00E+00	2	27907	Glucose 1-dehydrogenase 4 OS=Candidatus Accumulibacter sp. SK-02 OX=1453999 GN=gdhIV PE=4 SV=1
A0A011QNT2	78.53	21	0.00E+00	2	18670	Putative acetyltransferase OS=Candidatus Accumulibacter sp. BA-93 OX=1454004 GN=AW11_00608 PE=4 SV=1
A0A011PUY3	77.85	9	3.18E6	2	32245	Modulator of FtsH protease HflK OS=Candidatus Accumulibacter sp. BA-92 OX=1454003 GN=hflK_2 PE=4 SV=1
A0A011P7R6	77.85	9	3.18E6	2	32257	Modulator of FtsH protease HflK OS=Candidatus Accumulibacter sp. BA-93 OX=1454004 GN=hflK_1 PE=4 SV=1

A0A011Q876	77.35	24	0.00E+00	2	10925	Uncharacterized protein OS=Candidatus Accumulibacter sp. BA-93 OX=1454004 GN=AW11_03488 PE=4 SV=1
A0A011RGB9	76.55	19	3.23E6	2	10831	Osmotically-inducible protein Y OS=Candidatus Accumulibacter sp. BA-93 OX=1454004 GN=osmY PE=4 SV=1
A0A011Q1V7	76.55	19	3.23E6	2	10831	Osmotically-inducible protein Y OS=Candidatus Accumulibacter sp. BA-92 OX=1454003 GN=osmY PE=4 SV=1
A0A011Q811	76.50	17	2.47E7	2	12045	Ferredoxin OS=Candidatus Accumulibacter sp. BA-93 OX=1454004 GN=AW11_03495 PE=4 SV=1
A0A011Q8B3	75.68	14	2.2E6	2	27053	Phosphoadenosine phosphosulfate reductase OS=Candidatus Accumulibacter sp. BA-93 OX=1454004 GN=cysH PE=3 SV=1
A0A011QDZ8	75.62	23	0.00E+00	2	14092	Chemotaxis protein CheY OS=Candidatus Accumulibacter sp. BA-93 OX=1454004 GN=cheY_4 PE=4 SV=1
A0A011P1L3	75.62	23	0.00E+00	2	14075	Chemotaxis protein CheY OS=Candidatus Accumulibacter sp. BA-92 OX=1454003 GN=cheY_1 PE=4 SV=1
A0A011REC8	75.52	13	0.00E+00	2	25421	Ribonuclease PH OS=Candidatus Accumulibacter sp. BA-93 OX=1454004 GN=rph PE=3 SV=1
A0A369XQZ9	75.52	13	0.00E+00	2	25457	Ribonuclease PH OS=Candidatus Accumulibacter phosphatis OX=327160 GN=DVS81_04080 PE=4 SV=1
A0A011PKI3	75.25	6	0.00E+00	2	42115	Aminotransferase OS=Candidatus Accumulibacter sp. BA-93 OX=1454004 GN=aspC PE=3 SV=1
A0A011QLU3	74.86	5	0.00E+00	2	58392	Glutamine-dependent NAD(+) synthetase OS=Candidatus Accumulibacter sp. BA-93 OX=1454004 GN=nadE PE=3 SV=1
A0A011PT05	74.84	17	1.92E6	2	15241	Uncharacterized protein OS=Candidatus Accumulibacter sp. BA-92 OX=1454003 GN=AW10_01954 PE=4 SV=1
A0A011QDC5	74.84	17	1.92E6	2	15559	Uncharacterized protein OS=Candidatus Accumulibacter sp. BA-93 OX=1454004 GN=AW11_02860 PE=4 SV=1
A0A011P1G3	74.27	15	0.00E+00	2	14736	Inner membrane protein YgaP OS=Candidatus Accumulibacter sp. BA-93 OX=1454004 GN=ygaP PE=4 SV=1
A0A011PIP9	74.00	12	2.12E6	2	23479	ParA-like protein OS=Candidatus Accumulibacter sp. BA-93 OX=1454004 GN=AW11_02534 PE=4 SV=1
A0A011NZA6	73.69	15	0.00E+00	2	17624	McHr OS=Candidatus Accumulibacter sp. BA-93 OX=1454004 GN=AW11_02307 PE=4 SV=1
A0A011Q8F0	73.42	8	3.33E6	2	52219	Lactate utilization protein B OS=Candidatus Accumulibacter sp. BA-93 OX=1454004 GN=lutB PE=4 SV=1
A0A011Q5Z4	73.31	10	2.56E6	2	22465	2 3 4 5-tetrahydropyridine-2 6-dicarboxylate N-acetyltransferase OS=Candidatus Accumulibacter sp. BA-93 OX=1454004 GN=dapH PE=4 SV=1
A0A011Q899	73.05	15	0.00E+00	2	28242	Lactate utilization protein A OS=Candidatus Accumulibacter sp. BA-93 OX=1454004 GN=lutA_2 PE=4 SV=1
A0A011PSY7	73.05	15	0.00E+00	2	28012	Lactate utilization protein A OS=Candidatus Accumulibacter sp. BA-92 OX=1454003 GN=lutA_1 PE=4 SV=1
A0A011PJA6	72.19	9	0.00E+00	2	28446	Serine/threonine phosphatase stp OS=Candidatus Accumulibacter sp. BA-93 OX=1454004 GN=stp_3 PE=4 SV=1
A0A011PBR7	72.09	12	5.18E6	2	25758	Preprotein translocase subunit SecA OS=Candidatus Accumulibacter sp. BA-93 OX=1454004 GN=AW11_03719 PE=4 SV=1
A0A011QPS7	71.98	7	0.00E+00	2	42293	Ribosomal RNA large subunit methyltransferase L OS=Candidatus Accumulibacter sp. BA-93 OX=1454004 GN=rlmL PE=3 SV=1

A0A011PCL7	70.74	6	0.00E+00	2	44151	Methionine gamma-lyase OS=Candidatus Accumulibacter sp. BA-94 OX=1454005 GN=mdeA_2 PE=3 SV=1
A0A011N0F3	70.37	10	0.00E+00	2	36378	UDP-glucose 4-epimerase OS=Candidatus Accumulibacter sp. SK-11 OX=1454000 GN=galE_1 PE=4 SV=1
A0A011QIP4	70.33	11	0.00E+00	2	21272	Uncharacterized protein OS=Candidatus Accumulibacter sp. BA-93 OX=1454004 GN=AW11_01728 PE=4 SV=1
A0A011RIA8	70.23	13	3.74E7	2	14982	Uncharacterized protein OS=Candidatus Accumulibacter sp. BA-93 OX=1454004 GN=AW11_00270 PE=4 SV=1
A0A011QM55	69.93	5	6.64E6	2	24201	Uncharacterized protein OS=Candidatus Accumulibacter sp. BA-93 OX=1454004 GN=AW11_00762 PE=4 SV=1
C7RJD6	69.70	16	1.76E7	2	11807	50S ribosomal protein L22 OS=Accumulibacter phosphatis (strain UW-1) OX=522306 GN=rplV PE=3 SV=1
A0A011PTX7	69.70	15	1.76E7	2	12054	50S ribosomal protein L22 OS=Candidatus Accumulibacter sp. BA-93 OX=1454004 GN=rplV PE=3 SV=1
A0A011P1H8	69.49	20	2.87E6	2	13932	Protein ApaG OS=Candidatus Accumulibacter sp. BA-93 OX=1454004 GN=apaG PE=3 SV=1
A0A011QD81	69.42	11	0.00E+00	2	29902	Protease HtpX homolog OS=Candidatus Accumulibacter sp. BA-93 OX=1454004 GN=htpX PE=3 SV=1
A0A011Q452	69.24	15	0.00E+00	2	16065	Response regulator rcp1 OS=Candidatus Accumulibacter sp. BA-93 OX=1454004 GN=rcp1_2 PE=4 SV=1
A0A011PPW4	69.19	6	0.00E+00	2	31021	Acetylglutamate kinase OS=Candidatus Accumulibacter sp. BA-92 OX=1454003 GN=argB PE=3 SV=1
A0A011QPV0	69.19	6	0.00E+00	2	31098	Acetylglutamate kinase OS=Candidatus Accumulibacter sp. BA-93 OX=1454004 GN=argB PE=3 SV=1
A0A011QIK5	68.81	2	2.3E6	2	144320	Uncharacterized protein OS=Candidatus Accumulibacter sp. BA-93 OX=1454004 GN=AW11_01687 PE=4 SV=1
A0A011RDF7	67.70	20	0.00E+00	2	10763	Integration host factor subunit beta OS=Candidatus Accumulibacter sp. BA-93 OX=1454004 GN=ihfB_2 PE=3 SV=1
A0A369XNW8	67.70	20	0.00E+00	2	10763	Integration host factor subunit beta OS=Candidatus Accumulibacter phosphatis OX=327160 GN=DVS81_18600 PE=4 SV=1
A0A369XLR4	67.13	6	1.39E6	2	32779	RNase adapter RapZ OS=Candidatus Accumulibacter phosphatis OX=327160 GN=DVS81_18045 PE=4 SV=1
A0A011R7C3	67.13	6	1.39E6	2	32670	Nucleotide-binding protein AW11_02822 OS=Candidatus Accumulibacter sp. BA-93 OX=1454004 GN=AW11_02822 PE=3 SV=1
A0A011PF71	67.08	19	0.00E+00	2	10982	Cytochrome c4 OS=Candidatus Accumulibacter sp. BA-93 OX=1454004 GN=cycA_1 PE=4 SV=1
A0A011NUG3	66.48	14	1.08E6	2	26084	Low molecular weight protein-tyrosine-phosphatase YfkJ OS=Candidatus Accumulibacter sp. BA-93 OX=1454004 GN=yfkJ_1 PE=3 SV=1
A0A011QPK3	66.40	8	1.12E6	2	35407	Anthranilate synthase component II OS=Candidatus Accumulibacter sp. BA-92 OX=1454003 GN=trpD_2 PE=4 SV=1
A0A011Q4I8	66.40	7	1.12E6	2	35461	Glycosyl transferase family protein OS=Candidatus Accumulibacter sp. BA-93 OX=1454004 GN=AW11_03984 PE=4 SV=1
A0A084Y3U2	64.90	23	2.26E6	2	10572	Uncharacterized protein OS=Candidatus Accumulibacter sp. SK-01 OX=1457154 GN=CAPSK01_000860 PE=4 SV=1
A0A011N764	64.90	22	2.26E6	2	10706	Uncharacterized protein OS=Candidatus Accumulibacter sp. BA-92 OX=1454003 GN=AW10_03039 PE=4 SV=1

A0A369XP74	64.90	22	2.26E6	2	10777	DUF3579 domain-containing protein OS=Candidatus Accumulibacter phosphatis OX=327160 GN=DVS81_03285 PE=4 SV=1
A0A011Q7X7	64.90	22	2.26E6	2	10712	Uncharacterized protein OS=Candidatus Accumulibacter sp. BA-93 OX=1454004 GN=AW11_03583 PE=4 SV=1
A0A1Q3VQ42	64.90	21	2.26E6	2	11317	Uncharacterized protein OS=Candidatus Accumulibacter sp. 66-26 OX=1895689 GN=BGO63_14970 PE=4 SV=1
A0A080LUX5	64.90	20	2.26E6	2	11475	Uncharacterized protein OS=Candidatus Accumulibacter sp. BA-91 OX=1454002 GN=AW09_002445 PE=4 SV=1
A0A080MCR1	64.90	20	2.26E6	2	11625	Uncharacterized protein OS=Candidatus Accumulibacter sp. SK-02 OX=1453999 GN=AW06_004025 PE=4 SV=1
C7RLR4	64.90	20	2.26E6	2	11744	Uncharacterized protein OS=Accumulibacter phosphatis (strain UW-1) OX=522306 GN=CAP2UW1_0611 PE=4 SV=1
A0A011QBD9	64.72	11	0.00E+00	2	17132	Uncharacterized protein OS=Candidatus Accumulibacter sp. BA-93 OX=1454004 GN=AW11_03199 PE=4 SV=1
A0A011RD09	64.50	6	0.00E+00	2	58946	PQQ enzyme repeat protein OS=Candidatus Accumulibacter sp. BA-93 OX=1454004 GN=AW11_01763 PE=4 SV=1
A0A011QAC0	64.20	12	0.00E+00	2	22455	LPS-assembly lipoprotein LptE OS=Candidatus Accumulibacter sp. BA-93 OX=1454004 GN=lptE PE=3 SV=1
A0A011P4E1	63.39	5	0.00E+00	2	48072	Ribosomal protein S12 methylthiotransferase RimO OS=Candidatus Accumulibacter sp. BA-92 OX=1454003 GN=rimO PE=3 SV=1
A0A011P2S2	63.39	5	0.00E+00	2	48115	Ribosomal protein S12 methylthiotransferase RimO OS=Candidatus Accumulibacter sp. BA-93 OX=1454004 GN=rimO PE=3 SV=1
A0A369XGV1	63.39	5	0.00E+00	2	49208	30S ribosomal protein S12 methylthiotransferase RimO OS=Candidatus Accumulibacter phosphatis OX=327160 GN=DVS81_18645 PE=4 SV=1
A0A011Q7T1	62.96	12	0.00E+00	2	19931	AhpC/TSA family protein OS=Candidatus Accumulibacter sp. BA-93 OX=1454004 GN=AW11_03620 PE=4 SV=1
A0A011N9C4	62.84	4	0.00E+00	2	60359	CTP synthase OS=Candidatus Accumulibacter sp. BA-92 OX=1454003 GN=pyrG PE=3 SV=1
A0A011P927	62.53	2	0.00E+00	2	122750	Cyclic di-GMP phosphodiesterase Gmr OS=Candidatus Accumulibacter sp. BA-93 OX=1454004 GN=gmr_13 PE=4 SV=1
A0A011Q558	62.39	4	1.88E6	2	57773	Methylmalonyl-CoA carboxyltransferase 12S subunit OS=Candidatus Accumulibacter sp. BA-93 OX=1454004 GN=AW11_03898 PE=4 SV=1
A0A369XMC3	61.59	13	4.73E7	2	12270	50S ribosomal protein L21 OS=Candidatus Accumulibacter phosphatis OX=327160 GN=rplU PE=4 SV=1
A0A011QDP9	61.56	7	0.00E+00	2	43741	Methionine gamma-lyase OS=Candidatus Accumulibacter sp. BA-93 OX=1454004 GN=mdeA_2 PE=3 SV=1
A0A011PJA0	61.54	5	2.08E6	2	47625	Uncharacterized protein OS=Candidatus Accumulibacter sp. BA-93 OX=1454004 GN=AW11_02465 PE=4 SV=1
A0A1Q3VNZ6	60.26	16	0.00E+00	2	36534	Glyceraldehyde-3-phosphate dehydrogenase OS=Candidatus Accumulibacter sp. 66-26 OX=1895689 GN=BGO63_02775 PE=3 SV=1
A0A011Q9G9	60.15	6	0.00E+00	2	36871	Uncharacterized protein OS=Candidatus Accumulibacter sp. BA-93 OX=1454004 GN=AW11_03305 PE=4 SV=1

A0A011QLD0	59.92	8	0.00E+00	2	26107	Conjugal transfer protein TrbF OS=Candidatus Accumulibacter sp. BA-93 OX=1454004 GN=AW11_01076 PE=4 SV=1
A0A011PTR0	59.66	8	0.00E+00	2	31492	Small-conductance mechanosensitive channel OS=Candidatus Accumulibacter sp. BA-93 OX=1454004 GN=mscS_1 PE=4 SV=1
A0A011PJ13	59.57	6	5.39E6	2	50187	Porin OS=Candidatus Accumulibacter sp. BA-93 OX=1454004 GN=oprB PE=3 SV=1
A0A011QGK3	59.27	9	0.00E+00	2	28026	Probable septum site-determining protein MinC OS=Candidatus Accumulibacter sp. BA-93 OX=1454004 GN=minC PE=3 SV=1
A0A011MPI8	58.94	13	0.00E+00	2	9731	Antitoxin ParD4 OS=Candidatus Accumulibacter sp. SK-11 OX=1454000 GN=parD4 PE=4 SV=1
A0A011RGR7	58.92	15	1.1E7	2	14121	Uncharacterized protein OS=Candidatus Accumulibacter sp. BA-93 OX=1454004 GN=AW11_00764 PE=4 SV=1
A0A369XT43	58.92	15	1.1E7	2	14107	STAS/SEC14 domain-containing protein OS=Candidatus Accumulibacter phosphatis OX=327160 GN=DVS81_00695 PE=4 SV=1
A0A1A8XMP4	58.91	8	0.00E+00	2	32606	D-beta-D-heptose 7-phosphate kinase OS=Candidatus Accumulibacter aalborgensis OX=1860102 GN=rfaE PE=4 SV=1
A0A011PTQ0	58.34	9	0.00E+00	2	29392	N-acyl amino acid synthase PEP-CTERM/exosortase system-associated OS=Candidatus Accumulibacter sp. BA-93 OX=1454004 GN=AW11_00639 PE=4 SV=1
A0A011P756	56.92	11	0.00E+00	2	20482	Co-chaperone protein HscB homolog OS=Candidatus Accumulibacter sp. BA-93 OX=1454004 GN=hscB_1 PE=3 SV=1
A0A011PE99	56.22	10	0.00E+00	2	33993	Uncharacterized protein OS=Candidatus Accumulibacter sp. BA-93 OX=1454004 GN=AW11_03299 PE=4 SV=1
A0A011P7Q9	56.16	2	0.00E+00	2	143706	Phosphoribosylformylglycinamidine synthase OS=Candidatus Accumulibacter sp. BA-93 OX=1454004 GN=purL PE=3 SV=1
A0A011R903	55.73	6	0.00E+00	2	39916	Repressor protein PhoU OS=Candidatus Accumulibacter sp. BA-93 OX=1454004 GN=phoU_2 PE=4 SV=1
A0A011PQ92	55.23	11	2.88E7	2	16155	RNA polymerase-binding transcription factor DksA OS=Candidatus Accumulibacter sp. BA-92 OX=1454003 GN=dksA_2 PE=3 SV=1
A0A011PTR6	53.04	8	2.62E6	2	33460	Recombination-associated protein RdgC OS=Candidatus Accumulibacter sp. BA-93 OX=1454004 GN=rdgC PE=3 SV=1
A0A011QPX0	52.98	11	0.00E+00	2	30964	Magnesium and cobalt efflux protein CorC OS=Candidatus Accumulibacter sp. BA-93 OX=1454004 GN=corC_3 PE=4 SV=1
A0A011R8Z8	51.27	12	5.05E6	2	18113	LysM domain/BON superfamily protein OS=Candidatus Accumulibacter sp. BA-93 OX=1454004 GN=AW11_02483 PE=4 SV=1
A0A011P641	50.95	5	0.00E+00	2	52555	TPR repeat-containing protein YfgC OS=Candidatus Accumulibacter sp. BA-93 OX=1454004 GN=yfgC PE=4 SV=1
A0A011PXH3	50.76	1	1.61E7	2	221803	CHAT domain protein OS=Candidatus Accumulibacter sp. BA-92 OX=1454003 GN=AW10_01082 PE=4 SV=1
A0A011QBR7	50.58	18	2.98E6	2	20597	Phosphoheptose isomerase OS=Candidatus Accumulibacter sp. BA-93 OX=1454004 GN=gmhA PE=3 SV=1
A0A011PQ78	50.51	7	1.67E6	2	29591	Flagellar brake protein YcgR OS=Candidatus Accumulibacter sp. BA-93 OX=1454004 GN=ycgR PE=3 SV=1

A0A011RGV9	47.82	4	0.00E+00	2	46762	Anti-anti-sigma factor OS=Candidatus Accumulibacter sp. BA-93 OX=1454004 GN=AW11_00809 PE=4 SV=1
A0A011R483	47.74	3	0.00E+00	2	62240	Murein L D-transpeptidase OS=Candidatus Accumulibacter sp. BA-93 OX=1454004 GN=AW11_03276 PE=4 SV=1
A0A011MPJ2	45.79	7	0.00E+00	2	37109	Chromosome segregation protein SMC OS=Candidatus Accumulibacter sp. SK-11 OX=1454000 GN=AW07_03450 PE=4 SV=1
A0A011NP84	45.40	9	0.00E+00	2	42123	3-oxoadipyl-CoA/3-oxo-5 6-dehydrosuberyl-CoA thiolase OS=Candidatus Accumulibacter sp. BA-93 OX=1454004 GN=paaJ PE=3 SV=1
A0A011P9E0	41.45	6	0.00E+00	2	37779	Membrane-bound lytic murein transglycosylase B OS=Candidatus Accumulibacter sp. BA-93 OX=1454004 GN=mltB_2 PE=4 SV=1
A0A011NSP4	41.19	5	0.00E+00	2	39846	Nitrogen regulation protein NR(II) OS=Candidatus Accumulibacter sp. BA-93 OX=1454004 GN=glnL PE=4 SV=1
A0A011RCZ5	381.36	43	1.1E8	1	69426	Acetyl-coenzyme A synthetase OS=Candidatus Accumulibacter sp. BA-93 OX=1454004 GN=acsA_3 PE=4 SV=1
A0A369XQ55	374.72	50	2.83E6	1	47651	Isocitrate lyase OS=Candidatus Accumulibacter phosphatis OX=327160 GN=DVS81_00510 PE=4 SV=1
A0A011PSB2	373.49	37	8.56E7	1	69371	Acetyl-coenzyme A synthetase OS=Candidatus Accumulibacter sp. BA-92 OX=1454003 GN=acsA_1 PE=4 SV=1
A0A011PM83	366.75	41	0.00E+00	1	71218	Acetyl-coenzyme A synthetase OS=Candidatus Accumulibacter sp. BA-92 OX=1454003 GN=acsA_3 PE=3 SV=1
A0A011P1B4	358.09	51	0.00E+00	1	47873	Isocitrate lyase OS=Candidatus Accumulibacter sp. SK-11 OX=1454000 GN=aceA PE=4 SV=1
A0A351BEY2	358.09	51	0.00E+00	1	47822	Isocitrate lyase OS=Candidatus Accumulibacter sp. OX=2053492 GN=DCY47_04990 PE=4 SV=1
A0A351BGJ0	352.63	60	0.00E+00	1	50934	F0F1 ATP synthase subunit beta OS=Candidatus Accumulibacter sp. OX=2053492 GN=atpD PE=4 SV=1
A0A011RP08	352.63	60	0.00E+00	1	50916	ATP synthase subunit beta OS=Candidatus Accumulibacter sp. BA-94 OX=1454005 GN=atpD_1 PE=3 SV=1
A0A011PVW9	350.61	48	3.73E7	1	78471	Methylmalonyl-CoA mutase OS=Candidatus Accumulibacter sp. BA-92 OX=1454003 GN=scpA_2 PE=4 SV=1
A0A011N949	325.31	56	0.00E+00	1	34971	Malate dehydrogenase OS=Candidatus Accumulibacter sp. BA-92 OX=1454003 GN=mdh_2 PE=3 SV=1
A0A011Q659	321.78	52	1.25E8	1	35024	Malate dehydrogenase OS=Candidatus Accumulibacter sp. BA-93 OX=1454004 GN=mdh_2 PE=3 SV=1
A0A011NP96	318.34	48	0.00E+00	1	50934	ATP synthase subunit beta OS=Candidatus Accumulibacter sp. SK-12 OX=1454001 GN=atpD PE=3 SV=1
A0A011N996	307.81	39	0.00E+00	1	75285	Polyribonucleotide nucleotidyltransferase OS=Candidatus Accumulibacter sp. BA-92 OX=1454003 GN=pnp PE=3 SV=1
A0A369XL15	307.47	44	4.19E7	1	43036	Elongation factor Tu OS=Candidatus Accumulibacter phosphatis OX=327160 GN=tuf PE=4 SV=1
A0A369XHP1	303.92	42	0.00E+00	1	64689	Succinate dehydrogenase flavoprotein subunit OS=Candidatus Accumulibacter phosphatis OX=327160 GN=sdhA PE=4 SV=1
A0A011P247	301.45	40	0.00E+00	1	94640	Aconitate hydratase B OS=Candidatus Accumulibacter sp. BA-92 OX=1454003 GN=acnB PE=3 SV=1
A0A011NR05	300.67	45	7.47E6	1	77201	Elongation factor G OS=Candidatus Accumulibacter sp. BA-92 OX=1454003 GN=fusA_2 PE=3 SV=1
A0A369XU41	300.28	61	2.23E7	1	40925	Acyl-CoA dehydrogenase OS=Candidatus Accumulibacter phosphatis OX=327160 GN=DVS81_03030 PE=4 SV=1
A0A369XL94	297.06	42	4.43E8	1	43009	Branched-chain amino acid ABC transporter substrate-binding protein OS=Candidatus Accumulibacter phosphatis OX=327160 GN=DVS81_12530 PE=4 SV=1

A0A080ML82	291.54	23	1.69E7	1	72511	Acetyl-/propionyl-coenzyme A carboxylase alpha chain OS=Candidatus Accumulibacter sp. SK-02 OX=1453999 GN=accA1_1 PE=4 SV=1
A0A369XPS1	288.15	35	0.00E+00	1	126522	Maltose alpha-D-glucosyltransferase OS=Candidatus Accumulibacter phosphatis OX=327160 GN=treS PE=4 SV=1
A0A369XPJ8	287.67	54	3.67E6	1	37188	C4-dicarboxylate ABC transporter OS=Candidatus Accumulibacter phosphatis OX=327160 GN=DVS81_02135 PE=4 SV=1
A0A011NUW4	285.50	27	0.00E+00	1	106109	2-oxoglutarate dehydrogenase E1 component OS=Candidatus Accumulibacter sp. BA-92 OX=1454003 GN=sucA_2 PE=4 SV=1
A0A011PZW2	283.96	37	0.00E+00	1	61535	30S ribosomal protein S1 OS=Candidatus Accumulibacter sp. BA-92 OX=1454003 GN=rpsA PE=3 SV=1
A0A011Q5I8	282.79	56	0.00E+00	1	41012	Acyl-CoA dehydrogenase OS=Candidatus Accumulibacter sp. BA-93 OX=1454004 GN=mmgC_4 PE=3 SV=1
A0A351BCE8	282.30	39	0.00E+00	1	35076	Malate dehydrogenase OS=Candidatus Accumulibacter sp. OX=2053492 GN=DCY47_00255 PE=4 SV=1
A0A011PCV1	282.30	39	0.00E+00	1	35076	Malate dehydrogenase OS=Candidatus Accumulibacter sp. SK-11 OX=1454000 GN=mdh PE=3 SV=1
C7RRX8	277.74	16	3.84E7	1	100362	Pyruvate phosphate dikinase OS=Accumulibacter phosphatis (strain UW-1) OX=522306 GN=CAP2UW1_2514 PE=3 SV=1
A0A369XFF4	277.21	39	0.00E+00	1	72052	Molecular chaperone HtpG OS=Candidatus Accumulibacter phosphatis OX=327160 GN=DVS81_20000 PE=4 SV=1
A0A1A8XH55	275.37	17	0.00E+00	1	100315	Pyruvate phosphate dikinase OS=Candidatus Accumulibacter aalborgensis OX=1860102 GN=ppdK PE=3 SV=1
A0A011Q233	270.80	40	0.00E+00	1	42724	Leucine- isoleucine- valine- threonine- and alanine-binding protein OS=Candidatus Accumulibacter sp. BA-92 OX=1454003 GN=braC_1 PE=4 SV=1
A0A011PDN1	270.49	21	0.00E+00	1	63551	Phosphoenolpyruvate carboxykinase [GTP] OS=Candidatus Accumulibacter sp. BA-94 OX=1454005 GN=pckG_1 PE=3 SV=1
A0A011P0B3	268.50	37	9.69E5	1	127478	Trehalose synthase/amylase TreS OS=Candidatus Accumulibacter sp. BA-92 OX=1454003 GN=treS_2 PE=4 SV=1
A0A369XMA2	266.78	32	4.05E6	1	43645	Phosphoglycerate kinase OS=Candidatus Accumulibacter phosphatis OX=327160 GN=pgk PE=4 SV=1
A0A011R2D7	266.65	38	2.57E7	1	45713	Enolase OS=Candidatus Accumulibacter sp. BA-93 OX=1454004 GN=eno_2 PE=3 SV=1
A0A369XQW8	266.65	38	2.57E7	1	45748	Phosphopyruvate hydratase OS=Candidatus Accumulibacter phosphatis OX=327160 GN=DVS81_06930 PE=4 SV=1
A0A011NV29	266.65	38	2.57E7	1	45771	Enolase OS=Candidatus Accumulibacter sp. BA-92 OX=1454003 GN=eno_2 PE=3 SV=1
A0A011NSF1	264.25	20	2.1E7	1	100972	Pyruvate dehydrogenase E1 component OS=Candidatus Accumulibacter sp. SK-12 OX=1454001 GN=aceE PE=4 SV=1
C7RIP3	259.41	26	0.00E+00	1	75530	Polyribonucleotide nucleotidyltransferase OS=Accumulibacter phosphatis (strain UW-1) OX=522306 GN=pnp PE=3 SV=1
A0A369XV51	258.78	50	0.00E+00	1	40778	Class III poly(R)-hydroxyalkanoic acid synthase subunit PhaC OS=Candidatus Accumulibacter phosphatis OX=327160 GN=phaC PE=4 SV=1
A0A080M4R4	255.16	22	0.00E+00	1	50411	Enolase OS=Candidatus Accumulibacter sp. SK-02 OX=1453999 GN=eno_2 PE=3 SV=1
A0A011PTB2	254.13	35	0.00E+00	1	40557	Poly-beta-hydroxybutyrate polymerase OS=Candidatus Accumulibacter sp. BA-93 OX=1454004 GN=phbC_1 PE=4 SV=1
A0A011P7X5	251.51	25	0.00E+00	1	66250	Dihydroxy-acid dehydratase OS=Candidatus Accumulibacter sp. BA-93 OX=1454004 GN=ilvD PE=3 SV=1

A0A084Y6J0	251.51	43	6.94E8	1	28201	Elongation factor Tu OS=Candidatus Accumulibacter sp. BA-91 OX=1454002 GN=tufA PE=4 SV=1
A0A1Q3VPL9	249.66	26	0.00E+00	1	64763	Succinate dehydrogenase flavoprotein subunit OS=Candidatus Accumulibacter sp. 66-26 OX=1895689 GN=BGO63_04190 PE=3 SV=1
A0A011RA04	248.50	22	0.00E+00	1	83414	NADP-dependent malic enzyme OS=Candidatus Accumulibacter sp. BA-93 OX=1454004 GN=maeB_1 PE=4 SV=1
A0A369XN14	246.44	25	0.00E+00	1	66142	Dihydroxy-acid dehydratase OS=Candidatus Accumulibacter phosphatis OX=327160 GN=DVS81_12515 PE=4 SV=1
A0A011P483	241.94	19	5.51E6	1	74316	Alpha-1 4-glucan:maltose-1-phosphate maltosyltransferase OS=Candidatus Accumulibacter sp. BA-93 OX=1454004 GN=glgE1 PE=3 SV=1
A0A011PVY3	241.91	60	0.00E+00	1	26668	Triosephosphate isomerase OS=Candidatus Accumulibacter sp. BA-92 OX=1454003 GN=pgk PE=3 SV=1
A0A369XVD1	240.52	31	3.89E7	1	28564	Uncharacterized protein OS=Candidatus Accumulibacter phosphatis OX=327160 GN=DVS81_06435 PE=4 SV=1
A0A080LXY9	240.35	25	0.00E+00	1	40591	Beta-ketothiolase BktB OS=Candidatus Accumulibacter sp. BA-91 OX=1454002 GN=bktB PE=3 SV=1
A0A369XQ19	240.21	43	0.00E+00	1	54360	Fumarate hydratase OS=Candidatus Accumulibacter phosphatis OX=327160 GN=DVS81_00300 PE=4 SV=1
C7RU15	240.11	27	0.00E+00	1	62588	30S ribosomal protein S1 OS=Accumulibacter phosphatis (strain UW-1) OX=522306 GN=CAP2UW1_2899 PE=3 SV=1
A0A369XUC9	236.89	18	0.00E+00	1	95623	Cytochrome c OS=Candidatus Accumulibacter phosphatis OX=327160 GN=DVS81_03485 PE=4 SV=1
C7RLW8	236.49	16	0.00E+00	1	69982	Propionate/CoA ligase OS=Accumulibacter phosphatis (strain UW-1) OX=522306 GN=CAP2UW1_1921 PE=4 SV=1
A0A011RI21	235.34	67	0.00E+00	1	18388	30S ribosomal protein S5 OS=Candidatus Accumulibacter sp. BA-93 OX=1454004 GN=rpsE PE=3 SV=1
A0A369XT33	230.81	19	0.00E+00	1	83557	NADP-dependent malic enzyme OS=Candidatus Accumulibacter phosphatis OX=327160 GN=DVS81_10290 PE=4 SV=1
A0A011NJH0	229.53	36	4.18E6	1	52085	Glutamate synthase [NADPH] small chain OS=Candidatus Accumulibacter sp. BA-92 OX=1454003 GN=gltB_1 PE=4 SV=1
A0A011QHL2	227.43	24	0.00E+00	1	51303	Succinate-semialdehyde dehydrogenase [NADP(+)] GabD OS=Candidatus Accumulibacter sp. BA-92 OX=1454003 GN=gabD_3 PE=3 SV=1
A0A011PM18	226.22	23	0.00E+00	1	53915	Probable cytosol aminopeptidase OS=Candidatus Accumulibacter sp. BA-92 OX=1454003 GN=pepA PE=3 SV=1
A0A369XU66	225.71	41	1.42E6	1	36865	Ketol-acid reductoisomerase OS=Candidatus Accumulibacter phosphatis OX=327160 GN=DVS81_00625 PE=4 SV=1
A0A1Q3VTP1	223.97	19	0.00E+00	1	75814	Polyribonucleotide nucleotidyltransferase OS=Candidatus Accumulibacter sp. 66-26 OX=1895689 GN=pnp PE=3 SV=1
A0A080LTU9	222.22	15	1.76E7	1	60646	Pyrophosphate--fructose 6-phosphate 1-phosphotransferase OS=Candidatus Accumulibacter sp. BA-91 OX=1454002 GN=pfp PE=4 SV=1
A0A1A8XPW1	219.75	15	0.00E+00	1	72570	Propionyl-CoA carboxylase alpha chain mitochondrial OS=Candidatus Accumulibacter aalborgensis OX=1860102 GN=Pcca PE=4 SV=1
A0A011PNY0	219.39	53	1.65E7	1	19113	Peptidoglycan-associated protein OS=Candidatus Accumulibacter sp. BA-92 OX=1454003 GN=pal PE=3 SV=1

A0A011QF71	219.39	27	1.32E7	1	70648	Phosphomethylpyrimidine synthase OS=Candidatus Accumulibacter sp. BA-92 OX=1454003 GN=thiC PE=3 SV=1
A0A011QE93	218.67	18	7.07E6	1	91940	Cyanophycin synthetase OS=Candidatus Accumulibacter sp. BA-92 OX=1454003 GN=cphA_2 PE=4 SV=1
A0A369XNT9	217.62	26	0.00E+00	1	64912	Fumarate reductase (Quinol) flavoprotein subunit OS=Candidatus Accumulibacter phosphatis OX=327160 GN=DVS81_04145 PE=4 SV=1
C7RR96	217.44	16	0.00E+00	1	60716	Hydroxylamine reductase OS=Accumulibacter phosphatis (strain UW-1) OX=522306 GN=CAP2UW1_2489 PE=4 SV=1
C7RQZ5	216.85	17	0.00E+00	1	48871	Citrate synthase OS=Accumulibacter phosphatis (strain UW-1) OX=522306 GN=CAP2UW1_2387 PE=3 SV=1
A0A369XN95	216.34	23	6.09E6	1	58593	Acetolactate synthase large subunit OS=Candidatus Accumulibacter phosphatis OX=327160 GN=DVS81_05160 PE=4 SV=1
A0A1A8XP91	213.28	27	0.00E+00	1	37244	C4-dicarboxylate-binding periplasmic protein OS=Candidatus Accumulibacter aalborgensis OX=1860102 GN=dctP PE=4 SV=1
A0A011PGN8	212.16	32	0.00E+00	1	29628	Cytochrome c-551 OS=Candidatus Accumulibacter sp. BA-93 OX=1454004 GN=AW11_02936 PE=4 SV=1
A0A369XRW7	210.73	37	0.00E+00	1	17407	Thiol peroxidase OS=Candidatus Accumulibacter phosphatis OX=327160 GN=DVS81_05295 PE=4 SV=1
A0A011MRQ0	207.62	24	0.00E+00	1	40397	Beta-ketothiolase BktB OS=Candidatus Accumulibacter sp. SK-12 OX=1454001 GN=bktB PE=3 SV=1
A0A011RH08	207.25	34	0.00E+00	1	48030	NADH-quinone oxidoreductase subunit D OS=Candidatus Accumulibacter sp. BA-93 OX=1454004 GN=nuoD PE=3 SV=1
A0A1Q3VN89	207.16	13	0.00E+00	1	47806	Isocitrate lyase OS=Candidatus Accumulibacter sp. 66-26 OX=1895689 GN=BGO63_01795 PE=4 SV=1
C7RMU4	206.09	29	0.00E+00	1	38975	Alanine dehydrogenase OS=Accumulibacter phosphatis (strain UW-1) OX=522306 GN=CAP2UW1_2015 PE=3 SV=1
A0A011QPN7	205.78	25	0.00E+00	1	46175	sn-glycerol-3-phosphate-binding periplasmic protein UgpB OS=Candidatus Accumulibacter sp. BA-92 OX=1454003 GN=ugpB PE=4 SV=1
A0A011QD70	205.65	29	0.00E+00	1	70605	Phosphomethylpyrimidine synthase OS=Candidatus Accumulibacter sp. BA-93 OX=1454004 GN=thiC PE=3 SV=1
A0A011Q286	205.52	26	4.34E6	1	41524	Acetylornithine aminotransferase OS=Candidatus Accumulibacter sp. BA-92 OX=1454003 GN=argD PE=3 SV=1
A0A351BDL9	205.30	18	7.39E6	1	40527	Acetyl-CoA C-acyltransferase OS=Candidatus Accumulibacter sp. OX=2053492 GN=DCY47_02485 PE=4 SV=1
A0A011MQU0	205.30	18	7.39E6	1	40527	Beta-ketothiolase BktB OS=Candidatus Accumulibacter sp. SK-11 OX=1454000 GN=bktB PE=3 SV=1
A0A011PSD4	204.56	54	7.28E7	1	12183	Nitrogen regulatory protein P-II OS=Candidatus Accumulibacter sp. BA-93 OX=1454004 GN=glnB PE=3 SV=1
A0A369XJN5	204.56	54	7.28E7	1	12199	P-II family nitrogen regulator OS=Candidatus Accumulibacter phosphatis OX=327160 GN=DVS81_11705 PE=4 SV=1
A0A011PHH6	203.69	51	3.15E6	1	19126	Peptidoglycan-associated protein OS=Candidatus Accumulibacter sp. BA-93 OX=1454004 GN=pal PE=3 SV=1
A0A369XPM6	200.41	36	1.95E6	1	31417	30S ribosomal protein S3 OS=Candidatus Accumulibacter phosphatis OX=327160 GN=DVS81_09290 PE=4 SV=1

A0A351BH12	198.88	31	0.00E+00	1	36930	Ketol-acid reductoisomerase OS=Candidatus Accumulibacter sp. OX=2053492 GN=DCY47_08800 PE=4 SV=1
A0A011PPK1	198.88	31	0.00E+00	1	36944	Ketol-acid reductoisomerase (NADP(+)) OS=Candidatus Accumulibacter sp. SK-12 OX=1454001 GN=ilvC PE=3 SV=1
A0A011PIU7	198.88	26	0.00E+00	1	44004	Ketol-acid reductoisomerase (NADP(+)) OS=Candidatus Accumulibacter sp. SK-11 OX=1454000 GN=ilvC PE=3 SV=1
C7RL77	198.80	21	0.00E+00	1	48155	NADH-quinone oxidoreductase subunit F OS=Accumulibacter phosphatis (strain UW-1) OX=522306 GN=CAP2UW1_3776 PE=3 SV=1
A0A011Q1F7	198.57	25	2.27E7	1	27372	Cobalt-precorrin-4 C(11)-methyltransferase OS=Candidatus Accumulibacter sp. BA-92 OX=1454003 GN=cbiF PE=3 SV=1
A0A011Q3Y7	195.51	24	4.38E6	1	27375	Cobalt-precorrin-4 C(11)-methyltransferase OS=Candidatus Accumulibacter sp. BA-93 OX=1454004 GN=cbiF PE=3 SV=1
A0A011NUP6	195.45	28	2.66E6	1	32670	Cysteine synthase OS=Candidatus Accumulibacter sp. BA-92 OX=1454003 GN=cysK1 PE=3 SV=1
A0A011PU73	195.12	16	1.15E7	1	79584	Polyphosphate kinase OS=Candidatus Accumulibacter sp. BA-93 OX=1454004 GN=ppk PE=3 SV=1
A0A369XV17	194.67	21	0.00E+00	1	58786	2 3-bisphosphoglycerate-independent phosphoglycerate mutase OS=Candidatus Accumulibacter phosphatis OX=327160 GN=DVS81_03640 PE=4 SV=1
A0A011NM01	191.53	15	0.00E+00	1	94149	Periplasmic nitrate reductase OS=Candidatus Accumulibacter sp. BA-94 OX=1454005 GN=napA PE=3 SV=1
C7RIM1	190.75	18	0.00E+00	1	61049	AMP-dependent synthetase and ligase OS=Accumulibacter phosphatis (strain UW-1) OX=522306 GN=CAP2UW1_3331 PE=4 SV=1
A0A011NVD5	190.65	18	1.22E7	1	55571	Circadian clock protein kinase KaiC OS=Candidatus Accumulibacter sp. BA-92 OX=1454003 GN=kaiC PE=4 SV=1
A0A080LTY8	189.46	31	2.32E6	1	15595	Succinyl-CoA ligase [ADP-forming] subunit alpha OS=Candidatus Accumulibacter sp. BA-91 OX=1454002 GN=sucD PE=4 SV=1
A0A1Q3VXK4	188.75	15	0.00E+00	1	71604	Chaperone protein HtpG OS=Candidatus Accumulibacter sp. 66-26 OX=1895689 GN=htpG PE=3 SV=1
A0A011PHN5	185.42	60	2.8E6	1	11643	Nucleoid-associated protein AW11_02866 OS=Candidatus Accumulibacter sp. BA-93 OX=1454004 GN=ybaB PE=3 SV=1
C7RPC3	183.25	10	0.00E+00	1	170340	Glutamate synthase (Ferredoxin) OS=Accumulibacter phosphatis (strain UW-1) OX=522306 GN=CAP2UW1_4178 PE=4 SV=1
A0A011P786	183.19	16	0.00E+00	1	86332	Putative 3-hydroxyacyl-CoA dehydrogenase OS=Candidatus Accumulibacter sp. BA-93 OX=1454004 GN=fadN PE=4 SV=1
A0A1A8XSZ0	178.61	36	0.00E+00	1	18856	Phasin family protein OS=Candidatus Accumulibacter aalborgensis OX=1860102 GN=ACCAA_550034 PE=4 SV=1
A0A011PCC8	178.15	18	0.00E+00	1	47218	Succinyl-CoA:coenzyme A transferase OS=Candidatus Accumulibacter sp. BA-93 OX=1454004 GN=cat1 PE=4 SV=1
A0A011QQH2	177.80	14	1.73E8	1	36243	C4-dicarboxylate-binding periplasmic protein OS=Candidatus Accumulibacter sp. BA-92 OX=1454003 GN=dctP_2 PE=4 SV=1
A0A011PT14	175.67	60	0.00E+00	1	11713	Nucleoid-associated protein AW10_01964 OS=Candidatus Accumulibacter sp. BA-92 OX=1454003 GN=ybaB PE=3 SV=1
A0A011R1Y9	175.34	39	0.00E+00	1	36236	Protein RecA OS=Candidatus Accumulibacter sp. BA-93 OX=1454004 GN=recA PE=3 SV=1

A0A011P5V9	175.05	19	0.00E+00	1	81908	1 4-alpha-glucan branching enzyme GlgB OS=Candidatus Accumulibacter sp. BA-92 OX=1454003 GN=glgB_1 PE=3 SV=1
A0A011QBT5	174.84	22	0.00E+00	1	40779	Cell division protein FtsZ OS=Candidatus Accumulibacter sp. BA-93 OX=1454004 GN=ftsZ PE=3 SV=1
A0A351BJT2	174.55	18	0.00E+00	1	47771	Sulfate adenylyltransferase OS=Candidatus Accumulibacter sp. OX=2053492 GN=DCY47_13900 PE=4 SV=1
A0A080M5Q1	173.74	18	0.00E+00	1	61170	Long-chain-fatty-acid--CoA ligase OS=Candidatus Accumulibacter sp. SK-02 OX=1453999 GN=fadD PE=4 SV=1
A0A369XTI0	172.51	13	0.00E+00	1	69963	Peptidylprolyl isomerase OS=Candidatus Accumulibacter phosphatis OX=327160 GN=DVS81_10235 PE=4 SV=1
A0A011PN29	170.99	37	0.00E+00	1	22728	Putative phospholipid-binding protein MlaC OS=Candidatus Accumulibacter sp. BA-92 OX=1454003 GN=mlaC PE=4 SV=1
C7RNI1	170.70	12	0.00E+00	1	83536	Malate dehydrogenase (Oxaloacetate-decarboxylating) (NADP(+)) Phosphate acetyltransferase OS=Accumulibacter phosphatis (strain UW-1) OX=522306 GN=CAP2UW1_0772 PE=4 SV=1
A0A080M567	170.43	18	0.00E+00	1	34224	Branched-chain-amino-acid aminotransferase OS=Candidatus Accumulibacter sp. SK-02 OX=1453999 GN=ilvE PE=3 SV=1
A0A084Y5X2	169.48	9	3.07E7	1	63988	Acyl-CoA dehydrogenase OS=Candidatus Accumulibacter sp. SK-01 OX=1457154 GN=mmgC_1 PE=3 SV=1
A0A011NS52	169.34	13	0.00E+00	1	37333	C4-dicarboxylate-binding periplasmic protein OS=Candidatus Accumulibacter sp. SK-12 OX=1454001 GN=dctP PE=4 SV=1
A0A351BGX0	167.44	10	0.00E+00	1	62723	Arylsulfatase OS=Candidatus Accumulibacter sp. OX=2053492 GN=DCY47_08580 PE=4 SV=1
A0A080LWZ3	166.86	10	0.00E+00	1	63976	Acyl-CoA dehydrogenase short-chain specific OS=Candidatus Accumulibacter sp. BA-91 OX=1454002 GN=AW09_002461 PE=3 SV=1
A0A1A8XK56	166.52	36	4.29E7	1	15912	(R)-specific enoyl-CoA hydratase OS=Candidatus Accumulibacter aalborgensis OX=1860102 GN=phaJ PE=4 SV=1
A0A011MGI3	165.00	15	0.00E+00	1	63529	Acyl-CoA dehydrogenase short-chain specific OS=Candidatus Accumulibacter sp. SK-12 OX=1454001 GN=AW08_00779 PE=3 SV=1
C7RRV4	164.65	6	2.61E7	1	93198	Sulfatase OS=Accumulibacter phosphatis (strain UW-1) OX=522306 GN=CAP2UW1_1266 PE=4 SV=1
C7RJD4	164.64	34	1.18E7	1	15247	50S ribosomal protein L16 OS=Accumulibacter phosphatis (strain UW-1) OX=522306 GN=rplP PE=3 SV=1
A0A011RI31	164.64	34	1.18E7	1	15307	50S ribosomal protein L16 OS=Candidatus Accumulibacter sp. BA-93 OX=1454004 GN=rplP PE=3 SV=1
A0A1A8XD34	164.17	56	0.00E+00	1	13935	Iron-sulfur cluster assembly scaffold protein IscU OS=Candidatus Accumulibacter aalborgensis OX=1860102 GN=iscU PE=3 SV=1
A0A011P181	162.89	18	1.13E7	1	80544	Glycogen debranching enzyme OS=Candidatus Accumulibacter sp. BA-93 OX=1454004 GN=glgX_2 PE=3 SV=1
A0A369XP29	162.87	26	0.00E+00	1	30640	4-hydroxy-tetrahydrodipicolinate synthase OS=Candidatus Accumulibacter phosphatis OX=327160 GN=DVS81_04260 PE=4 SV=1
A0A011Q767	161.77	43	0.00E+00	1	24290	Dihydrolipoyllysine-residue succinyltransferase component of 2-oxoglutarate dehydrogenase complex OS=Candidatus Accumulibacter sp. BA-93 OX=1454004 GN=sucB PE=4 SV=1
A0A011QJN4	160.44	26	0.00E+00	1	46416	Adenylosuccinate synthetase OS=Candidatus Accumulibacter sp. BA-93 OX=1454004 GN=purA PE=3 SV=1

A0A011RAX2	160.33	19	1.11E6	1	34026	Tyrosine-protein kinase YwqD OS=Candidatus Accumulibacter sp. BA-93 OX=1454004 GN=ywqD PE=4 SV=1
A0A011NPH5	160.33	18	1.11E6	1	35871	Tyrosine-protein kinase YwqD OS=Candidatus Accumulibacter sp. BA-92 OX=1454003 GN=ywqD PE=4 SV=1
A0A369XUQ0	157.89	28	0.00E+00	1	41610	Isovaleryl-CoA dehydrogenase OS=Candidatus Accumulibacter phosphatis OX=327160 GN=DVS81_03015 PE=4 SV=1
A0A011P3B3	157.38	19	0.00E+00	1	43511	3-oxoacyl-[acyl-carrier-protein] synthase 2 OS=Candidatus Accumulibacter sp. BA-92 OX=1454003 GN=fabF PE=3 SV=1
A0A369XQB0	157.36	35	0.00E+00	1	22820	Glutathione S-transferase OS=Candidatus Accumulibacter phosphatis OX=327160 GN=DVS81_08515 PE=4 SV=1
C7RT75	156.08	21	0.00E+00	1	49963	Carboxyl-terminal protease OS=Accumulibacter phosphatis (strain UW-1) OX=522306 GN=CAP2UW1_1503 PE=3 SV=1
A0A351BDS9	156.00	22	0.00E+00	1	21528	2-oxoacid:acceptor oxidoreductase OS=Candidatus Accumulibacter sp. OX=2053492 GN=DCY47_02790 PE=4 SV=1
A0A011NVW3	156.00	22	0.00E+00	1	21517	NADH-dependent phenylglyoxylate dehydrogenase subunit gamma OS=Candidatus Accumulibacter sp. SK-12 OX=1454001 GN=padE PE=4 SV=1
A0A011RPK8	156.00	22	0.00E+00	1	21547	NADH-dependent phenylglyoxylate dehydrogenase subunit gamma OS=Candidatus Accumulibacter sp. BA-94 OX=1454005 GN=padE PE=4 SV=1
A0A011MUN8	156.00	22	0.00E+00	1	21528	NADH-dependent phenylglyoxylate dehydrogenase subunit gamma OS=Candidatus Accumulibacter sp. SK-11 OX=1454000 GN=padE PE=4 SV=1
A0A369XK53	155.36	23	0.00E+00	1	37863	Asparaginase OS=Candidatus Accumulibacter phosphatis OX=327160 GN=DVS81_14735 PE=4 SV=1
A0A011NBY6	155.03	14	4.01E7	1	33234	Poly-beta-hydroxybutyrate polymerase OS=Candidatus Accumulibacter sp. SK-11 OX=1454000 GN=phbC_1 PE=4 SV=1
A0A080LXH2	154.35	23	0.00E+00	1	21642	NADH-dependent phenylglyoxylate dehydrogenase subunit gamma OS=Candidatus Accumulibacter sp. BA-91 OX=1454002 GN=padE PE=4 SV=1
A0A080M413	154.35	23	0.00E+00	1	21715	NADH-dependent phenylglyoxylate dehydrogenase subunit gamma OS=Candidatus Accumulibacter sp. SK-02 OX=1453999 GN=padE PE=4 SV=1
A0A011PIA6	153.89	20	0.00E+00	1	43286	Tryptophan synthase beta chain OS=Candidatus Accumulibacter sp. SK-11 OX=1454000 GN=trpB_1 PE=3 SV=1
A0A351BD82	153.89	20	0.00E+00	1	43531	Tryptophan synthase subunit beta OS=Candidatus Accumulibacter sp. OX=2053492 GN=trpB PE=4 SV=1
A0A011P6S5	153.80	15	0.00E+00	1	44931	Glutamate-1-semialdehyde 2 1-aminomutase OS=Candidatus Accumulibacter sp. BA-92 OX=1454003 GN=hemL PE=3 SV=1
A0A011RIA5	153.77	22	0.00E+00	1	57252	Lysine--tRNA ligase OS=Candidatus Accumulibacter sp. BA-93 OX=1454004 GN=lysS PE=3 SV=1
A0A011QNY8	153.77	22	0.00E+00	1	57425	Lysine--tRNA ligase OS=Candidatus Accumulibacter sp. BA-92 OX=1454003 GN=lysS PE=3 SV=1
A0A080LSC8	152.47	31	0.00E+00	1	17345	Single-stranded DNA-binding protein OS=Candidatus Accumulibacter sp. BA-91 OX=1454002 GN=ssb PE=3 SV=1
A0A011P5W5	152.44	12	0.00E+00	1	40999	Acetyl-CoA acetyltransferase OS=Candidatus Accumulibacter sp. BA-94 OX=1454005 GN=thlA PE=3 SV=1
A0A011PNZ9	151.79	20	1.7E7	1	25463	3-oxoacyl-[acyl-carrier-protein] reductase FabG OS=Candidatus Accumulibacter sp. BA-92 OX=1454003 GN=fabG_5 PE=4 SV=1

A0A369XPS9	151.50	7	0.00E+00	1	76737	Methyl-accepting chemotaxis protein OS=Candidatus Accumulibacter phosphatis OX=327160 GN=DVS81_12645 PE=4 SV=1
A0A080M943	151.45	10	9.79E7	1	58642	Phosphoglucomutase OS=Candidatus Accumulibacter sp. SK-02 OX=1453999 GN=pgm PE=4 SV=1
A0A084Y463	151.45	10	9.79E7	1	58459	Phosphoglucomutase OS=Candidatus Accumulibacter sp. SK-01 OX=1457154 GN=pgm PE=4 SV=1
A0A080MA83	150.96	10	0.00E+00	1	58662	Acetolactate synthase OS=Candidatus Accumulibacter sp. BA-91 OX=1454002 GN=ilvB PE=3 SV=1
A0A011PUJ5	149.98	14	0.00E+00	1	44512	Glutamate-1-semialdehyde 2 1-aminomutase OS=Candidatus Accumulibacter sp. BA-93 OX=1454004 GN=hemL PE=3 SV=1
A0A369XL10	148.94	26	0.00E+00	1	22903	50S ribosomal protein L4 OS=Candidatus Accumulibacter phosphatis OX=327160 GN=DVS81_09315 PE=4 SV=1
A0A011PP98	148.27	25	1.95E7	1	27589	30S ribosomal protein S2 OS=Candidatus Accumulibacter sp. BA-93 OX=1454004 GN=rpsB PE=3 SV=1
A0A011PZ52	148.08	21	0.00E+00	1	44365	Capsular glucan synthase OS=Candidatus Accumulibacter sp. BA-92 OX=1454003 GN=glgA_2 PE=4 SV=1
A0A080LUN9	146.35	11	0.00E+00	1	34106	Branched-chain-amino-acid aminotransferase OS=Candidatus Accumulibacter sp. BA-91 OX=1454002 GN=ilvE PE=3 SV=1
A0A080M4L9	145.31	11	1.85E6	1	44586	Gamma-glutamyl phosphate reductase OS=Candidatus Accumulibacter sp. SK-02 OX=1453999 GN=proA PE=3 SV=1
A0A369XYP0	144.77	34	7.27E7	1	17837	Protein-export chaperone SecB OS=Candidatus Accumulibacter phosphatis OX=327160 GN=DVS81_01560 PE=4 SV=1
A0A011QGV2	144.77	34	7.27E7	1	17934	Protein-export protein SecB OS=Candidatus Accumulibacter sp. BA-92 OX=1454003 GN=secB PE=3 SV=1
A0A1Q3VSH6	144.66	11	4.97E6	1	41715	S-adenosylmethionine synthase OS=Candidatus Accumulibacter sp. 66-26 OX=1895689 GN=metK PE=3 SV=1
A0A011RD97	144.29	31	8.37E6	1	20092	Protein GrpE OS=Candidatus Accumulibacter sp. BA-93 OX=1454004 GN=grpE PE=3 SV=1
A0A011QVW2	143.83	9	1.53E7	1	45813	Putative cobalt-precorrin-6Y C(15)-methyltransferase [decarboxylating] OS=Candidatus Accumulibacter sp. BA-92 OX=1454003 GN=cbiT PE=4 SV=1
A0A080MKL0	143.45	17	0.00E+00	1	44584	Cysteine desulfurase IscS OS=Candidatus Accumulibacter sp. SK-02 OX=1453999 GN=iscS PE=3 SV=1
A0A011NI90	142.97	11	1.29E6	1	97298	DNA gyrase subunit A OS=Candidatus Accumulibacter sp. BA-92 OX=1454003 GN=gyrA PE=3 SV=1
A0A011NJR9	142.72	12	6.2E6	1	34390	Cobalt-precorrin-3B C(17)-methyltransferase OS=Candidatus Accumulibacter sp. BA-92 OX=1454003 GN=cbiH PE=4 SV=1
A0A011PJL0	142.65	15	2.15E6	1	71044	Acetate CoA-transferase YdiF OS=Candidatus Accumulibacter sp. BA-92 OX=1454003 GN=ydiF_2 PE=4 SV=1
A0A369XYG8	142.50	12	0.00E+00	1	50457	Phosphate acetyltransferase OS=Candidatus Accumulibacter phosphatis OX=327160 GN=DVS81_01140 PE=4 SV=1
C7RLS0	142.02	12	0.00E+00	1	27585	Toluene tolerance family protein OS=Accumulibacter phosphatis (strain UW-1) OX=522306 GN=CAP2UW1_0617 PE=4 SV=1
A0A011PMV1	141.94	12	0.00E+00	1	95559	Chaperone protein ClpB OS=Candidatus Accumulibacter sp. BA-92 OX=1454003 GN=clpB PE=3 SV=1
A0A369XLV6	141.74	10	0.00E+00	1	95843	ATP-dependent chaperone ClpB OS=Candidatus Accumulibacter phosphatis OX=327160 GN=clpB PE=4 SV=1
A0A011QXZ3	140.92	29	0.00E+00	1	47051	Transcription termination factor Rho OS=Candidatus Accumulibacter sp. BA-94 OX=1454005 GN=rho PE=3 SV=1

A0A369XKN3	140.09	12	5.61E7	1	49664	Trigger factor OS=Candidatus Accumulibacter phosphatis OX=327160 GN=DVS81_10200 PE=4 SV=1
A0A011RIK4	139.98	17	0.00E+00	1	46227	UDP-N-acetyl-D-glucosamine 6-dehydrogenase OS=Candidatus Accumulibacter sp. BA-93 OX=1454004 GN=wbpA PE=3 SV=1
A0A011NY39	138.58	22	0.00E+00	1	36003	Biotin synthase OS=Candidatus Accumulibacter sp. SK-12 OX=1454001 GN=bioB PE=3 SV=1
A0A011RC43	138.05	19	0.00E+00	1	54543	Transcription termination/antitermination protein NusA OS=Candidatus Accumulibacter sp. BA-93 OX=1454004 GN=nusA PE=3 SV=1
A0A369XKV4	137.62	11	1.83E7	1	34076	Quinone oxidoreductase OS=Candidatus Accumulibacter phosphatis OX=327160 GN=DVS81_16095 PE=4 SV=1
C7RJH8	136.21	17	0.00E+00	1	25797	Carboxylesterase OS=Accumulibacter phosphatis (strain UW-1) OX=522306 GN=CAP2UW1_0183 PE=4 SV=1
A0A011R826	136.16	28	4.34E6	1	17138	Uncharacterized protein OS=Candidatus Accumulibacter sp. BA-93 OX=1454004 GN=AW11_02642 PE=4 SV=1
A0A369XSV1	135.72	20	6.94E5	1	28444	Enoyl-CoA hydratase OS=Candidatus Accumulibacter phosphatis OX=327160 GN=DVS81_06135 PE=4 SV=1
C7RK30	133.74	9	0.00E+00	1	66227	Dihydroxy-acid dehydratase OS=Accumulibacter phosphatis (strain UW-1) OX=522306 GN=ilvD PE=3 SV=1
A0A1A8XEB1	132.93	6	0.00E+00	1	81503	Uncharacterized protein OS=Candidatus Accumulibacter aalborgensis OX=1860102 GN=ACCAA_1050016 PE=4 SV=1
A0A011PF83	132.63	20	0.00E+00	1	30114	Enoyl-[acyl-carrier-protein] reductase [NADH] OS=Candidatus Accumulibacter sp. BA-93 OX=1454004 GN=fabI_1 PE=3 SV=1
A0A011NNW6	132.32	20	0.00E+00	1	26393	1-(5-phosphoribosyl)-5-[(5-phosphoribosylamino)methylideneamino] imidazole-4-carboxamide isomerase OS=Candidatus Accumulibacter sp. SK-12 OX=1454001 GN=hisA PE=3 SV=1
A0A369XZ19	131.45	29	0.00E+00	1	13250	VOC family protein OS=Candidatus Accumulibacter phosphatis OX=327160 GN=DVS81_01675 PE=4 SV=1
A0A011PNY1	130.81	22	0.00E+00	1	18894	Transcription elongation factor GreA OS=Candidatus Accumulibacter sp. SK-12 OX=1454001 GN=greA PE=3 SV=1
A0A011PRA9	129.63	52	4.96E6	1	11793	Glutaredoxin OS=Candidatus Accumulibacter sp. BA-92 OX=1454003 GN=grxD PE=3 SV=1
A0A011NF06	127.88	6	0.00E+00	1	106020	Vitamin B12-dependent ribonucleotide reductase OS=Candidatus Accumulibacter sp. BA-92 OX=1454003 GN=nrdZ PE=3 SV=1
A0A1Q3VS01	127.49	15	0.00E+00	1	39765	ABC transporter substrate-binding protein OS=Candidatus Accumulibacter sp. 66-26 OX=1895689 GN=BGO63_10860 PE=3 SV=1
A0A080LWF7	125.85	24	0.00E+00	1	27412	30S ribosomal protein S2 OS=Candidatus Accumulibacter sp. BA-91 OX=1454002 GN=rpsB PE=3 SV=1
A0A1Q3VQD4	125.21	16	0.00E+00	1	45568	Argininosuccinate synthase OS=Candidatus Accumulibacter sp. 66-26 OX=1895689 GN=argG PE=3 SV=1
A0A369XPL1	124.99	9	0.00E+00	1	95556	DNA topoisomerase III OS=Candidatus Accumulibacter phosphatis OX=327160 GN=DVS81_06490 PE=4 SV=1
A0A011Q9W5	124.90	10	0.00E+00	1	44136	Gamma-glutamyl phosphate reductase OS=Candidatus Accumulibacter sp. BA-93 OX=1454004 GN=proA PE=3 SV=1
A0A1A8XJC5	123.81	13	0.00E+00	1	52484	Uncharacterized protein OS=Candidatus Accumulibacter aalborgensis OX=1860102 GN=ACCAA_180064 PE=3 SV=1

A0A1Q3VX07	123.61	19	0.00E+00	1	23162	NADH-quinone oxidoreductase subunit C OS=Candidatus Accumulibacter sp. 66-26 OX=1895689 GN=nuoC PE=3 SV=1
A0A011QNA3	122.87	7	0.00E+00	1	67614	Chaperone protein HscA homolog OS=Candidatus Accumulibacter sp. BA-93 OX=1454004 GN=hscA_1 PE=3 SV=1
C7RS12	122.36	7	0.00E+00	1	112679	Bifunctional protein PutA OS=Accumulibacter phosphatis (strain UW-1) OX=522306 GN=CAP2UW1_2548 PE=3 SV=1
A0A084Y6U0	120.74	16	0.00E+00	1	29155	Orotidine 5'-phosphate decarboxylase OS=Candidatus Accumulibacter sp. BA-91 OX=1454002 GN=pyrF PE=3 SV=1
A0A1A8XUL9	120.12	17	8.27E7	1	22986	PEBP family protein OS=Candidatus Accumulibacter aalborgensis OX=1860102 GN=ACCAA_630001 PE=4 SV=1
A0A080M7E6	120.05	17	0.00E+00	1	23155	Putative kinase inhibitor protein OS=Candidatus Accumulibacter sp. SK-02 OX=1453999 GN=AW06_001719 PE=4 SV=1
A0A011PV74	120.05	17	0.00E+00	1	22754	Putative kinase inhibitor protein OS=Candidatus Accumulibacter sp. BA-92 OX=1454003 GN=AW10_01642 PE=4 SV=1
A0A369XWF2	119.23	42	0.00E+00	1	12411	Iron-sulfur cluster insertion protein ErpA OS=Candidatus Accumulibacter phosphatis OX=327160 GN=DVS81_04870 PE=4 SV=1
A0A1Q3VSE6	118.96	25	0.00E+00	1	15524	Nucleoside diphosphate kinase OS=Candidatus Accumulibacter sp. 66-26 OX=1895689 GN=ndk PE=3 SV=1
A0A369XHU3	118.90	18	0.00E+00	1	17184	Uncharacterized protein OS=Candidatus Accumulibacter phosphatis OX=327160 GN=DVS81_19555 PE=4 SV=1
A0A011QIN6	118.87	9	0.00E+00	1	40504	Chaperone protein DnaJ OS=Candidatus Accumulibacter sp. BA-93 OX=1454004 GN=dnaJ_1 PE=3 SV=1
A0A011PJP2	117.14	14	0.00E+00	1	46991	Homoserine dehydrogenase OS=Candidatus Accumulibacter sp. BA-92 OX=1454003 GN=hom PE=3 SV=1
A0A011QJ79	116.69	8	0.00E+00	1	52073	Glutamyl-tRNA(Gln) amidotransferase subunit A OS=Candidatus Accumulibacter sp. BA-93 OX=1454004 GN=gatA PE=3 SV=1
A0A011QLY4	116.19	7	0.00E+00	1	49115	Chaperone SurA OS=Candidatus Accumulibacter sp. BA-94 OX=1454005 GN=surA PE=3 SV=1
A0A369XPG1	116.07	20	4.94E6	1	18639	Gamma carbonic anhydrase family protein OS=Candidatus Accumulibacter phosphatis OX=327160 GN=DVS81_04380 PE=4 SV=1
A0A011Q3M6	115.16	23	0.00E+00	1	30055	Bifunctional protein FolD OS=Candidatus Accumulibacter sp. BA-93 OX=1454004 GN=folD PE=3 SV=1
A0A011Q1B7	114.99	21	2.42E6	1	21725	Cob(I)yrinic acid a c-diamide adenosyltransferase OS=Candidatus Accumulibacter sp. BA-92 OX=1454003 GN=btuR PE=3 SV=1
A0A011QH80	114.30	5	2.13E6	1	65845	DNA topoisomerase 3 OS=Candidatus Accumulibacter sp. BA-94 OX=1454005 GN=topB_1 PE=4 SV=1
A0A369XID6	113.80	12	0.00E+00	1	55874	Bifunctional phosphoribosylaminoimidazolecarboxamide formyltransferase/IMP cyclohydrolase PurH OS=Candidatus Accumulibacter phosphatis OX=327160 GN=DVS81_18365 PE=4 SV=1
A0A011QRM6	113.13	14	0.00E+00	1	25089	NADP-dependent malic enzyme OS=Candidatus Accumulibacter sp. BA-94 OX=1454005 GN=maeB_2 PE=4 SV=1
A0A011PQ80	113.05	20	1.55E7	1	17463	Thiol peroxidase OS=Candidatus Accumulibacter sp. BA-92 OX=1454003 GN=tpx PE=3 SV=1
A0A080M9P3	112.83	12	1.35E7	1	21366	Superoxide dismutase OS=Candidatus Accumulibacter sp. SK-02 OX=1453999 GN=sodB PE=3 SV=1
A0A080LTY3	112.83	12	1.35E7	1	21410	Superoxide dismutase OS=Candidatus Accumulibacter sp. BA-91 OX=1454002 GN=sodB PE=3 SV=1

A0A011QYR2	112.41	17	2.73E7	1	21647	Cob(I)yrinic acid a c-diamide adenosyltransferase OS=Candidatus Accumulibacter sp. BA-93 OX=1454004 GN=btuR PE=3 SV=1
A0A011PV55	112.40	16	0.00E+00	1	25400	Uncharacterized protein OS=Candidatus Accumulibacter sp. BA-92 OX=1454003 GN=AW10_01525 PE=4 SV=1
A0A011NKH9	111.56	7	0.00E+00	1	76676	Uncharacterized protein OS=Candidatus Accumulibacter sp. BA-92 OX=1454003 GN=AW10_00019 PE=4 SV=1
A0A011NPI2	111.47	10	0.00E+00	1	46560	Adenylosuccinate synthetase OS=Candidatus Accumulibacter sp. SK-12 OX=1454001 GN=purA PE=3 SV=1
A0A369XKZ6	111.27	16	1.98E7	1	20342	Translation initiation factor IF-3 OS=Candidatus Accumulibacter phosphatis OX=327160 GN=DVS81_10580 PE=4 SV=1
A0A011NL27	110.70	15	5.01E6	1	38763	dTDP-glucose 4 6-dehydratase OS=Candidatus Accumulibacter sp. SK-11 OX=1454000 GN=rfbB_1 PE=4 SV=1
C7RS81	110.19	8	1.56E7	1	34175	Cobalamin (Vitamin B12) biosynthesis CbiX protein OS=Accumulibacter phosphatis (strain UW-1) OX=522306 GN=CAP2UW1_2618 PE=4 SV=1
A0A369XQN3	110.17	14	0.00E+00	1	38407	Uncharacterized protein OS=Candidatus Accumulibacter phosphatis OX=327160 GN=DVS81_07415 PE=4 SV=1
A0A011NU53	109.92	37	1.26E8	1	6319	50S ribosomal protein L32 OS=Candidatus Accumulibacter sp. BA-93 OX=1454004 GN=rpmF PE=3 SV=1
A0A011PYY8	109.92	37	1.26E8	1	6349	50S ribosomal protein L32 OS=Candidatus Accumulibacter sp. BA-92 OX=1454003 GN=rpmF PE=3 SV=1
A0A011PZD6	107.69	11	0.00E+00	1	33779	1 6-anhydro-N-acetylmuramyl-L-alanine amidase AmpD OS=Candidatus Accumulibacter sp. BA-92 OX=1454003 GN=ampD_1 PE=4 SV=1
A0A011R8Z4	107.42	23	2.49E7	1	10574	Uncharacterized protein OS=Candidatus Accumulibacter sp. BA-93 OX=1454004 GN=AW11_02478 PE=4 SV=1
A0A080M869	107.38	21	1.9E7	1	23637	Adenylate kinase OS=Candidatus Accumulibacter sp. SK-02 OX=1453999 GN=adk PE=3 SV=1
A0A080M8U5	107.38	21	1.9E7	1	23779	Adenylate kinase OS=Candidatus Accumulibacter sp. BA-91 OX=1454002 GN=adk PE=3 SV=1
A0A369XIS7	107.20	24	0.00E+00	1	19147	F0F1 ATP synthase subunit delta OS=Candidatus Accumulibacter phosphatis OX=327160 GN=DVS81_13740 PE=4 SV=1
A0A011P7U3	107.19	15	0.00E+00	1	35580	Ornithine carbamoyltransferase OS=Candidatus Accumulibacter sp. BA-93 OX=1454004 GN=argF PE=3 SV=1
A0A011PMM0	106.68	9	0.00E+00	1	57039	NADH-quinone oxidoreductase chain 3 OS=Candidatus Accumulibacter sp. BA-94 OX=1454005 GN=nqo3_1 PE=3 SV=1
A0A011N649	106.52	7	9.51E5	1	43494	Arginine biosynthesis bifunctional protein ArgJ OS=Candidatus Accumulibacter sp. BA-92 OX=1454003 GN=argJ PE=3 SV=1
A0A369XVL3	106.16	7	0.00E+00	1	48317	Transporter OS=Candidatus Accumulibacter phosphatis OX=327160 GN=DVS81_05675 PE=4 SV=1
A0A011QHK1	106.16	8	0.00E+00	1	47909	47 kDa outer membrane protein OS=Candidatus Accumulibacter sp. BA-93 OX=1454004 GN=AW11_02110 PE=4 SV=1
A0A011QSF3	105.95	9	0.00E+00	1	51238	Protease TldD OS=Candidatus Accumulibacter sp. BA-94 OX=1454005 GN=AW12_00729 PE=4 SV=1
A0A346T2R7	105.70	8	0.00E+00	1	41536	Polyphosphate kinase 1 (Fragment) OS=Candidatus Accumulibacter phosphatis OX=327160 GN=ppk1 PE=4 SV=1
A0A1Q3VWW3	105.58	6	0.00E+00	1	82943	NADH-quinone oxidoreductase OS=Candidatus Accumulibacter sp. 66-26 OX=1895689 GN=BGO63_13460 PE=3 SV=1

C7RP75	104.88	6	0.00E+00	1	106501	Valine--tRNA ligase OS=Accumulibacter phosphatis (strain UW-1) OX=522306 GN=valS PE=3 SV=1
C7RVI4	103.79	27	0.00E+00	1	15080	MaoC domain protein dehydratase OS=Accumulibacter phosphatis (strain UW-1) OX=522306 GN=CAP2UW1_3187 PE=4 SV=1
A0A011PQR6	103.72	22	1.4E7	1	20228	Protein GrpE OS=Candidatus Accumulibacter sp. BA-92 OX=1454003 GN=grpE PE=3 SV=1
A0A1A8XPQ2	103.23	7	0.00E+00	1	40708	Aspartate-semialdehyde dehydrogenase OS=Candidatus Accumulibacter aalborgensis OX=1860102 GN=asd PE=3 SV=1
A0A369XI83	103.10	13	3.32E6	1	26505	SDR family NAD(P)-dependent oxidoreductase OS=Candidatus Accumulibacter phosphatis OX=327160 GN=DVS81_14420 PE=4 SV=1
A0A011PAL2	102.30	18	0.00E+00	1	9813	Flavinator of succinate dehydrogenase OS=Candidatus Accumulibacter sp. BA-93 OX=1454004 GN=AW11_03786 PE=4 SV=1
C7RM74	101.49	4	1.67E7	1	68154	TonB-dependent receptor OS=Accumulibacter phosphatis (strain UW-1) OX=522306 GN=CAP2UW1_3902 PE=3 SV=1
C7RPY0	100.84	52	0.00E+00	1	10511	10 kDa chaperonin OS=Accumulibacter phosphatis (strain UW-1) OX=522306 GN=groS PE=3 SV=1
C7RKE7	99.52	14	0.00E+00	1	23555	Adenylate kinase OS=Accumulibacter phosphatis (strain UW-1) OX=522306 GN=adk PE=3 SV=1
A0A011NY90	98.78	11	0.00E+00	1	37806	dTDP-glucose 4 6-dehydratase OS=Candidatus Accumulibacter sp. SK-12 OX=1454001 GN=rfbB_2 PE=4 SV=1
A0A1Q3VSJ8	97.60	8	0.00E+00	1	37354	C4-dicarboxylate ABC transporter OS=Candidatus Accumulibacter sp. 66-26 OX=1895689 GN=BGO63_17705 PE=4 SV=1
C7RN33	97.24	8	0.00E+00	1	37114	TRAP dicarboxylate transporter DctP subunit OS=Accumulibacter phosphatis (strain UW-1) OX=522306 GN=CAP2UW1_2104 PE=4 SV=1
A0A011PVT1	97.11	6	0.00E+00	1	54828	Uncharacterized protein OS=Candidatus Accumulibacter sp. BA-92 OX=1454003 GN=AW10_01559 PE=4 SV=1
A0A011NVZ1	96.27	8	0.00E+00	1	49246	Tryptophan synthase beta chain OS=Candidatus Accumulibacter sp. BA-92 OX=1454003 GN=trpB_2 PE=3 SV=1
A0A011N6A4	96.06	15	0.00E+00	1	14686	50S ribosomal protein L17 OS=Candidatus Accumulibacter sp. BA-92 OX=1454003 GN=rplQ PE=3 SV=1
A0A369XPK5	96.06	15	0.00E+00	1	14630	50S ribosomal protein L17 OS=Candidatus Accumulibacter phosphatis OX=327160 GN=DVS81_09185 PE=4 SV=1
A0A011NQ76	96.06	15	0.00E+00	1	14791	50S ribosomal protein L17 OS=Candidatus Accumulibacter sp. SK-12 OX=1454001 GN=rplQ PE=3 SV=1
A0A011PK59	96.06	15	0.00E+00	1	14816	50S ribosomal protein L17 OS=Candidatus Accumulibacter sp. BA-94 OX=1454005 GN=rplQ PE=3 SV=1
A0A1A8XXF8	96.06	14	0.00E+00	1	14771	50S ribosomal protein L17 OS=Candidatus Accumulibacter aalborgensis OX=1860102 GN=rplQ PE=3 SV=1
A0A369XV80	95.93	34	0.00E+00	1	9357	Acyl-CoA-binding protein OS=Candidatus Accumulibacter phosphatis OX=327160 GN=DVS81_07020 PE=4 SV=1
A0A080LZG9	95.91	5	0.00E+00	1	68314	Outer membrane cobalamin translocator OS=Candidatus Accumulibacter sp. BA-91 OX=1454002 GN=btuB PE=3 SV=1
A0A084XY80	95.13	26	0.00E+00	1	17610	Glutathione peroxidase OS=Candidatus Accumulibacter sp. SK-01 OX=1457154 GN=CAPSK01_003184 PE=3 SV=1
A0A369XM79	93.90	11	0.00E+00	1	43215	MaoC family dehydratase OS=Candidatus Accumulibacter phosphatis OX=327160 GN=DVS81_08415 PE=4 SV=1

A0A011P1D5	93.48	13	0.00E+00	1	35284	Asparagine synthetase [glutamine-hydrolyzing] 1 OS=Candidatus Accumulibacter sp. BA-92 OX=1454003 GN=asnB_4 PE=4 SV=1
A0A369XNB2	92.26	22	0.00E+00	1	10347	Uncharacterized protein OS=Candidatus Accumulibacter phosphatis OX=327160 GN=DVS81_14280 PE=4 SV=1
A0A1A8XHU2	91.63	6	0.00E+00	1	49582	Outer membrane protein transport protein (OMPP1/FadL/TodX) OS=Candidatus Accumulibacter aalborgensis OX=1860102 GN=ACCAA_1050015 PE=4 SV=1
A0A011PP18	90.27	10	0.00E+00	1	40798	Ferrochelatase OS=Candidatus Accumulibacter sp. BA-93 OX=1454004 GN=hemH PE=3 SV=1
A0A011NHU7	89.87	10	0.00E+00	1	43607	Acriflavine resistance protein A OS=Candidatus Accumulibacter sp. BA-92 OX=1454003 GN=acrA PE=3 SV=1
A0A084XXC7	89.59	5	0.00E+00	1	55324	NAD(P) transhydrogenase subunit alpha OS=Candidatus Accumulibacter sp. SK-01 OX=1457154 GN=pntA PE=3 SV=1
A0A369XJ50	88.90	9	0.00E+00	1	50217	Phosphomannomutase/phosphoglucomutase OS=Candidatus Accumulibacter phosphatis OX=327160 GN=DVS81_12885 PE=4 SV=1
A0A011PQS1	88.38	9	0.00E+00	1	19977	Uncharacterized protein OS=Candidatus Accumulibacter sp. BA-93 OX=1454004 GN=AW11_01266 PE=4 SV=1
A0A369XMH8	88.32	8	0.00E+00	1	41000	Quinolinate synthase NadA OS=Candidatus Accumulibacter phosphatis OX=327160 GN=DVS81_07455 PE=4 SV=1
A0A1A8XZJ5	88.32	8	0.00E+00	1	41102	Quinolinate synthase A OS=Candidatus Accumulibacter aalborgensis OX=1860102 GN=nadA PE=3 SV=1
A0A011QVQ6	88.32	8	0.00E+00	1	40853	Quinolinate synthase A OS=Candidatus Accumulibacter sp. BA-92 OX=1454003 GN=nadA PE=3 SV=1
A0A1Q3VRM0	88.32	8	0.00E+00	1	40941	Quinolinate synthase A OS=Candidatus Accumulibacter sp. 66-26 OX=1895689 GN=nadA PE=3 SV=1
A0A080MAH5	88.24	37	2.9E6	1	7277	50S ribosomal protein L29 OS=Candidatus Accumulibacter sp. SK-02 OX=1453999 GN=rpmC PE=3 SV=1
A0A080LUV3	88.24	36	2.9E6	1	7386	50S ribosomal protein L29 OS=Candidatus Accumulibacter sp. BA-91 OX=1454002 GN=rpmC PE=3 SV=1
A0A084Y3A3	88.24	36	2.9E6	1	7405	50S ribosomal protein L29 OS=Candidatus Accumulibacter sp. SK-01 OX=1457154 GN=rpmC PE=3 SV=1
A0A369XLK6	88.24	36	2.9E6	1	7405	50S ribosomal protein L29 OS=Candidatus Accumulibacter phosphatis OX=327160 GN=DVS81_09280 PE=4 SV=1
A0A011P7B1	88.24	36	2.9E6	1	7405	50S ribosomal protein L29 OS=Candidatus Accumulibacter sp. BA-93 OX=1454004 GN=rpmC PE=3 SV=1
A0A011N5I7	87.02	18	0.00E+00	1	22939	ATP phosphoribosyltransferase OS=Candidatus Accumulibacter sp. BA-92 OX=1454003 GN=hisG PE=3 SV=1
A0A011PNU5	86.85	6	2.56E7	1	22218	Uncharacterized protein OS=Candidatus Accumulibacter sp. BA-93 OX=1454004 GN=AW11_01765 PE=4 SV=1
A0A369XIL4	86.61	12	0.00E+00	1	34203	Oxidoreductase OS=Candidatus Accumulibacter phosphatis OX=327160 GN=DVS81_17545 PE=4 SV=1
A0A011PPT7	86.61	12	0.00E+00	1	34168	Acrylyl-CoA reductase AcuI OS=Candidatus Accumulibacter sp. BA-92 OX=1454003 GN=acuI_2 PE=4 SV=1
A0A011NXF2	86.10	6	0.00E+00	1	92578	Cyanophycin synthetase OS=Candidatus Accumulibacter sp. SK-12 OX=1454001 GN=cphA_2 PE=3 SV=1
A0A011Q7U7	85.97	27	7.25E6	1	5392	Uncharacterized protein OS=Candidatus Accumulibacter sp. BA-93 OX=1454004 GN=AW11_03635 PE=4 SV=1
A0A011PH72	85.71	7	0.00E+00	1	55876	Methylmalonyl-CoA carboxyltransferase 12S subunit OS=Candidatus Accumulibacter sp. SK-12 OX=1454001 GN=AW08_03129 PE=4 SV=1
A0A011P6T1	84.96	34	0.00E+00	1	13037	Stalked cell differentiation-controlling protein OS=Candidatus Accumulibacter sp. BA-92 OX=1454003 GN=pleD_1 PE=4 SV=1

A0A011PUJ1	84.96	34	0.00E+00	1	13037	Stalked cell differentiation-controlling protein OS=Candidatus Accumulibacter sp. BA-93 OX=1454004 GN=pleD_3 PE=4 SV=1
A0A369XP47	84.96	34	0.00E+00	1	13009	Response regulator OS=Candidatus Accumulibacter phosphatis OX=327160 GN=DVS81_12655 PE=4 SV=1
A0A080LVB9	84.96	34	0.00E+00	1	13093	Stalked cell differentiation-controlling protein OS=Candidatus Accumulibacter sp. BA-91 OX=1454002 GN=pleD_6 PE=4 SV=1
A0A011NSM5	84.73	4	0.00E+00	1	39696	3-dehydroquinate synthase OS=Candidatus Accumulibacter sp. BA-93 OX=1454004 GN=aroB PE=3 SV=1
A0A011QPE1	84.60	9	5.59E7	1	17288	Superoxide dismutase [Cu-Zn] OS=Candidatus Accumulibacter sp. BA-93 OX=1454004 GN=sodC1 PE=3 SV=1
A0A369XQH4	84.36	15	0.00E+00	1	43883	2-methylfumaryl-CoA isomerase OS=Candidatus Accumulibacter phosphatis OX=327160 GN=DVS81_08440 PE=4 SV=1
A0A011QP11	83.01	15	6.47E6	1	14702	50S ribosomal protein L17 OS=Candidatus Accumulibacter sp. BA-93 OX=1454004 GN=rplQ PE=3 SV=1
A0A011PN62	82.63	16	3.9E6	1	13954	Ribosomal silencing factor RsfS OS=Candidatus Accumulibacter sp. BA-93 OX=1454004 GN=rsfS PE=3 SV=1
A0A080M6U8	81.92	4	0.00E+00	1	72558	Potassium-transporting ATPase ATP-binding subunit OS=Candidatus Accumulibacter sp. SK-02 OX=1453999 GN=kdpB PE=3 SV=1
A0A011PAC5	81.92	4	0.00E+00	1	60076	Potassium-transporting ATPase ATP-binding subunit OS=Candidatus Accumulibacter sp. SK-11 OX=1454000 GN=kdpB PE=3 SV=1
A0A351BDJ3	81.92	4	0.00E+00	1	66732	K(+)-transporting ATPase subunit B (Fragment) OS=Candidatus Accumulibacter sp. OX=2053492 GN=kdpB PE=4 SV=1
C7RUE5	81.92	4	0.00E+00	1	71823	Potassium-transporting ATPase ATP-binding subunit OS=Accumulibacter phosphatis (strain UW-1) OX=522306 GN=kdpB PE=3 SV=1
A0A011PIF7	81.92	4	0.00E+00	1	71772	Potassium-transporting ATPase ATP-binding subunit OS=Candidatus Accumulibacter sp. SK-12 OX=1454001 GN=kdpB PE=3 SV=1
A0A1A8XWD5	81.68	20	4.24E7	1	15933	Heat shock protein Hsp20 OS=Candidatus Accumulibacter aalborgensis OX=1860102 GN=ACCAA_680037 PE=3 SV=1
A0A011PPQ2	80.76	10	8.31E6	1	24236	Fumarylpyruvate hydrolase OS=Candidatus Accumulibacter sp. BA-93 OX=1454004 GN=nagK PE=4 SV=1
A0A011QGT9	80.69	17	0.00E+00	1	16913	Uncharacterized protein OS=Candidatus Accumulibacter sp. BA-92 OX=1454003 GN=AW10_03312 PE=4 SV=1
A0A011RDM7	80.66	6	0.00E+00	1	67499	Protein translocase subunit SecD OS=Candidatus Accumulibacter sp. BA-93 OX=1454004 GN=secD PE=3 SV=1
A0A011PXI7	80.58	13	0.00E+00	1	36295	Phosphoribosylformylglycinamidine cyclo-ligase OS=Candidatus Accumulibacter sp. BA-92 OX=1454003 GN=purM PE=3 SV=1
A0A011QGR6	80.54	4	1.71E6	1	34483	HTH-type transcriptional repressor YcgE OS=Candidatus Accumulibacter sp. BA-93 OX=1454004 GN=ycgE_1 PE=4 SV=1
A0A011QW34	80.01	5	3.68E6	1	72642	Hsc66 OS=Candidatus Accumulibacter sp. BA-94 OX=1454005 GN=hscA PE=3 SV=1
A0A369XN19	79.70	23	8.38E6	1	6707	50S ribosomal protein L30 OS=Candidatus Accumulibacter phosphatis OX=327160 GN=DVS81_09230 PE=4 SV=1

A0A011P7A2	79.70	23	8.38E6	1	6607	50S ribosomal protein L30 OS=Candidatus Accumulibacter sp. BA-93 OX=1454004 GN=rpmD PE=3 SV=1
A0A011QH21	79.70	20	8.38E6	1	7639	50S ribosomal protein L30 OS=Candidatus Accumulibacter sp. BA-92 OX=1454003 GN=rpmD PE=3 SV=1
A0A1Q3VSU3	78.97	11	6.45E6	1	31318	Elongation factor Ts OS=Candidatus Accumulibacter sp. 66-26 OX=1895689 GN=tsf PE=3 SV=1
A0A084Y395	77.59	21	0.00E+00	1	12940	50S ribosomal protein L18 OS=Candidatus Accumulibacter sp. SK-01 OX=1457154 GN=rplR PE=3 SV=1
A0A011QJP3	77.04	21	4.94E6	1	7305	DNA-directed RNA polymerase subunit omega OS=Candidatus Accumulibacter sp. BA-93 OX=1454004 GN=rpoZ PE=3 SV=1
A0A369XUF1	77.04	21	4.94E6	1	7306	DNA-directed RNA polymerase subunit omega OS=Candidatus Accumulibacter phosphatis OX=327160 GN=DVS81_04120 PE=4 SV=1
A0A080LWN0	76.72	10	0.00E+00	1	20578	Putative kinase inhibitor OS=Candidatus Accumulibacter sp. BA-91 OX=1454002 GN=AW09_001730 PE=4 SV=1
A0A011QGE3	76.40	13	0.00E+00	1	10449	Helix-turn-helix domain protein OS=Candidatus Accumulibacter sp. BA-93 OX=1454004 GN=AW11_02248 PE=4 SV=1
A0A011NY99	76.15	4	0.00E+00	1	48331	Phosphoenolpyruvate carboxykinase [GTP] OS=Candidatus Accumulibacter sp. BA-94 OX=1454005 GN=pckG_2 PE=3 SV=1
A0A011QK27	76.09	8	0.00E+00	1	31113	SapC OS=Candidatus Accumulibacter sp. BA-92 OX=1454003 GN=AW10_02503 PE=4 SV=1
A0A011QMA8	75.98	8	0.00E+00	1	49492	ATP-dependent protease ATPase subunit HslU OS=Candidatus Accumulibacter sp. BA-93 OX=1454004 GN=hslU PE=3 SV=1
A0A011NTP5	75.91	11	0.00E+00	1	45408	Lactate utilization protein A OS=Candidatus Accumulibacter sp. BA-92 OX=1454003 GN=lutA_2 PE=4 SV=1
A0A011QNM4	75.68	8	0.00E+00	1	25021	Haloacetate dehalogenase H-2 OS=Candidatus Accumulibacter sp. BA-93 OX=1454004 GN=dehH2 PE=4 SV=1
S6BAA0	75.45	6	0.00E+00	1	40954	Polyphosphate kinase (Fragment) OS=uncultured Candidatus Accumulibacter sp. OX=759355 GN=ppk1 PE=4 SV=1
A0A011P6U3	75.38	6	0.00E+00	1	22061	Polyhydroxyalkanoate synthesis repressor PhaR OS=Candidatus Accumulibacter sp. BA-93 OX=1454004 GN=AW11_00546 PE=4 SV=1
A0A011PV77	75.38	6	0.00E+00	1	22061	Polyhydroxyalkanoate synthesis repressor PhaR OS=Candidatus Accumulibacter sp. BA-92 OX=1454003 GN=AW10_01545 PE=4 SV=1
A0A011P2C5	75.26	2	0.00E+00	1	104072	Uncharacterized protein OS=Candidatus Accumulibacter sp. BA-93 OX=1454004 GN=AW11_01762 PE=4 SV=1
A0A011PMX3	75.00	20	0.00E+00	1	16906	Uncharacterized protein OS=Candidatus Accumulibacter sp. BA-93 OX=1454004 GN=AW11_01909 PE=4 SV=1
A0A011QDD1	74.16	5	0.00E+00	1	30176	Putative enoyl-CoA hydratase echA8 OS=Candidatus Accumulibacter sp. BA-93 OX=1454004 GN=echA8_5 PE=3 SV=1
A0A011PQ46	74.07	4	0.00E+00	1	34167	Ornithine cyclodeaminase OS=Candidatus Accumulibacter sp. BA-93 OX=1454004 GN=AW11_01423 PE=4 SV=1
A0A011NKJ8	73.96	18	0.00E+00	1	25414	LIV-I protein F OS=Candidatus Accumulibacter sp. BA-92 OX=1454003 GN=livF_1 PE=4 SV=1
A0A369XLC3	73.76	5	2.36E6	1	25980	YebC/PmpR family DNA-binding transcriptional regulator OS=Candidatus Accumulibacter phosphatis OX=327160 GN=DVS81_12700 PE=4 SV=1

A0A011NH85	73.74	9	4.48E6	1	31979	Malonyl CoA-acyl carrier protein transacylase OS=Candidatus Accumulibacter sp. BA-92 OX=1454003 GN=fabD PE=3 SV=1
A0A369XQN5	73.74	9	4.48E6	1	31930	[acyl-carrier-protein] S-malonyltransferase OS=Candidatus Accumulibacter phosphatis OX=327160 GN=fabD PE=4 SV=1
C7RV55	73.72	11	0.00E+00	1	33265	Extracellular solute-binding protein family 3 OS=Accumulibacter phosphatis (strain UW-1) OX=522306 GN=CAP2UW1_1834 PE=4 SV=1
A0A011P359	73.64	17	0.00E+00	1	22835	ATP phosphoribosyltransferase OS=Candidatus Accumulibacter sp. BA-93 OX=1454004 GN=hisG PE=3 SV=1
A0A084Y3S1	73.58	6	0.00E+00	1	50358	Trigger factor OS=Candidatus Accumulibacter sp. SK-01 OX=1457154 GN=tig PE=3 SV=1
A0A011RIP6	73.51	16	0.00E+00	1	25350	LIV-I protein F OS=Candidatus Accumulibacter sp. BA-93 OX=1454004 GN=livF_2 PE=4 SV=1
C7RN43	73.12	8	0.00E+00	1	39967	Outer membrane protein assembly factor BamB OS=Accumulibacter phosphatis (strain UW-1) OX=522306 GN=bamB PE=3 SV=1
A0A1A8XRK8	73.02	8	0.00E+00	1	45580	Serine hydroxymethyltransferase OS=Candidatus Accumulibacter aalborgensis OX=1860102 GN=glyA PE=3 SV=1
A0A369XLE9	72.61	12	2.00E+07	1	12734	Uncharacterized protein OS=Candidatus Accumulibacter phosphatis OX=327160 GN=DVS81_12450 PE=4 SV=1
A0A011PRM9	72.48	3	0.00E+00	1	49648	Transcriptional regulatory protein ZraR OS=Candidatus Accumulibacter sp. BA-92 OX=1454003 GN=zraR_2 PE=4 SV=1
A0A011NZ95	72.48	3	0.00E+00	1	49807	Transcriptional regulatory protein ZraR OS=Candidatus Accumulibacter sp. BA-93 OX=1454004 GN=zraR_3 PE=4 SV=1
A0A369XKA8	72.48	3	0.00E+00	1	49739	Sigma-54-dependent Fis family transcriptional regulator OS=Candidatus Accumulibacter phosphatis OX=327160 GN=DVS81_10310 PE=4 SV=1
A0A351BJG1	72.48	3	0.00E+00	1	50260	Sigma-54-dependent Fis family transcriptional regulator OS=Candidatus Accumulibacter sp. OX=2053492 GN=DCY47_13270 PE=4 SV=1
A0A011NEN5	72.48	3	0.00E+00	1	50260	Transcriptional regulatory protein ZraR OS=Candidatus Accumulibacter sp. SK-11 OX=1454000 GN=zraR_2 PE=4 SV=1
A0A011Q0B2	72.48	3	0.00E+00	1	50125	Transcriptional regulatory protein ZraR OS=Candidatus Accumulibacter sp. BA-94 OX=1454005 GN=zraR_1 PE=4 SV=1
A0A011NUT3	72.48	3	0.00E+00	1	50071	Transcriptional regulatory protein ZraR OS=Candidatus Accumulibacter sp. SK-12 OX=1454001 GN=zraR_2 PE=4 SV=1
C7RT80	72.41	28	0.00E+00	1	17029	Protein-export protein SecB OS=Accumulibacter phosphatis (strain UW-1) OX=522306 GN=secB PE=3 SV=1
A0A084XY53	71.78	7	0.00E+00	1	45397	Glutamate-pyruvate aminotransferase AlaA OS=Candidatus Accumulibacter sp. SK-01 OX=1457154 GN=alaA PE=4 SV=1
A0A1A8XLN2	71.54	9	0.00E+00	1	16677	Putative peroxiredoxin YgaF OS=Candidatus Accumulibacter aalborgensis OX=1860102 GN=ygaF PE=4 SV=1
A0A011R5H8	71.54	9	0.00E+00	1	16802	Putative peroxiredoxin bcp OS=Candidatus Accumulibacter sp. BA-93 OX=1454004 GN=bcp_1 PE=4 SV=1
A0A080LZF7	71.54	9	0.00E+00	1	16756	Putative peroxiredoxin bcp OS=Candidatus Accumulibacter sp. BA-91 OX=1454002 GN=bcp_2 PE=4 SV=1
A0A1Q3VU55	71.54	9	0.00E+00	1	16660	Peroxiredoxin OS=Candidatus Accumulibacter sp. 66-26 OX=1895689 GN=BGO63_17990 PE=4 SV=1

A0A084XZP8	71.54	9	0.00E+00	1	17003	Putative peroxiredoxin bcp OS=Candidatus Accumulibacter sp. SK-01 OX=1457154 GN=bcp_2 PE=4 SV=1
A0A011RJ77	71.00	3	0.00E+00	1	45546	Zinc metallopeptidase RseP OS=Candidatus Accumulibacter sp. BA-93 OX=1454004 GN=AW11_00043 PE=4 SV=1
A0A011PXH9	70.99	9	8.83E6	1	16655	Uncharacterized protein OS=Candidatus Accumulibacter sp. BA-92 OX=1454003 GN=AW10_01092 PE=4 SV=1
A0A011PQ53	70.70	9	0.00E+00	1	13877	Nitrogen regulatory protein P-II OS=Candidatus Accumulibacter sp. BA-93 OX=1454004 GN=AW11_01433 PE=3 SV=1
A0A011RHI9	69.36	12	0.00E+00	1	15224	Uncharacterized protein OS=Candidatus Accumulibacter sp. BA-93 OX=1454004 GN=AW11_00536 PE=4 SV=1
A0A011PTZ4	68.57	13	0.00E+00	1	25575	3-oxoacyl-[acyl-carrier-protein] reductase FabG OS=Candidatus Accumulibacter sp. BA-94 OX=1454005 GN=fabG_2 PE=4 SV=1
A0A011QIY0	68.55	3	4.39E7	1	52930	Uncharacterized protein OS=Candidatus Accumulibacter sp. BA-93 OX=1454004 GN=AW11_01636 PE=3 SV=1
A0A369XS41	68.55	3	4.39E7	1	53004	Cytochrome-c oxidase cbb3-type subunit I OS=Candidatus Accumulibacter phosphatis OX=327160 GN=ccoN PE=4 SV=1
A0A011NUP2	68.40	4	3.68E6	1	35224	Hopanoid-associated sugar epimerase OS=Candidatus Accumulibacter sp. BA-92 OX=1454003 GN=AW10_02698 PE=4 SV=1
C7RSH8	67.37	3	0.00E+00	1	37812	Lytic murein transglycosylase B OS=Accumulibacter phosphatis (strain UW-1) OX=522306 GN=CAP2UW1_1367 PE=4 SV=1
A0A1A8XFZ0	67.26	3	1.00E+07	1	46423	Precorrin-6Y C5 15-methyltransferase (Decarboxylating) CbiT subunit OS=Candidatus Accumulibacter aalborgensis OX=1860102 GN=ACCAA_100007 PE=4 SV=1
C7RS78	67.26	3	1.00E+07	1	45314	Precorrin-6Y C5 15-methyltransferase (Decarboxylating) CbiT subunit OS=Accumulibacter phosphatis (strain UW-1) OX=522306 GN=CAP2UW1_2615 PE=4 SV=1
A0A1Q3VRT9	67.26	4	5.86E6	1	31894	dTDP-4-dehydrorhamnose reductase OS=Candidatus Accumulibacter sp. 66-26 OX=1895689 GN=BGO63_08770 PE=3 SV=1
A0A369XKZ5	67.02	4	0.00E+00	1	69477	Diguanylate cyclase OS=Candidatus Accumulibacter phosphatis OX=327160 GN=DVS81_12980 PE=4 SV=1
A0A011PN35	66.88	5	1.34E6	1	44649	UDP-N-acetylglucosamine 1-carboxyvinyltransferase OS=Candidatus Accumulibacter sp. BA-92 OX=1454003 GN=murA PE=3 SV=1
A0A011PSZ5	66.78	9	2.63E6	1	22998	Carbonic anhydrase OS=Candidatus Accumulibacter sp. BA-93 OX=1454004 GN=icfA PE=3 SV=1
C7RM24	66.30	27	0.00E+00	1	18705	Transferase hexapeptide repeat protein OS=Accumulibacter phosphatis (strain UW-1) OX=522306 GN=CAP2UW1_1977 PE=4 SV=1
A0A369XTP3	65.80	2	0.00E+00	1	64261	Uncharacterized protein OS=Candidatus Accumulibacter phosphatis OX=327160 GN=DVS81_03850 PE=4 SV=1
A0A011PCV9	65.75	11	0.00E+00	1	43531	Polysaccharide export protein Wza OS=Candidatus Accumulibacter sp. BA-93 OX=1454004 GN=AW11_03534 PE=4 SV=1
A0A011QHR8	65.45	3	2.98E6	1	51501	Mannose-1-phosphate guanylyltransferase 1 OS=Candidatus Accumulibacter sp. BA-93 OX=1454004 GN=manC1 PE=3 SV=1
A0A011QG98	65.13	5	0.00E+00	1	46947	Histidinol dehydrogenase OS=Candidatus Accumulibacter sp. BA-92 OX=1454003 GN=hisD PE=3 SV=1
A0A011RDU7	65.13	5	0.00E+00	1	46659	Histidinol dehydrogenase OS=Candidatus Accumulibacter sp. BA-93 OX=1454004 GN=hisD PE=3 SV=1

A0A369XN22	65.13	5	0.00E+00	1	46754	Histidinol dehydrogenase OS=Candidatus Accumulibacter phosphatis OX=327160 GN=hisD PE=4 SV=1
A0A1A8XRB8	65.11	2	1.28E7	1	77598	Cyanophycin synthetase OS=Candidatus Accumulibacter aalborgensis OX=1860102 GN=cphA PE=4 SV=1
A0A011PV71	64.03	2	0.00E+00	1	63973	Gamma-glutamyltranspeptidase OS=Candidatus Accumulibacter sp. BA-93 OX=1454004 GN=ggt PE=4 SV=1
A0A1A8XE84	63.95	10	0.00E+00	1	17917	Outer membrane protein assembly factor BamE OS=Candidatus Accumulibacter aalborgensis OX=1860102 GN=bamE PE=3 SV=1
A0A011P681	63.95	10	0.00E+00	1	17703	Outer membrane protein assembly factor BamE OS=Candidatus Accumulibacter sp. BA-93 OX=1454004 GN=bamE PE=3 SV=1
A0A011NVK4	63.37	15	1.23E7	1	9493	30S ribosomal protein S16 OS=Candidatus Accumulibacter sp. BA-92 OX=1454003 GN=rpsP PE=3 SV=1
A0A369XR01	63.37	15	1.23E7	1	9507	30S ribosomal protein S16 OS=Candidatus Accumulibacter phosphatis OX=327160 GN=DVS81_00655 PE=4 SV=1
A0A011NFW1	63.24	7	8.54E5	1	21450	Alkyl hydroperoxide reductase AhpD OS=Candidatus Accumulibacter sp. BA-92 OX=1454003 GN=AW10_01095 PE=3 SV=1
A0A011QLC6	63.24	8	8.54E5	1	19166	Alkyl hydroperoxide reductase AhpD OS=Candidatus Accumulibacter sp. BA-92 OX=1454003 GN=AW10_02267 PE=3 SV=1
A0A011NY21	63.21	9	0.00E+00	1	13009	Protease production enhancer protein OS=Candidatus Accumulibacter sp. BA-93 OX=1454004 GN=degU PE=4 SV=1
A0A011PX40	63.19	5	0.00E+00	1	26216	Benzil reductase ((S)-benzoin forming) OS=Candidatus Accumulibacter sp. BA-92 OX=1454003 GN=yueD PE=4 SV=1
A0A011Q4J1	63.19	5	0.00E+00	1	24810	Benzil reductase ((S)-benzoin forming) OS=Candidatus Accumulibacter sp. BA-93 OX=1454004 GN=yueD PE=4 SV=1
A0A369XKN6	63.12	2	0.00E+00	1	60255	AMP-binding protein OS=Candidatus Accumulibacter phosphatis OX=327160 GN=DVS81_14310 PE=4 SV=1
A0A011QP24	63.04	1	0.00E+00	1	144268	Phosphoribosylformylglycinamidine synthase OS=Candidatus Accumulibacter sp. BA-92 OX=1454003 GN=purL PE=3 SV=1
A0A011NWC6	62.66	7	0.00E+00	1	18262	Leucine-responsive regulatory protein OS=Candidatus Accumulibacter sp. BA-93 OX=1454004 GN=lrp_2 PE=4 SV=1
A0A011QJB4	62.09	12	0.00E+00	1	35841	UDP-glucose 4-epimerase OS=Candidatus Accumulibacter sp. BA-93 OX=1454004 GN=galE PE=4 SV=1
A0A011Q4Z1	61.54	6	0.00E+00	1	34186	Histone deacetylase-like amidohydrolase OS=Candidatus Accumulibacter sp. BA-93 OX=1454004 GN=hdaH_2 PE=4 SV=1
A0A011PLC7	61.54	6	0.00E+00	1	34249	Histone deacetylase-like amidohydrolase OS=Candidatus Accumulibacter sp. BA-92 OX=1454003 GN=hdaH_3 PE=4 SV=1
A0A011PLN9	61.49	9	0.00E+00	1	49484	ATP-dependent protease ATPase subunit HslU OS=Candidatus Accumulibacter sp. BA-92 OX=1454003 GN=hslU PE=3 SV=1
A0A369XRS6	60.44	8	0.00E+00	1	43652	Sugar transporter OS=Candidatus Accumulibacter phosphatis OX=327160 GN=DVS81_08885 PE=4 SV=1
A0A011RI79	60.34	4	0.00E+00	1	31665	Disulfide-bond oxidoreductase YghU OS=Candidatus Accumulibacter sp. BA-93 OX=1454004 GN=yghU PE=4 SV=1
A0A011PBB8	59.95	8	0.00E+00	1	21258	T(6)A37 threonylcarbamoyladenosine biosynthesis protein RimN OS=Candidatus Accumulibacter sp. BA-94 OX=1454005 GN=rimN PE=3 SV=1

A0A084Y6S0	59.95	7	0.00E+00	1	22709	T(6)A37 threonylcarbamoyladenosine biosynthesis protein RimN OS=Candidatus Accumulibacter sp. BA-91 OX=1454002 GN=rimN_2 PE=3 SV=1
C7RU89	59.95	7	0.00E+00	1	22626	Sua5/YciO/YrdC/YwlC family protein OS=Accumulibacter phosphatis (strain UW-1) OX=522306 GN=CAP2UW1_2975 PE=3 SV=1
A0A080M740	59.95	7	0.00E+00	1	22637	T(6)A37 threonylcarbamoyladenosine biosynthesis protein RimN OS=Candidatus Accumulibacter sp. SK-02 OX=1453999 GN=rimN_1 PE=3 SV=1
A0A011PMW6	59.95	7	0.00E+00	1	22429	tRNA(ANN) t(6)A37 threonylcarbamoyladenosine modification protein OS=Candidatus Accumulibacter sp. BA-93 OX=1454004 GN=AW11_01904 PE=3 SV=1
A0A351BG91	59.95	7	0.00E+00	1	22819	Threonylcarbamoyl-AMP synthase OS=Candidatus Accumulibacter sp. OX=2053492 GN=DCY47_07385 PE=4 SV=1
A0A011PAZ8	59.95	7	0.00E+00	1	22819	T(6)A37 threonylcarbamoyladenosine biosynthesis protein RimN OS=Candidatus Accumulibacter sp. SK-11 OX=1454000 GN=rimN PE=3 SV=1
A0A011NS44	59.95	7	0.00E+00	1	22892	T(6)A37 threonylcarbamoyladenosine biosynthesis protein RimN OS=Candidatus Accumulibacter sp. SK-12 OX=1454001 GN=rimN_2 PE=3 SV=1
A0A011ND71	59.95	7	0.00E+00	1	22578	T(6)A37 threonylcarbamoyladenosine biosynthesis protein RimN OS=Candidatus Accumulibacter sp. BA-92 OX=1454003 GN=rimN_2 PE=3 SV=1
A0A084XZ01	59.95	7	0.00E+00	1	22665	T(6)A37 threonylcarbamoyladenosine biosynthesis protein RimN OS=Candidatus Accumulibacter sp. SK-01 OX=1457154 GN=rimN_2 PE=3 SV=1
A0A369XI02	59.95	7	0.00E+00	1	22629	Threonylcarbamoyl-AMP synthase OS=Candidatus Accumulibacter phosphatis OX=327160 GN=DVS81_18815 PE=4 SV=1
A0A1Q3VWX2	59.48	4	0.00E+00	1	30732	4-hydroxy-tetrahydrodipicolinate synthase OS=Candidatus Accumulibacter sp. 66-26 OX=1895689 GN=dapA PE=3 SV=1
A0A369XLY3	58.99	13	1.1E7	1	17489	DUF4124 domain-containing protein OS=Candidatus Accumulibacter phosphatis OX=327160 GN=DVS81_07440 PE=4 SV=1
A0A011PHS4	58.54	5	2.12E6	1	43628	Membrane-bound lytic murein transglycosylase B OS=Candidatus Accumulibacter sp. BA-93 OX=1454004 GN=mltB_1 PE=4 SV=1
A0A011R8M7	58.36	9	0.00E+00	1	23051	Uncharacterized protein OS=Candidatus Accumulibacter sp. BA-93 OX=1454004 GN=AW11_02608 PE=4 SV=1
A0A011QM91	58.29	3	1.59E6	1	37946	Beta-lactamase hydrolase-like protein OS=Candidatus Accumulibacter sp. BA-92 OX=1454003 GN=blh_1 PE=4 SV=1
A0A011Q881	58.29	3	1.59E6	1	38015	Beta-lactamase hydrolase-like protein OS=Candidatus Accumulibacter sp. BA-93 OX=1454004 GN=blh_1 PE=4 SV=1
A0A369XNJ4	58.29	3	1.59E6	1	37924	MBL fold metallo-hydrolase OS=Candidatus Accumulibacter phosphatis OX=327160 GN=DVS81_08960 PE=4 SV=1
A0A011QQ66	57.88	7	4.56E6	1	15099	Cytochrome c555 OS=Candidatus Accumulibacter sp. BA-93 OX=1454004 GN=AW11_00070 PE=4 SV=1
A0A011QJD6	57.88	7	4.56E6	1	15099	Cytochrome c555 OS=Candidatus Accumulibacter sp. BA-92 OX=1454003 GN=AW10_02585 PE=4 SV=1
A0A1A8XI19	57.69	3	1.03E6	1	58210	L-aspartate oxidase OS=Candidatus Accumulibacter aalborgensis OX=1860102 GN=nadB PE=3 SV=1
A0A011MFU5	57.41	3	0.00E+00	1	97613	Aconitate hydratase OS=Candidatus Accumulibacter sp. SK-12 OX=1454001 GN=acn PE=3 SV=1
A0A011QPF4	56.88	4	0.00E+00	1	33553	Virulence factor Mce family protein OS=Candidatus Accumulibacter sp. BA-93 OX=1454004 GN=AW11_00291 PE=4 SV=1

A0A080M2M8	56.74	2	0.00E+00	1	63843	Sulfite reductase [ferredoxin] OS=Candidatus Accumulibacter sp. BA-91 OX=1454002 GN=sir PE=4 SV=1
A0A084XWU5	56.74	2	0.00E+00	1	64012	Sulfite reductase [ferredoxin] OS=Candidatus Accumulibacter sp. SK-01 OX=1457154 GN=sir PE=4 SV=1
A0A080M7B9	56.74	2	0.00E+00	1	63994	Sulfite reductase [ferredoxin] OS=Candidatus Accumulibacter sp. SK-02 OX=1453999 GN=sir PE=4 SV=1
A0A369XKS3	56.57	9	3.04E7	1	12953	Uncharacterized protein OS=Candidatus Accumulibacter phosphatis OX=327160 GN=DVS81_09710 PE=4 SV=1
A0A011NXT6	56.53	3	0.00E+00	1	50054	Dihydrolipoyl dehydrogenase OS=Candidatus Accumulibacter sp. BA-93 OX=1454004 GN=lpdV PE=4 SV=1
A0A011RE32	56.29	5	0.00E+00	1	28131	Uncharacterized protein OS=Candidatus Accumulibacter sp. BA-93 OX=1454004 GN=AW11_01555 PE=4 SV=1
A0A011NZC1	56.24	2	1.22E6	1	58447	Long-chain-fatty-acid--CoA ligase OS=Candidatus Accumulibacter sp. BA-93 OX=1454004 GN=lcfB_1 PE=4 SV=1
A0A369XGA3	55.73	5	0.00E+00	1	28731	ParA family protein OS=Candidatus Accumulibacter phosphatis OX=327160 GN=DVS81_18555 PE=4 SV=1
A0A011PJK3	55.73	5	0.00E+00	1	28885	Sporulation initiation inhibitor protein soj OS=Candidatus Accumulibacter sp. BA-92 OX=1454003 GN=soj_5 PE=4 SV=1
A0A011NXK0	55.73	5	0.00E+00	1	28737	Sporulation initiation inhibitor protein soj OS=Candidatus Accumulibacter sp. BA-93 OX=1454004 GN=soj_4 PE=4 SV=1
A0A080M214	55.36	9	0.00E+00	1	32058	Inositol-1-monophosphatase OS=Candidatus Accumulibacter sp. BA-91 OX=1454002 GN=suhB_2 PE=4 SV=1
A0A011QJ56	55.36	9	0.00E+00	1	32318	Inositol-1-monophosphatase OS=Candidatus Accumulibacter sp. BA-93 OX=1454004 GN=suhB_2 PE=4 SV=1
A0A011Q984	54.83	4	0.00E+00	1	35580	Putative ATPase (AAA+ superfamily) OS=Candidatus Accumulibacter sp. BA-93 OX=1454004 GN=AW11_03377 PE=4 SV=1
A0A369XQW7	54.83	4	0.00E+00	1	35242	ATP-binding protein OS=Candidatus Accumulibacter phosphatis OX=327160 GN=DVS81_07505 PE=4 SV=1
A0A1A8XY15	54.37	4	5.17E6	1	28196	ATP synthase subunit a OS=Candidatus Accumulibacter aalborgensis OX=1860102 GN=atpB PE=3 SV=1
A0A011PKS6	54.31	6	0.00E+00	1	17444	Ribbon-helix-helix protein copG family OS=Candidatus Accumulibacter sp. BA-93 OX=1454004 GN=AW11_02229 PE=4 SV=1
A0A011QPL0	53.63	9	0.00E+00	1	16546	Uncharacterized protein OS=Candidatus Accumulibacter sp. BA-93 OX=1454004 GN=AW11_00321 PE=4 SV=1
A0A011QYS0	53.56	8	2.77E7	1	14984	Cobalamin biosynthesis protein CbiG OS=Candidatus Accumulibacter sp. BA-93 OX=1454004 GN=AW11_04041 PE=4 SV=1
A0A011QDW1	53.26	10	0.00E+00	1	11445	Iron-sulfur cluster assembly protein CyaY OS=Candidatus Accumulibacter sp. BA-93 OX=1454004 GN=cyaY PE=3 SV=1
A0A369XK93	53.25	3	0.00E+00	1	52764	Carbon starvation protein A OS=Candidatus Accumulibacter phosphatis OX=327160 GN=DVS81_20875 PE=4 SV=1
A0A011QGR0	53.04	6	6.4E6	1	24921	Uncharacterized protein OS=Candidatus Accumulibacter sp. BA-93 OX=1454004 GN=AW11_02087 PE=4 SV=1
A0A011QP72	52.91	16	1.92E6	1	18651	Transcription antitermination protein RfaH OS=Candidatus Accumulibacter sp. BA-93 OX=1454004 GN=rfaH PE=4 SV=1

A0A369XUA0	52.73	4	0.00E+00	1	38974	Uncharacterized protein OS=Candidatus Accumulibacter phosphatis OX=327160 GN=DVS81_03840 PE=4 SV=1
A0A011QCS5	52.71	15	0.00E+00	1	13601	Uncharacterized protein OS=Candidatus Accumulibacter sp. BA-93 OX=1454004 GN=AW11_02883 PE=4 SV=1
A0A011QJG7	52.70	3	0.00E+00	1	42863	Acyl-CoA dehydrogenase OS=Candidatus Accumulibacter sp. BA-93 OX=1454004 GN=acdA_1 PE=3 SV=1
A0A011N4Y6	52.39	8	0.00E+00	1	26499	5'-nucleotidase SurE OS=Candidatus Accumulibacter sp. BA-92 OX=1454003 GN=surE PE=3 SV=1
A0A011NLP1	52.39	8	0.00E+00	1	26310	5'-nucleotidase SurE OS=Candidatus Accumulibacter sp. SK-12 OX=1454001 GN=surE PE=3 SV=1
A0A011Q4G9	52.39	8	0.00E+00	1	26686	5'-nucleotidase SurE OS=Candidatus Accumulibacter sp. BA-93 OX=1454004 GN=surE PE=3 SV=1
A0A1A8XHN1	52.39	8	0.00E+00	1	26442	5'-nucleotidase SurE OS=Candidatus Accumulibacter aalborgensis OX=1860102 GN=surE PE=3 SV=1
A0A084Y414	52.39	8	0.00E+00	1	26402	5'-nucleotidase SurE OS=Candidatus Accumulibacter sp. SK-01 OX=1457154 GN=surE PE=3 SV=1
A0A369XP03	52.39	8	0.00E+00	1	26521	5'/3'-nucleotidase SurE OS=Candidatus Accumulibacter phosphatis OX=327160 GN=DVS81_04215 PE=4 SV=1
A0A369XWL5	52.36	11	0.00E+00	1	24302	Type IV secretion protein Rhs OS=Candidatus Accumulibacter phosphatis OX=327160 GN=DVS81_03845 PE=4 SV=1
A0A011QJ62	52.09	3	0.00E+00	1	52528	Magnesium and cobalt efflux protein CorC OS=Candidatus Accumulibacter sp. BA-93 OX=1454004 GN=corC_4 PE=4 SV=1
A0A011PP79	51.87	4	0.00E+00	1	31563	Arogenate dehydrogenase OS=Candidatus Accumulibacter sp. BA-93 OX=1454004 GN=tyrC PE=4 SV=1
A0A1A8XHS2	51.70	2	0.00E+00	1	89572	Arylsulfatase A family protein OS=Candidatus Accumulibacter aalborgensis OX=1860102 GN=ACCAA_1050003 PE=4 SV=1
A0A011R2M9	51.15	4	0.00E+00	1	46961	Glutamate-aspartate carrier protein OS=Candidatus Accumulibacter sp. BA-93 OX=1454004 GN=gltT PE=3 SV=1
A0A011QHG2	50.94	2	0.00E+00	1	43799	LL-diaminopimelate aminotransferase OS=Candidatus Accumulibacter sp. BA-92 OX=1454003 GN=dapL PE=4 SV=1
A0A011QKK2	50.82	7	0.00E+00	1	16940	Flagella synthesis chaperone protein FlgN OS=Candidatus Accumulibacter sp. BA-93 OX=1454004 GN=AW11_01371 PE=4 SV=1
A0A369XLD9	50.42	6	0.00E+00	1	40327	tRNA 2-thiouridine(34) synthase MnmA OS=Candidatus Accumulibacter phosphatis OX=327160 GN=DVS81_12925 PE=4 SV=1
A0A011MC54	50.26	3	0.00E+00	1	34762	Glutathione synthetase OS=Candidatus Accumulibacter sp. SK-12 OX=1454001 GN=gshB PE=3 SV=1
A0A011NA60	50.24	9	0.00E+00	1	12557	Alkyl hydroperoxide reductase AhpD OS=Candidatus Accumulibacter sp. BA-92 OX=1454003 GN=AW10_02367 PE=3 SV=1
A0A011QP18	50.11	6	0.00E+00	1	21130	Putative hydrolase OS=Candidatus Accumulibacter sp. BA-93 OX=1454004 GN=AW11_00368 PE=4 SV=1
A0A011P4Y9	49.66	5	0.00E+00	1	31039	Uncharacterized protein OS=Candidatus Accumulibacter sp. SK-11 OX=1454000 GN=AW07_03464 PE=4 SV=1
A0A011P510	49.37	9	0.00E+00	1	16094	Uncharacterized protein OS=Candidatus Accumulibacter sp. BA-93 OX=1454004 GN=AW11_01170 PE=4 SV=1
A0A369XFA7	49.37	9	0.00E+00	1	15940	Uncharacterized protein OS=Candidatus Accumulibacter phosphatis OX=327160 GN=DVS81_20245 PE=4 SV=1

A0A011QP97	49.21	4	0.00E+00	1	44904	Uncharacterized protein OS=Candidatus Accumulibacter sp. BA-93 OX=1454004 GN=AW11_00246 PE=4 SV=1
A0A011P929	49.00	12	0.00E+00	1	16597	Uncharacterized protein OS=Candidatus Accumulibacter sp. BA-93 OX=1454004 GN=AW11_04020 PE=4 SV=1
A0A011NP98	49.00	12	0.00E+00	1	16827	Uncharacterized protein OS=Candidatus Accumulibacter sp. BA-92 OX=1454003 GN=AW10_03996 PE=4 SV=1
A0A011PQN7	48.60	3	0.00E+00	1	29457	Ferredoxin--NADP reductase OS=Candidatus Accumulibacter sp. BA-93 OX=1454004 GN=fpr_1 PE=4 SV=1
A0A011QTN1	48.60	3	0.00E+00	1	29513	Ferredoxin--NADP reductase OS=Candidatus Accumulibacter sp. BA-92 OX=1454003 GN=fpr_1 PE=4 SV=1
A0A369XLY8	48.60	3	0.00E+00	1	29387	Ferredoxin--NADP reductase OS=Candidatus Accumulibacter phosphatis OX=327160 GN=DVS81_11765 PE=4 SV=1
A0A1Q3VV69	48.57	13	0.00E+00	1	14073	Protein ApaG OS=Candidatus Accumulibacter sp. 66-26 OX=1895689 GN=apaG PE=3 SV=1
A0A011PHG5	48.53	4	0.00E+00	1	27628	Molybdate-binding periplasmic protein OS=Candidatus Accumulibacter sp. BA-93 OX=1454004 GN=modA PE=4 SV=1
A0A369XL33	48.53	4	0.00E+00	1	27848	Molybdate ABC transporter substrate-binding protein OS=Candidatus Accumulibacter phosphatis OX=327160 GN=modA PE=4 SV=1
A0A011QIE4	48.53	4	0.00E+00	1	27820	Molybdate-binding periplasmic protein OS=Candidatus Accumulibacter sp. BA-92 OX=1454003 GN=modA PE=4 SV=1
A0A011PFH4	48.42	2	3.89E7	1	52858	Phosphate transporter OS=Candidatus Accumulibacter sp. BA-93 OX=1454004 GN=cysP PE=3 SV=1
A0A011NY42	48.42	2	3.89E7	1	52731	Phosphate transporter OS=Candidatus Accumulibacter sp. BA-92 OX=1454003 GN=cysP PE=3 SV=1
A0A011PLB2	48.32	3	6.75E5	1	54584	Uncharacterized protein OS=Candidatus Accumulibacter sp. BA-93 OX=1454004 GN=AW11_02182 PE=4 SV=1
A0A011REE5	48.14	8	0.00E+00	1	14913	Fumarate reductase 15 kDa hydrophobic protein OS=Candidatus Accumulibacter sp. BA-93 OX=1454004 GN=frdC PE=4 SV=1
A0A369XNW7	48.14	8	0.00E+00	1	15027	Fumarate reductase subunit C OS=Candidatus Accumulibacter phosphatis OX=327160 GN=DVS81_04155 PE=4 SV=1
A0A011NU11	48.08	4	0.00E+00	1	27659	Putative periplasmic serine endoprotease DegP-like OS=Candidatus Accumulibacter sp. SK-12 OX=1454001 GN=mucD_2 PE=4 SV=1
A0A080LTH1	48.08	4	0.00E+00	1	29587	Putative periplasmic serine endoprotease DegP-like OS=Candidatus Accumulibacter sp. BA-91 OX=1454002 GN=mucD_2 PE=4 SV=1
A0A011PC90	48.08	5	0.00E+00	1	20034	Peptidase Do OS=Candidatus Accumulibacter sp. BA-94 OX=1454005 GN=AW12_00445 PE=4 SV=1
C7RIG7	48.08	4	0.00E+00	1	27213	Peptidase S1 and S6 chymotrypsin/Hap OS=Accumulibacter phosphatis (strain UW-1) OX=522306 GN=CAP2UW1_3276 PE=4 SV=1
A0A011QP09	48.08	3	0.00E+00	1	30976	Putative periplasmic serine endoprotease DegP-like OS=Candidatus Accumulibacter sp. BA-93 OX=1454004 GN=mucD_1 PE=4 SV=1
A0A1A8XMC8	48.08	3	0.00E+00	1	30485	Peptidase S1 and S6 chymotrypsin/Hap OS=Candidatus Accumulibacter aalborgensis OX=1860102 GN=ACCAA_220055 PE=4 SV=1
A0A1A8XLQ7	48.04	14	0.00E+00	1	9182	Uncharacterized protein OS=Candidatus Accumulibacter aalborgensis OX=1860102 GN=ACCAA_20011 PE=4 SV=1

C7RUU1	48.04	14	0.00E+00	1	8867	Uncharacterized protein OS=Accumulibacter phosphatis (strain UW-1) OX=522306 GN=CAP2UW1_3063 PE=4 SV=1
A0A080M679	48.04	14	0.00E+00	1	9188	Uncharacterized protein OS=Candidatus Accumulibacter sp. SK-02 OX=1453999 GN=AW06_002925 PE=4 SV=1
A0A369XP95	48.04	14	0.00E+00	1	9056	Uncharacterized protein OS=Candidatus Accumulibacter phosphatis OX=327160 GN=DVS81_13290 PE=4 SV=1
A0A011NTI0	48.04	14	0.00E+00	1	9129	Uncharacterized protein OS=Candidatus Accumulibacter sp. BA-93 OX=1454004 GN=AW11_03247 PE=4 SV=1
A0A011M5T2	48.04	14	0.00E+00	1	9164	Uncharacterized protein OS=Candidatus Accumulibacter sp. SK-12 OX=1454001 GN=AW08_03571 PE=4 SV=1
A0A084XZC1	48.04	12	0.00E+00	1	10617	Uncharacterized protein OS=Candidatus Accumulibacter sp. SK-01 OX=1457154 GN=CAPSK01_002403 PE=4 SV=1
A0A011N5J8	47.46	13	0.00E+00	1	9033	Sec-independent protein translocase protein TatA OS=Candidatus Accumulibacter sp. BA-92 OX=1454003 GN=tatA PE=3 SV=1
A0A369XPP5	47.46	13	0.00E+00	1	9123	Sec-independent protein translocase subunit TatA OS=Candidatus Accumulibacter phosphatis OX=327160 GN=DVS81_09815 PE=4 SV=1
A0A011P370	47.46	13	0.00E+00	1	8983	Sec-independent protein translocase protein TatA OS=Candidatus Accumulibacter sp. BA-93 OX=1454004 GN=tatA PE=3 SV=1
A0A011PMN8	46.94	13	0.00E+00	1	10036	Uncharacterized protein OS=Candidatus Accumulibacter sp. BA-93 OX=1454004 GN=AW11_02010 PE=4 SV=1
A0A011P0P8	46.58	4	0.00E+00	1	34934	Putative glycosyltransferase EpsJ OS=Candidatus Accumulibacter sp. BA-93 OX=1454004 GN=epsJ PE=4 SV=1
A0A011PIS6	46.27	4	0.00E+00	1	39065	3-isopropylmalate dehydrogenase OS=Candidatus Accumulibacter sp. BA-93 OX=1454004 GN=leuB_2 PE=4 SV=1
A0A011QDN0	46.16	3	0.00E+00	1	49413	TRAP transporter solute receptor TAXI family OS=Candidatus Accumulibacter sp. BA-93 OX=1454004 GN=AW11_02535 PE=4 SV=1
A0A011QG50	46.09	14	0.00E+00	1	12246	Membrane fusogenic activity OS=Candidatus Accumulibacter sp. BA-93 OX=1454004 GN=AW11_02304 PE=4 SV=1
A0A011QM20	45.66	5	0.00E+00	1	25914	CRISPR system Cascade subunit CasE OS=Candidatus Accumulibacter sp. BA-93 OX=1454004 GN=casE PE=4 SV=1
A0A011Q796	45.63	4	1.26E6	1	33259	50S ribosomal protein L3 glutamine methyltransferase OS=Candidatus Accumulibacter sp. BA-93 OX=1454004 GN=prmB PE=3 SV=1
A0A011NW19	45.62	5	0.00E+00	1	27548	Uroporphyrinogen-III synthase OS=Candidatus Accumulibacter sp. BA-92 OX=1454003 GN=hemD PE=4 SV=1
A0A369XGZ0	45.62	5	0.00E+00	1	27593	Uroporphyrinogen-III synthase OS=Candidatus Accumulibacter phosphatis OX=327160 GN=DVS81_18390 PE=4 SV=1
A0A011NQV9	45.55	5	0.00E+00	1	26661	Putative transmembrane sensor domain protein OS=Candidatus Accumulibacter sp. BA-93 OX=1454004 GN=AW11_03697 PE=4 SV=1
A0A011Q7N9	45.45	15	0.00E+00	1	8958	Transcriptional regulator BolA OS=Candidatus Accumulibacter sp. BA-93 OX=1454004 GN=AW11_03585 PE=3 SV=1

A0A1A8XYG5	45.30	2	0.00E+00	1	59774	Sulphate transporter OS=Candidatus Accumulibacter aalborgensis OX=1860102 GN=ACCAA_810036 PE=3 SV=1
A0A011QRD6	45.30	2	0.00E+00	1	60511	Putative sulfate transporterc OS=Candidatus Accumulibacter sp. BA-92 OX=1454003 GN=AW10_01156 PE=3 SV=1
A0A011PQ31	45.30	2	0.00E+00	1	60414	Putative sulfate transporterc OS=Candidatus Accumulibacter sp. BA-93 OX=1454004 GN=AW11_01403 PE=3 SV=1
A0A084Y4B0	45.30	2	0.00E+00	1	61822	Putative sulfate transporterc OS=Candidatus Accumulibacter sp. SK-01 OX=1457154 GN=CAPSK01_000817 PE=3 SV=1
A0A080M917	45.30	2	0.00E+00	1	61532	Putative sulfate transporterc OS=Candidatus Accumulibacter sp. SK-02 OX=1453999 GN=AW06_001963 PE=3 SV=1
A0A369XN10	45.16	4	0.00E+00	1	35229	Complex I NDUFA9 subunit family protein OS=Candidatus Accumulibacter phosphatis OX=327160 GN=DVS81_05105 PE=4 SV=1
A0A011PJL6	43.88	6	0.00E+00	1	20014	Uncharacterized protein OS=Candidatus Accumulibacter sp. BA-93 OX=1454004 GN=AW11_02401 PE=4 SV=1
A0A369XJH0	43.88	5	0.00E+00	1	22168	DUF3299 domain-containing protein OS=Candidatus Accumulibacter phosphatis OX=327160 GN=DVS81_19320 PE=4 SV=1
C7RS80	43.68	10	2.77E6	1	23675	Precorrin-8X methylmutase CbiC/CobH OS=Accumulibacter phosphatis (strain UW-1) OX=522306 GN=CAP2UW1_2617 PE=4 SV=1
A0A011PSZ9	43.61	2	0.00E+00	1	75931	NADH-quinone oxidoreductase subunit L OS=Candidatus Accumulibacter sp. BA-93 OX=1454004 GN=nuoL_1 PE=4 SV=1
A0A011P8Q0	43.47	4	0.00E+00	1	25650	3-oxoacyl-[acyl-carrier-protein] reductase FabG OS=Candidatus Accumulibacter sp. BA-93 OX=1454004 GN=fabG_1 PE=4 SV=1
A0A011QE38	42.81	9	4.5E6	1	15630	Uncharacterized protein OS=Candidatus Accumulibacter sp. BA-93 OX=1454004 GN=AW11_02466 PE=4 SV=1
A0A011QIP3	42.81	9	4.5E6	1	15699	Uncharacterized protein OS=Candidatus Accumulibacter sp. BA-92 OX=1454003 GN=AW10_02873 PE=4 SV=1
A0A011PS47	42.75	2	0.00E+00	1	54543	Glycerol kinase OS=Candidatus Accumulibacter sp. BA-93 OX=1454004 GN=glpK PE=3 SV=1
C7RNC2	42.06	6	5.61E6	1	30672	Nitrilase/cyanide hydratase and apolipoprotein N-acyltransferase OS=Accumulibacter phosphatis (strain UW-1) OX=522306 GN=CAP2UW1_4066 PE=4 SV=1
A0A011NX14	41.85	12	0.00E+00	1	12793	Uncharacterized protein OS=Candidatus Accumulibacter sp. BA-93 OX=1454004 GN=AW11_02675 PE=4 SV=1
A0A011QQ22	41.81	6	0.00E+00	1	28407	Trans-aconitate 2-methyltransferase OS=Candidatus Accumulibacter sp. BA-93 OX=1454004 GN=tam PE=3 SV=1
A0A1Q3VN06	41.68	6	0.00E+00	1	40469	Acetyl-CoA acetyltransferase OS=Candidatus Accumulibacter sp. 66-26 OX=1895689 GN=BGO63_10025 PE=3 SV=1
A0A011QHK0	41.66	3	0.00E+00	1	36078	Histidinol phosphatase of the PHP family protein OS=Candidatus Accumulibacter sp. BA-93 OX=1454004 GN=AW11_01905 PE=4 SV=1
A0A369XGR0	41.66	3	0.00E+00	1	33081	PHP domain-containing protein OS=Candidatus Accumulibacter phosphatis OX=327160 GN=DVS81_18810 PE=4 SV=1
A0A011PP12	41.61	4	0.00E+00	1	41039	Leucine ABC transporter subunit substrate-binding protein LivK OS=Candidatus Accumulibacter sp. BA-93 OX=1454004 GN=AW11_01716 PE=4 SV=1

A0A011QKD9	41.61	4	0.00E+00	1	40658	Leucine ABC transporter subunit substrate-binding protein LivK OS=Candidatus Accumulibacter sp. BA-92 OX=1454003 GN=AW10_02442 PE=4 SV=1
A0A080M2F5	41.25	3	0.00E+00	1	38899	S-(hydroxymethyl)glutathione dehydrogenase OS=Candidatus Accumulibacter sp. SK-02 OX=1453999 GN=frmA PE=3 SV=1
A0A011QB73	41.21	4	8.01E6	1	24494	Uncharacterized protein OS=Candidatus Accumulibacter sp. BA-93 OX=1454004 GN=AW11_03149 PE=4 SV=1
A0A011QJM8	41.02	12	0.00E+00	1	12091	Uncharacterized protein OS=Candidatus Accumulibacter sp. BA-93 OX=1454004 GN=AW11_01434 PE=3 SV=1
A0A369XRW3	40.91	9	0.00E+00	1	16891	Hemerythrin domain-containing protein OS=Candidatus Accumulibacter phosphatis OX=327160 GN=DVS81_08665 PE=4 SV=1
A0A011QAZ6	40.91	8	0.00E+00	1	17415	Iron-sulfur cluster repair di-iron protein OS=Candidatus Accumulibacter sp. BA-93 OX=1454004 GN=AW11_03186 PE=4 SV=1
A0A011PAK7	39.96	6	2.38E6	1	27169	Putative HTH-type transcriptional regulator YurK OS=Candidatus Accumulibacter sp. BA-93 OX=1454004 GN=yurK PE=4 SV=1
A0A011PRL5	38.85	2	0.00E+00	1	99639	Sensor histidine kinase YycG OS=Candidatus Accumulibacter sp. BA-93 OX=1454004 GN=yycG_1 PE=4 SV=1
C7RVD7	38.85	2	0.00E+00	1	99584	Integral membrane sensor signal transduction histidine kinase OS=Accumulibacter phosphatis (strain UW-1) OX=522306 GN=CAP2UW1_3138 PE=4 SV=1
A0A011QNN0	38.54	6	0.00E+00	1	21180	Cytochrome c biogenesis protein TlpA OS=Candidatus Accumulibacter sp. BA-93 OX=1454004 GN=tlpA_1 PE=4 SV=1
A0A080M7W2	38.49	4	0.00E+00	1	38751	Glutamyl-tRNA(Gln) amidotransferase subunit A OS=Candidatus Accumulibacter sp. BA-91 OX=1454002 GN=gatA PE=3 SV=1
A0A011NR19	38.38	11	0.00E+00	1	18073	6 7-dimethyl-8-ribityllumazine synthase OS=Candidatus Accumulibacter sp. BA-93 OX=1454004 GN=ribH PE=3 SV=1
A0A011Q7Y1	37.98	10	2.47E6	1	10022	Putative NTP binding protein (Contains STAS domain) OS=Candidatus Accumulibacter sp. BA-93 OX=1454004 GN=AW11_03588 PE=4 SV=1
A0A011PUB6	37.44	2	0.00E+00	1	59736	Multifunctional fusion protein OS=Candidatus Accumulibacter sp. BA-93 OX=1454004 GN=nnr PE=3 SV=1
A0A1Q3VPP6	37.44	2	0.00E+00	1	51441	Multifunctional fusion protein OS=Candidatus Accumulibacter sp. 66-26 OX=1895689 GN=nnrE PE=3 SV=1
A0A011P0Q9	37.41	5	0.00E+00	1	22305	Uncharacterized protein OS=Candidatus Accumulibacter sp. BA-93 OX=1454004 GN=AW11_02168 PE=4 SV=1
C7RVS7	37.33	8	0.00E+00	1	15378	Uncharacterized protein OS=Accumulibacter phosphatis (strain UW-1) OX=522306 GN=CAP2UW1_4604 PE=4 SV=1
A0A011P442	36.85	4	0.00E+00	1	33750	Metal-dependent hydrolase OS=Candidatus Accumulibacter sp. BA-92 OX=1454003 GN=AW10_00474 PE=4 SV=1
A0A011P487	36.69	7	0.00E+00	1	15778	2-C-methyl-D-erythritol 4-phosphate cytidylyltransferase OS=Candidatus Accumulibacter sp. BA-93 OX=1454004 GN=ispD_1 PE=3 SV=1
A0A011NNX7	36.69	6	0.00E+00	1	16958	2-C-methyl-D-erythritol 4-phosphate cytidylyltransferase OS=Candidatus Accumulibacter sp. BA-92 OX=1454003 GN=ispD_2 PE=3 SV=1
A0A011PER8	36.40	13	0.00E+00	1	8931	Trafficking protein A OS=Candidatus Accumulibacter sp. BA-92 OX=1454003 GN=fitA_2 PE=4 SV=1

A0A011QML0	36.40	12	0.00E+00	1	9404	Trafficking protein A OS=Candidatus Accumulibacter sp. BA-93 OX=1454004 GN=fitA PE=4 SV=1
A0A1A8XI59	36.40	12	0.00E+00	1	9387	Putative plasmid stability protein y4jJ OS=Candidatus Accumulibacter aalborgensis OX=1860102 GN=ACCAA_130037 PE=4 SV=1
A0A369XH07	36.40	12	0.00E+00	1	9451	Plasmid stabilization protein OS=Candidatus Accumulibacter phosphatis OX=327160 GN=DVS81_18210 PE=4 SV=1
A0A011RGY5	36.34	3	5.43E6	1	27855	4-hydroxy-tetrahydrodipicolinate reductase OS=Candidatus Accumulibacter sp. BA-93 OX=1454004 GN=dapB PE=3 SV=1
A0A011PPA5	36.30	4	3.72E6	1	29843	Isoprenyl transferase OS=Candidatus Accumulibacter sp. BA-93 OX=1454004 GN=ispU PE=3 SV=1
A0A011PNR6	36.25	9	0.00E+00	1	20041	Uncharacterized protein OS=Candidatus Accumulibacter sp. BA-93 OX=1454004 GN=AW11_01818 PE=4 SV=1
A0A080M4L6	35.96	11	0.00E+00	1	20423	Peptidyl-prolyl cis-trans isomerase OS=Candidatus Accumulibacter sp. BA-91 OX=1454002 GN=AW09_002814 PE=3 SV=1
A0A080M595	35.96	11	0.00E+00	1	20490	Peptidyl-prolyl cis-trans isomerase OS=Candidatus Accumulibacter sp. SK-02 OX=1453999 GN=AW06_002754 PE=3 SV=1
A0A084XUX6	35.96	11	0.00E+00	1	20623	Peptidyl-prolyl cis-trans isomerase OS=Candidatus Accumulibacter sp. SK-01 OX=1457154 GN=CAPSK01_004431 PE=3 SV=1
A0A011PM58	35.76	4	0.00E+00	1	28090	Iron(3+)-hydroxamate import ATP-binding protein FhuC OS=Candidatus Accumulibacter sp. BA-93 OX=1454004 GN=fhuC PE=4 SV=1
A0A011NH33	35.76	4	0.00E+00	1	28479	Iron(3+)-hydroxamate import ATP-binding protein FhuC OS=Candidatus Accumulibacter sp. BA-92 OX=1454003 GN=fhuC PE=4 SV=1
A0A080M2I4	35.76	4	0.00E+00	1	27813	Iron(3+)-hydroxamate import ATP-binding protein FhuC OS=Candidatus Accumulibacter sp. SK-02 OX=1453999 GN=fhuC PE=4 SV=1
C7RM72	35.76	4	0.00E+00	1	28063	ABC transporter related OS=Accumulibacter phosphatis (strain UW-1) OX=522306 GN=CAP2UW1_3900 PE=4 SV=1
A0A369XNZ2	35.76	4	0.00E+00	1	28225	ABC transporter ATP-binding protein OS=Candidatus Accumulibacter phosphatis OX=327160 GN=DVS81_13805 PE=4 SV=1
A0A1A8XN95	35.76	4	0.00E+00	1	27642	ABC transporter related OS=Candidatus Accumulibacter aalborgensis OX=1860102 GN=ACCAA_210010 PE=4 SV=1
A0A1Q3VTE1	35.76	4	0.00E+00	1	27938	ABC transporter ATP-binding protein OS=Candidatus Accumulibacter sp. 66-26 OX=1895689 GN=BGO63_09205 PE=4 SV=1
A0A1A8XG22	35.65	2	0.00E+00	1	47210	Uncharacterized protein OS=Candidatus Accumulibacter aalborgensis OX=1860102 GN=ACCAA_130083 PE=4 SV=1
A0A011PN56	35.48	11	0.00E+00	1	13281	Uncharacterized protein OS=Candidatus Accumulibacter sp. BA-93 OX=1454004 GN=AW11_01984 PE=4 SV=1
A0A011PHW1	35.26	8	0.00E+00	1	23990	Uncharacterized protein OS=Candidatus Accumulibacter sp. SK-11 OX=1454000 GN=AW07_00021 PE=4 SV=1
A0A351BKG2	35.26	8	0.00E+00	1	23990	DUF47 domain-containing protein OS=Candidatus Accumulibacter sp. OX=2053492 GN=DCY47_15115 PE=4 SV=1
A0A1Q3VR19	35.25	3	0.00E+00	1	45179	Diaminopimelate decarboxylase OS=Candidatus Accumulibacter sp. 66-26 OX=1895689 GN=lysA PE=3 SV=1

A0A011NPK1	35.01	3	0.00E+00	1	44233	L-glutamyl-[BtrI acyl-carrier protein] decarboxylase OS=Candidatus Accumulibacter sp. BA-92 OX=1454003 GN=btrK PE=3 SV=1
A0A369XFQ0	34.99	5	0.00E+00	1	22177	Heme-binding protein OS=Candidatus Accumulibacter phosphatis OX=327160 GN=DVS81_19560 PE=4 SV=1
A0A011P230	34.99	12	0.00E+00	1	10719	SOUL heme-binding protein OS=Candidatus Accumulibacter sp. BA-94 OX=1454005 GN=AW12_02763 PE=4 SV=1
A0A011NX57	34.99	5	0.00E+00	1	22371	SOUL heme-binding protein OS=Candidatus Accumulibacter sp. BA-93 OX=1454004 GN=AW11_02641 PE=4 SV=1
A0A011PW56	34.99	5	0.00E+00	1	25577	SOUL heme-binding protein OS=Candidatus Accumulibacter sp. BA-92 OX=1454003 GN=AW10_01413 PE=4 SV=1
A0A011Q3V2	34.96	6	0.00E+00	1	25980	Cytochrome c1 OS=Candidatus Accumulibacter sp. BA-94 OX=1454005 GN=petC PE=4 SV=1
A0A011NX51	34.80	1	0.00E+00	1	103666	Uncharacterized protein OS=Candidatus Accumulibacter sp. BA-92 OX=1454003 GN=AW10_02195 PE=4 SV=1
A0A011PSX4	34.76	0	0.00E+00	1	1302526	Chitinase A OS=Candidatus Accumulibacter sp. BA-92 OX=1454003 GN=chiA PE=4 SV=1
A0A011NAP5	33.96	5	0.00E+00	1	72563	Long-chain-fatty-acid--CoA ligase FadD15 OS=Candidatus Accumulibacter sp. BA-92 OX=1454003 GN=AW10_02218 PE=4 SV=1
A0A011PZC8	33.69	4	0.00E+00	1	20056	Leucine-responsive regulatory protein OS=Candidatus Accumulibacter sp. BA-92 OX=1454003 GN=lrp_1 PE=4 SV=1
A0A011QGD0	33.67	15	0.00E+00	1	9389	DNA binding domain excisionase family OS=Candidatus Accumulibacter sp. BA-93 OX=1454004 GN=AW11_02233 PE=4 SV=1
A0A011NMU0	33.44	5	0.00E+00	1	23455	Uncharacterized protein OS=Candidatus Accumulibacter sp. BA-93 OX=1454004 GN=AW11_03982 PE=4 SV=1
A0A011NZM3	33.44	5	0.00E+00	1	23582	Uncharacterized protein OS=Candidatus Accumulibacter sp. BA-92 OX=1454003 GN=AW10_01429 PE=4 SV=1
A0A011NMQ6	32.59	3	0.00E+00	1	25431	Cobalamin biosynthesis protein CbiG OS=Candidatus Accumulibacter sp. BA-93 OX=1454004 GN=AW11_04040 PE=4 SV=1
A0A011R4I5	32.09	13	0.00E+00	1	14788	Uncharacterized protein OS=Candidatus Accumulibacter sp. BA-93 OX=1454004 GN=AW11_03253 PE=4 SV=1
C7RVP8	31.26	8	0.00E+00	1	23663	Uncharacterized protein OS=Accumulibacter phosphatis (strain UW-1) OX=522306 GN=CAP2UW1_4574 PE=4 SV=1
A0A011PLL2	31.00	3	1.03E6	1	39602	Putative inner membrane protein OS=Candidatus Accumulibacter sp. BA-92 OX=1454003 GN=AW10_03578 PE=4 SV=1
C7RSR0	30.52	2	0.00E+00	1	49066	ATP synthase F1 beta subunit OS=Accumulibacter phosphatis (strain UW-1) OX=522306 GN=CAP2UW1_2676 PE=4 SV=1
A0A1Q3VW96	30.52	2	0.00E+00	1	50458	F0F1 ATP synthase subunit beta OS=Candidatus Accumulibacter sp. 66-26 OX=1895689 GN=BGO63_18440 PE=4 SV=1
A0A369XUC0	30.52	2	0.00E+00	1	49512	F0F1 ATP synthase subunit beta OS=Candidatus Accumulibacter phosphatis OX=327160 GN=DVS81_08300 PE=4 SV=1
A0A011PRN4	30.52	2	0.00E+00	1	49654	ATP synthase subunit beta OS=Candidatus Accumulibacter sp. BA-93 OX=1454004 GN=atpD_2 PE=4 SV=1

A0A011PUJ7	30.15	1	0.00E+00	1	137208	Uncharacterized protein OS=Candidatus Accumulibacter sp. SK-11 OX=1454000 GN=AW07_04178 PE=4 SV=1
A0A011PSQ4	29.97	3	0.00E+00	1	30806	4-hydroxy-tetrahydrodipicolinate synthase OS=Candidatus Accumulibacter sp. SK-12 OX=1454001 GN=dapA PE=3 SV=1
A0A011P3Q3	29.83	5	0.00E+00	1	21313	dITP/XTP pyrophosphatase OS=Candidatus Accumulibacter sp. BA-93 OX=1454004 GN=AW11_01445 PE=3 SV=1
A0A011R3N1	29.31	2	0.00E+00	1	32109	33 kDa chaperonin OS=Candidatus Accumulibacter sp. BA-93 OX=1454004 GN=hslO PE=3 SV=1
A0A011PSS5	28.64	8	0.00E+00	1	20026	Uncharacterized protein OS=Candidatus Accumulibacter sp. BA-92 OX=1454003 GN=AW10_02020 PE=4 SV=1
A0A369XSG5	28.64	8	0.00E+00	1	20396	DUF2760 domain-containing protein OS=Candidatus Accumulibacter phosphatis OX=327160 GN=DVS81_11575 PE=4 SV=1
A0A011PT34	28.18	4	0.00E+00	1	31110	4-hydroxy-tetrahydrodipicolinate synthase OS=Candidatus Accumulibacter sp. BA-94 OX=1454005 GN=dapA PE=3 SV=1
A0A1Q3VPK7	27.30	2	0.00E+00	1	57379	Chemotaxis protein OS=Candidatus Accumulibacter sp. 66-26 OX=1895689 GN=BGO63_04475 PE=4 SV=1
A0A351BLP7	26.34	5	0.00E+00	1	27762	Acyl-ACP--UDP-N-acetylglucosamine O-acyltransferase OS=Candidatus Accumulibacter sp. OX=2053492 GN=DCY47_17425 PE=4 SV=1
A0A011P9T1	26.34	5	0.00E+00	1	28332	Acyl-[acyl-carrier-protein]--UDP-N-acetylglucosamine O-acyltransferase OS=Candidatus Accumulibacter sp. SK-11 OX=1454000 GN=lpxA PE=3 SV=1
A0A011NHZ1	25.37	2	0.00E+00	1	35580	Putative GTPase OS=Candidatus Accumulibacter sp. SK-11 OX=1454000 GN=AW07_01472 PE=4 SV=1
A0A1A8XT96	25.21	1	0.00E+00	1	51699	Mannose-1-phosphate guanyltransferase OS=Candidatus Accumulibacter aalborgensis OX=1860102 GN=cpsB PE=3 SV=1
A0A080LZN7	25.21	1	0.00E+00	1	51425	Mannose-1-phosphate guanylyltransferase 1 OS=Candidatus Accumulibacter sp. BA-91 OX=1454002 GN=manC1 PE=3 SV=1
A0A084XWX2	24.73	2	0.00E+00	1	48212	Flagellar M-ring protein OS=Candidatus Accumulibacter sp. SK-01 OX=1457154 GN=fliF_1 PE=4 SV=1
A0A1A8XP68	24.27	2	0.00E+00	1	39734	Phosphoserine transaminase OS=Candidatus Accumulibacter aalborgensis OX=1860102 GN=serC PE=3 SV=1
C7RIY8	23.41	3	0.00E+00	1	42421	UDP-N-acetylglucosamine 2-epimerase OS=Accumulibacter phosphatis (strain UW-1) OX=522306 GN=CAP2UW1_0114 PE=3 SV=1
A0A1Q3VT65	23.41	3	0.00E+00	1	42577	UDP-N-acetylglucosamine 2-epimerase OS=Candidatus Accumulibacter sp. 66-26 OX=1895689 GN=BGO63_08780 PE=3 SV=1
